# Transaminase-Mediated Amine Borrowing *via* Shuttle Biocatalysis

**DOI:** 10.1021/acs.orglett.1c03320

**Published:** 2021-12-15

**Authors:** Freya Taday, James Ryan, Rachel O’Sullivan, Elaine O’Reilly

**Affiliations:** † School of Chemistry, Science Centre South, University College Dublin, Belfield, Dublin 4, Ireland; ‡ School of Chemistry, University of Nottingham, University Park, Nottingham NG7 2RD, U.K.

## Abstract

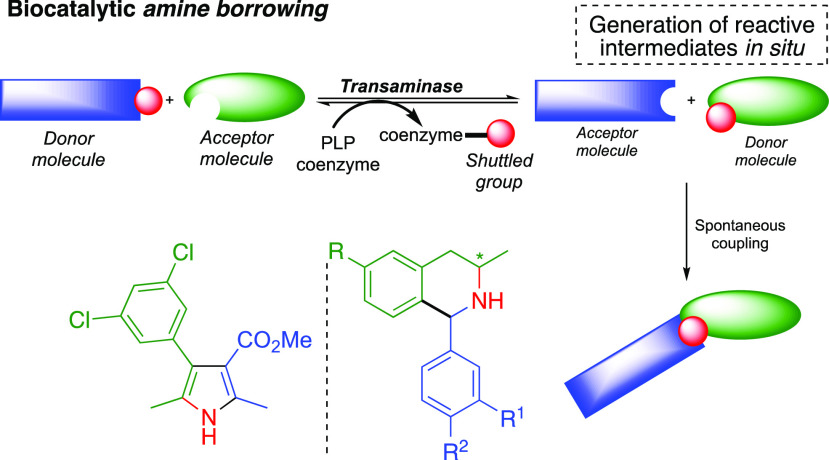

Shuttle catalysis has emerged as a useful methodology for the reversible transfer of small functional groups, such as CO and HCN, and goes far beyond transfer hydrogenation chemistry. While a biocatalytic hydrogen-borrowing methodology is well established, the biocatalytic borrowing of alternative functional groups has not yet been realized. Herein, we present a new concept of *amine borrowing* via biocatalytic shuttle catalysis, which has no counterpart in chemo-shuttle catalysis and allows efficient intermolecular amine shuttling to generate reactive intermediates *in situ*. By coupling this dynamic exchange with an irreversible downstream step to displace the reaction equilibrium in the forward direction, high conversion to target products can be achieved. We showcase the potential of this amine-borrowing methodology using a biocatalytic equivalent of both the Knorr-pyrrole synthesis and Pictet–Spengler reaction.

Shuttle catalysis has emerged as powerful methodology for performing catalytic functional group transfer reactions and relies on the reversible shuttling of functionality between a donor and acceptor molecule (Figure 1a).^1−3^ The methodology is an extension of hydrogen autotransfer,^4,5^ more recently referred to as borrowing hydrogen,^6−8^ and is especially useful when the shuttled group is highly reactive, hazardous, or unstable. In the forward functionalization direction, hazardous reagents (e.g., HCN, syngas) or unstable groups (e.g., HMgBr) can be shuttled *in situ*.^9−11^ In the reverse defunctionalization direction, the approach has been used to valorize biomass and other waste materials and prevent the release of toxic byproducts.^12,13^

**Figure 1:**
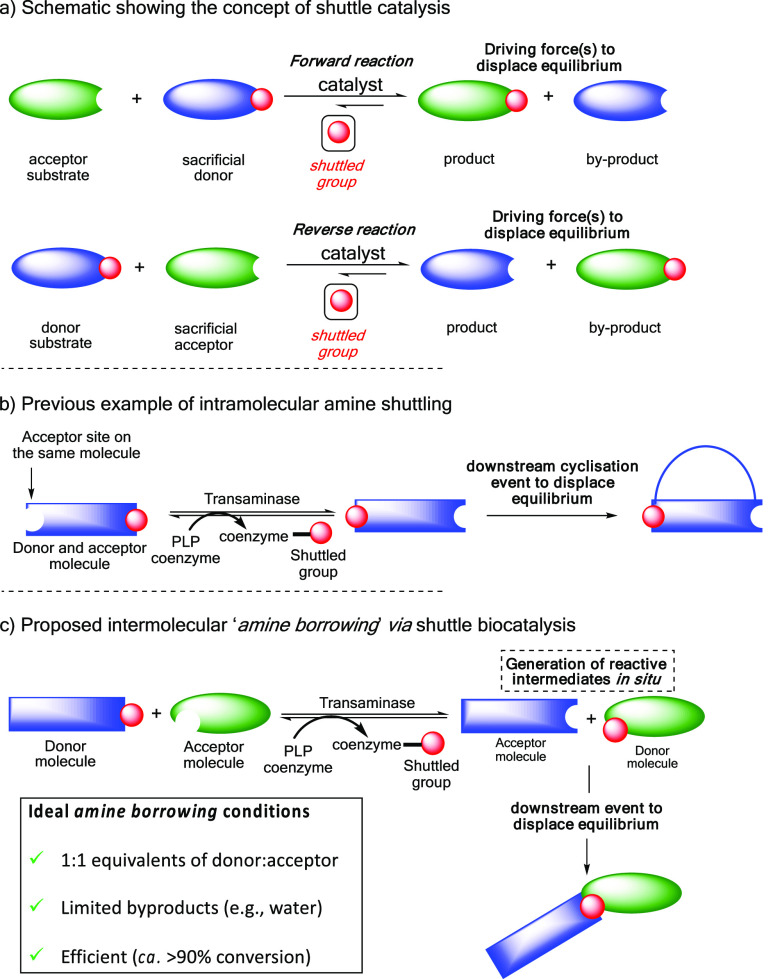
(a) Overall concept of “shuttle catalysis”, where functionality is shuttled *in situ* from a donor to an acceptor molecule, followed by a downstream event to displace the reaction equilibrium. (b) Previous example from our laboratory of intramolecular biocatalytic amine shuttling and spontaneous aza-Michael reaction. (c) Proposed amine-borrowing methodology using PLP to shuttle the amine functionality and generate reactive species *in situ*, which subsequently undergo a downstream event to displace the reaction equilibrium.

While shuttle catalysis has exploited a range of reversible chemical reactions, many enzyme-catalyzed processes are also freely reversible, and displacing the reaction equilibrium toward product formation is often achieved (both in Nature and synthetically) by performing cascade reactions where the product of one biocatalytic step becomes the substrate/reactant for the next transformation.^14−17^ Such cascade sequences enable the construction of complex molecules from relatively simple building blocks, and the compatibility of enzymes often means that multiple steps can be performed without the need for intermediate purification steps. The reversible nature of many enzyme transformations means that they can be exploited for mediating reactions in either the forward or reverse direction, and this adds a significant level of flexibility to the development of (chemo)enzymatic routes.^14,15^ An underexplored area of biocatalysis is using enzymes to shuttle functionality intra- or intermolecularly to generate reactive species *in situ*, which can undergo an irreversible, complexity-building, downstream step. We previously reported transaminase methodology that transferred amine functionality intramolecularly, generating a reactive aza-Michael precursor *in situ* and negating the need for an external amine donor (Figure 1b).^18^ The reversibility of the enzymatic reaction coupled with the spontaneous cyclization event ensured that only the thermodynamic product was isolated, rather than a potential mixture. Kroutil and co-workers reported the reversible shuttling of a methyl group using a cobalamin-dependent methyl transferase system.^19^

(Co)enzymes represent perfect examples of natural shuttle catalysts. For example, the nicotinamide coenzymes mediate reversible hydride transfer through Nature’s complex redox networks, and the concept of biocatalytic *hydrogen borrowing* has been derived from this natural shuttling methodology.^20−25^ The approach relies on the application of a redox self-sufficient single or coupled enzyme system, where the nicotinamide is recycled. Pyridoxal phosphate (PLP) is another example of a natural shuttling coenzyme that plays a vital mechanistic role in numerous PLP-dependent enzymes, including mediating the reversible transfer of amine functionality from a donor to an acceptor molecule in enzyme-catalyzed transamination reactions.^26−29^

Here, we present a new concept of biocatalytic *amine borrowing* via shuttle biocatalysis, using a transaminase to demonstrate the potential power of this methodology. While transaminases have been heavily exploited for the stereoselective installation of amine functionality, they are associated with the use of a sacrificial amine donor to install this functionality.^26,29^ In our approach, PLP functions to shuttle amine functionality from a donor to an acceptor, generating a reactive species *in situ*, which undergo a spontaneous downstream step to displace the reaction equilibrium in the forward direction (Figure 1c). This spontaneous downstream step serves to reunite the *borrowed* amine with the original amine donor to build molecular complexity. The introduction of molecular complexity is traditionally associated with linear syntheses, where intermediates are isolated and purified. However, new approaches that focus on building molecular complexity in an atom-efficient and sustainable manner are highly desirable, and our amine-borrowing methodology offers a new biocatalytic strategy for atom-efficient molecular construction. We demonstrate this amine-borrowing methodology using a biocatalytic equivalent of the Knorr-pyrrole synthesis and the Pictet–Spengler reaction to generate tetrahydroisoquinolines (THIQs).

The Knorr-pyrrole synthesis relies on the condensation of an α-amino ketone with a suitable carbonyl. Mechanistic investigations carried out by Xu et al. into the biocatalytic synthesis of pyrroles showed that an α-amino ketone and β-keto ester could be generated *in situ* using a commercially available transaminase.^30^ The Knorr-pyrrole synthesis provides an ideal model reaction to showcase our amine-borrowing methodology (Scheme 1). The approach centers on the one-pot conversion of a β-amino ester (**1**) and α-diketone (**2**) to the corresponding β-ketoester (**3**) and α-aminoketone (**4**), respectively. In this approach, PLP shuttles the amine functionality from substrate **1** to **2**, generating products **3** and **4**, which were expected to undergo a spontaneous Knorr-pyrrole reaction. Generating the α-aminoketone *in situ* is advantageous, as these compounds are unstable to oxidative dimerization and can readily form pyrazines.^30,31^ Our ideal amine borrowing conditions should employ stoichiometric equivalents of donor and acceptor, minimize byproduct generation, and afford the target pyrrole products in high conversion/yield (Figure 1c).

**Scheme 1:**
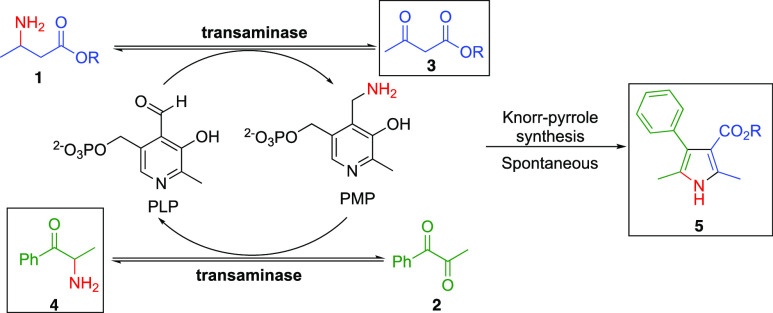
Knorr-Pyrrole Reaction Demonstrating the Concept of Biocatalytic Amine Borrowing The transaminase products that condense to form **5** are shown in the box.

Commercially available (*R*)-selective ATA117 was chosen as a biocatalyst, as it has previously been shown to accept substrates **1** and **2**. Initial efforts focused on optimizing the enzyme loading, substrate concentration and pH for the reaction of racemic β-amino ester **1a** with α-diketone **2a** (Table 1; see SI (Table S1) for more details). Despite the use of 2 equiv of the racemic donor, only the (*R*)-enantiomer (therefore 1 equiv) is readily available to the enzyme. A series of optimization studies (Table S1) suggested that an enzyme loading of 5 mg mL^–1^ at pH 9 and 40 mM substrate concentration enabled efficient conversion of **1a** and **2a** to pyrrole **5a** via the intermediate generation of reactive species **3a** and **4a** (Table 1, entry 4). These optimized conditions were used for all subsequent biotransformations.

**Table 1:**
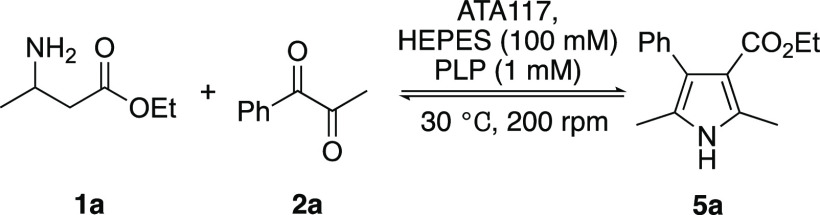
Optimizing Amine-Borrowing Conditions Using Model Substrates *rac*-**1a** and **2a**^a^

entry	conc of **2a** (mM)	pH	conv (%) (24 h)
1	5	8	68
2	5	9	77
3	20	9	86
4	40	9	94

a Reaction conditions: 1-phenylpropane-1,2-dione **2a**, ethyl 3-aminobutanoate **1a** (2 racemic equiv; 1 equiv of *R*-**1a**), ATA117 (5 mg/mL^–1^), HEPES (100 mM, 0.5 mL), DMSO (10% v/v), 30 °C, 200 rpm. Conversion was measured by HPLC. Results are the mean of three replicates. Conversions were comparable after 48 h.

Diketone substrates **2a**–**f** (Table 2) were selected to encompass a broad range of electronic variations on the aromatic ring, including weakly (**2b**) to strongly (**2d**) electron-withdrawing groups and electron-donating groups (**2e**). Diketone **2f** was chosen to explore the efficiency of the cascade starting from dialkyl α-diketones, as α-amino ketones are significantly more susceptible to oxidative dimerization to form pyrazines, compared to their aryl-functionalized counterparts.

**Table 2:**
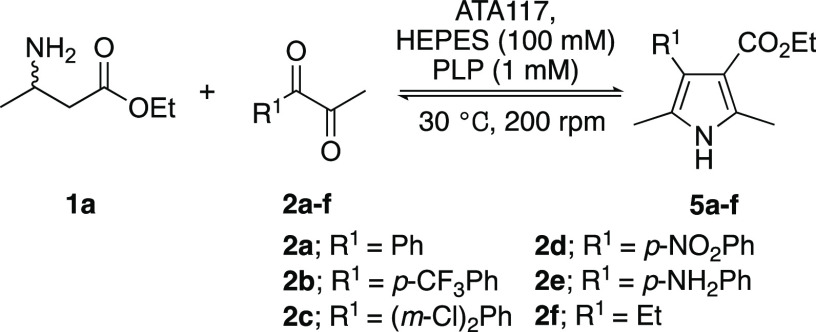
Preparative-Scale Reactions between Racemic β-Amino Ethyl Ester **1a** and a Range of Diketones **2a**–**f**^a^

entry	product	R^1^	β-amino ester (equiv)	conv (%) (72 h)^b^	yield (%)
1	**a**	Ph	2^e^	90	52^c^
2	**b**	*p*-CF_3_Ph	2^e^	99	64^c^
3	**c**	(*m*-Cl)_2_Ph	2^e^	78	46^c^
4	**d**	*p*-NO_2_Ph	2^e^	41	25^c^
5	**e**	*p*-NH_2_Ph	2^e^	83	54^d^
6	**f**	Et	2^e^	34	28^c^

a Reaction conditions: (*R*/*S*)-ethyl 3-aminobutanoate (*R*/*S*)-**1a** (80 mM), diketone **2a**–**f** (40 mM), HEPES (100 mM, pH 9), ATA117 (5 mg/mL^–1^), DMSO (10% v/v), 30 °C, 200 rpm, final volume of 10 mL.

b Conversion measured by HPLC.

c Isolated yield after column chromatography.

d Isolated yield after preparative HPLC.

e Racemic ethyl 3-aminobutanoate, where only 1 equiv is available to the enzyme.

ATA117 showed good conversion (41–99%) across the aromatic substrates tested, affording the corresponding pyrrole products **5a**–**e** (Table 2, entries 1–5). There was no obvious correlation with the ring electronics and the conversion to the Knorr-pyrrole products. The poor conversion with alkyl diketone **1f** was anticipated using stoichiometric concentrations of the coupling partners, due to the likely formation of the pyrazine homodimer product;^30,31^ however, this potential side product was never observed. It is worth noting that, while we did not try this approach, application of both an (*R*)- and (*S*)-selective TA concurrently would enable both enantiomers of **1a** to be converted to **3a**.

Having achieved good conversions with racemic ethyl 3-aminobutanoate **1a**, we next switched to just 1 equiv of enantiopure (*R*)-methyl 3-aminobutanoate (*R*)-**1b** (Table S2). The switch to the methyl derivative **1b** was solely based on the commercial availability of this molecule in its enantiomerically pure form. In most cases, the conversions were broadly comparable to those observed using the racemic ethyl derivative. The ATA Knorr-pyrrole cascade with **1b** and *m*-Cl diketone derivative **2c** (Scheme 2, Table S2, entry 3) elegantly demonstrates the potential efficiency of the amine-borrowing methodology. The PLP functions to transfer the amino group from the β-amino ester donor **1b** to the α-diketone acceptor **2c**, generating reactive species **3b** and **4c**. The spontaneous Knorr-pyrrole condensation functions to effectively displace the reaction equilibrium using stoichiometric equivalents (40 mM) of donor and acceptor, resulting in 95% conversion (80% yield) to pyrrole **5i**.

**Scheme 2:**
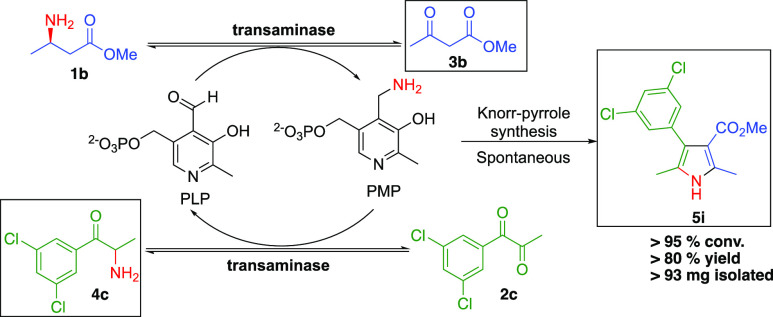
Preparative-Scale Transaminase-Mediated Amine-Borrowing Reaction for the Synthesis of **5i**, Reaction conditions: (*R*)-methyl 3-aminobutanoate **(***R***)-1b** (40 mM), diketone **2c** (40 mM), HEPES (100 mM, pH 9), ATA117 (5 mg mL^–1^), DMSO (10% v/v), 30 °C, 200 rpm, final volume of 10 mL. Transaminase products that condense to form **5i** are shown in the box.

Next, we explored amine borrowing for the preparation of THIQs, using the Pictet–Spengler (PS) rection. This reaction takes place between a β-arylethylamine and a suitable carbonyl, often an aldehyde.^32^ We rationalized that it may be desirable to generate the carbonyl *in situ*, as many aldehydes are unstable. Additionally, unlike the Knorr-pyrrole example discussed above, the C–C bond-forming PS cascade will lead to the installation of a new chiral center, starting from achrial starting materials. We envisaged using an ATA to generative reactive intermediates **7** and **9***in situ* by transamination of ketone **8** in the presence of amine **6**, followed by a subsequent PS reaction to afford the target THIQs (Scheme 3). Previous studies have demonstrated the mechanistic importance of β-arylethylamines bearing an electron-donating group in the *meta* position,^33,34^ as they possess increased electron density at the point of ring closure, facilitating nucleophilic attack of the imine.

**Scheme 3:**
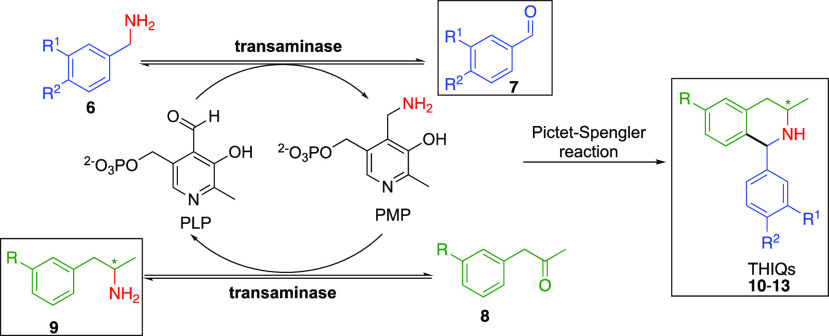
ATA/Pictet–Spengler Cascade Demonstrating the Concept of Biocatalytic Amine Borrowing for the Synthesis of THIQs from Achiral Substrates The transaminase products that condense to form **10**–**13** are shown in the box.

Prior to exploring the complete cascade, we first sought to ensure that the PS reaction was feasible under conditions compatible with the ATAs. To explore this, β-arylethylamine **9a**, the product of the transamination of the corresponding ketone **8**, was synthesized and exposed to benzaldehyde **7a** in the presence of KP_i_ buffer (Figure 2A). The groups of Hailes and Ward have demonstrated that phosphate (P_i_) can catalyze the PS reaction^34,35^ and it is also tolerated by many ATAs; therefore, our focus was on optimizing the pH and cosolvent (see Figure S1 for full details). Our optimization studies revealed that pH 6 or 7 in DMSO gave poor to moderate conversion, and therefore, we initially evaluated a range of solvents and identified MeOH as the most suitable cosolvent for the reaction. The PS reaction between **7a** and **9a** proceeded efficiently at pH 6 or 7 when using 10–50% MeOH as the cosolvent. (Figure S1). At higher pH values, there was increased conversion to the Schiff base, as observed by GC–MS, but subsequent ring closure did not readily occur (data not shown). In all cases, a dr of 2:3 was observed. Having established reaction conditions compatible with the ATAs, ketone **8a** was reacted with benzylamine **6a** in the presence of (*R*)-selective ATA025 and (*S*)-selective ATA256 (Figure 2B). However, conversion to the THIQ product was extremely low (<10%), and NMR analysis of the reaction mixtures suggested that the enzyme was not effectively converting ketone **8a** to the corresponding amine. This is likely due to incompatibility between the low pH necessary for effective PS reaction and the typically high pHs used for ATA reactions (pH 8–10). An alternative amine donor, vanillamine **6b**, was selected and screened with a small panel of ketones **8a**-**c**, using DMSO as the cosolvent, as this solvent was found to be optimum for the PS reaction with this amine donor.

**Figure 2:**
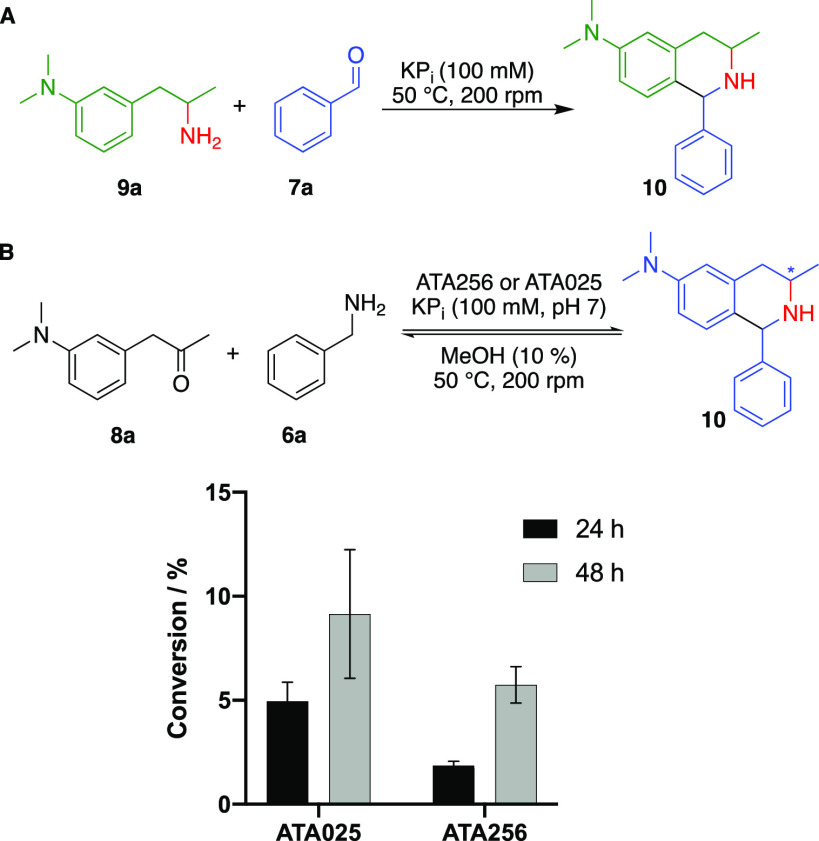
(A) Optimizing the Pictet–Spengler reaction conditions for the condensation of racemic **9a** and **7a** (see Figure S1 for details). (B) ATA/Pictet–Spengler cascade for the synthesis of THIQ **10** using ATA025 and ATA256. The conversion with each enzyme is shown in the graph.

A small selection of the data gathered is detailed in Table 3 (see Table S3 for more details). As expected, the reaction with ketone **8a**, bearing a NMe_2_ substituent, showed very low conversion to THIQ **11** (entry 1). *m*-Methoxy-substituted ketone **8b** was converted to the corresponding amine **9b** (32%), but there was no THIQ **12** product detected. The cascade involving ketone **8c** with a *m*-hydroxy substituent showed moderate, but significant, conversion to THIQ **13**. At 40 mM ketone concentration, the conversion increased slightly as the equivalents of vanillamine were increased (entries 3–5). In all cases, analysis of the reaction mixtures showed that significant quantities of amine **9c** were formed and not condensing further to the THIQ (entries 3–6). Relatively more THIQ formed at higher concentrations (entry 6) due to the biomolecular nature of the reaction, but overall conversion began to drop. We also observed likely degradation of amine **9c**, particularly at lower concentrations (see SI Table S3), and it is likely that this degradation is contributing to the low conversions to the THIQ products. *p*-NO_2_-benzylamine was also investigated as an amine donor, as it was thought that the electron-withdrawing nitro group would aid the PS reaction. However, the only product observed was that of the condensation between the *p*-NO_2_-benzaldehyde and *p*-NO_2_-benzylamine. While the *ee* of the transformation has not yet been explored, it is likely that the ATA step is highly selective but that the subsequent PS reaction is not selective. It is worth noting that we also explored the PS cascade with a small panel of aldehydes, bearing a *m*-OH group but only saw trace quantities of THIQ formed (see the SI Appendix for details). Despite the moderate conversion observed with these PS cascades, we believe these reactions represent interesting early examples of applying amine borrowing to initiate C–C bond formation and install new chiral functionality.

**Table 3:**
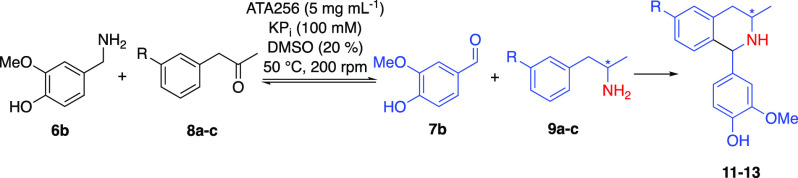
Optimizing Amine-Borrowing Conditions for the ATA/Pictet–Spengler Cascade, Starting from Vanillamine **6b** and Ketones **8a**–**c**^a^

entry	R	**8** (mM)	amine (equiv)	conv to **9** (%)	conv to THIQ (%)	THIQ product
1	NMe_2_	100	1.1	3	3	**11**
2	OMe	50	1.1	32	0	**12**
3	OH	40	1.1	14	17	**13**
4	OH	40	2	21	27	**13**
5	OH	40	5	27	31	**13**
6	OH	100	1.1	11	28^b^	**13**

a Reaction conditions: vanillamine **6b** (1.1–5 equiv), phenylacetone derivative **8a**-**c** (40–100 mM), KP_i_ (100 mM, pH 7.5), ATA256 (5 mg mL^–1^), DMSO (20% v/v), 50 °C, 200 rpm, final volume of 1 mL. Conversion measured by NMR and the results represent the mean of three replicates.

b DMSO concentration was 10%. A 2:3 *dr* was observed in each case.

In conclusion, we introduce the new concept of amine borrowing *via* shuttle biocatalysis and showcase our methodology using the Knorr-pyrrole synthesis and the Pictet–Spengler reaction. Our approach centers on the transaminase-mediated *in situ* generation of reactive intermediates, which subsequently condense to generate the pyrrole or THIQ products. In contrast to the majority of other transaminase reactions,^26,29^ our methodology does not rely on an external amine donor but harnesses the shuttling capabilities of PLP to *borrow* amine functionality from a donor, which is ultimately returned following a spontaneous condensation. This represents a positive step toward developing biocatalytic processes with high atom economy. In our best example, we show that stoichiometric equivalents of reactive precursors (transaminase substrates) can be almost quantitatively converted to the pyrrole product (95%). This methodology has the potential to be extended to alternative group transfer reactions and to a range of biocatalytic systems, and efforts are ongoing in this regard. The concept of employing enzymes that typically mediate functional group transfer reactions to generate reactive species, which can undergo a complexity building downstream step, has the potential to complement the ever-growing range of biocatalytic methodology and expand the range of transformations achievable via shuttle (bio)catalysis.

## Supporting Information Available

Experimental procedures and data (PDF)

## Dedication

This manuscript is dedicated to the memory of Pat O’Reilly (March 1944–October 2021).

## References

[ref1] Bhawal B. N.; Morandi B. Shuttle Catalysis - New Strategies in organic Synthesis. Chem. - Eur. J. 2017, 23, 12004–12012. 10.1002/chem.201605325. 28125163

[ref2] Bhawal B. N.; Morandi B. Catalytic Transfer Functionalization through Shuttle Catalysis. ACS Catal. 2016, 6, 7528–7535. 10.1021/acscatal.6b02333.

[ref3] Bhawal B. B.; Morandi B. Catalytic Isofunctional Reactions – Expanding the Repertoire of Shuttle and Metathesis Reactions. Angew. Chem., Int. Ed. 2019, 58, 10074–10103. 10.1002/anie.201803797. 30192427

[ref4] Edwards M. G.; Williams J. M. J. Catalytic Activation: Indirect “Wittig” Reaction of Alcohols. Angew. Chem., Int. Ed. 2002, 41, 4740–4743. 10.1002/anie.200290034. 12481344

[ref5] Guillena G.; Ramón D. J.; Yus M. Alcohols as Electrophiles in C-C Bond-forming Reactions: The Hydrogen Autotransfer Process. Angew. Chem., Int. Ed. 2007, 46, 2358–2364. 10.1002/anie.200603794. 17465397

[ref6] Hamid M. H. S. A.; Slatford P. A.; Williams J. M. J. Borrowing Hydrogen in the Activation of Alcohols. Adv. Synth. Catal. 2007, 349, 1555–1575. 10.1002/adsc.200600638.

[ref7] Reed-Berendt B. G.; Latham D. E.; Dambatta M. B.; Morrill L. C. Borrowing Hydrogen for Organic Synthesis. ACS Cent. Sci. 2021, 7, 570–585. 10.1021/acscentsci.1c00125. 34056087 PMC8155478

[ref8] Corma A.; Navas J.; Sabater M. J. Advances in One-Pot Synthesis through Borrowing Hydrogen Catalysis. Chem. Rev. 2018, 118, 1410–1459. 10.1021/acs.chemrev.7b00340. 29319294

[ref9] Fang X.; Cacherat B.; Morandi B. CO- and HCl-free synthesis of acid chlorides from unsaturated hydrocarbons via shuttle catalysis. Nat. Chem. 2017, 9, 1105–1109. 10.1038/nchem.2798. 29064496

[ref10] Yu P.; Bismuto A.; Morandi B. Iridium-Catalyzed Hydrochlorination and Hydrobromination of Alknes by Shuttle Catalysis. Angew. Chem., Int. Ed. 2020, 59, 2904–2910. 10.1002/anie.201912803. PMC7028031 31769578

[ref11] Fang X.; Yu P.; Morandi B. Catalytic reversible alkene-nitrile interconversion through controllable transfer hydrocyanation. Science 2016, 351, 832–836. 10.1126/science.aae0427. 26912891

[ref12] Murphy S. K.; Park J.-W.; Cruz F. A.; Dong V. M. Rh-catalyzed C-C bond cleavage by transfer hydroformylation. Science 2015, 347, 56–60. 10.1126/science.1261232. 25554782 PMC4445961

[ref13] Boehm P.; Morandi B. New Catalysis Concepts for Molecular Design and Feedstocks Valorization. Chimia 2020, 74, 724–729. 10.2533/chimia.2020.724. 32958111

[ref14] Huffman M. A.; Fryszkowska A.; Alvizo I.; Borra-Garske M.; Campos K. R.; Canada K. A.; Devine P. N.; Duan D.; Forstater J. H.; Grosser S. T. Design of an in vitro biocatalytic cascade for the manufacture of islatravir. Science 2019, 366, 1255–1259. 10.1126/science.aay8484. 31806816

[ref15] O’Reilly E.; Ryan J. Biocatalytic cascades go viral. Science 2019, 366, 1199–1200. 10.1126/science.aaz7376. 31806802

[ref16] France S. P.; Hepworth L. J.; Turner N. J.; Flitsch S. L. Constructing Biocatalytic Cascades: In Vitro and in Vivo Approaches to de Novo Multi-Enzyme Pathways. ACS Catal. 2017, 7, 710–724. 10.1021/acscatal.6b02979.

[ref17] Kuska J.; O’Reilly E. Engineering biosynthetic pathways and biocatalytic cascades for sustainable synthesis. Curr. Opin. Chem. Biol. 2020, 58, 146–154. 10.1016/j.cbpa.2020.08.006. 33152607

[ref18] Ryan J.; Šaiučiulis; Gomm A.; Maciá B.; O’Reilly E.; Caprio V. Transaminase Triggered Aza-Michael Approach for the Enantioselective Synthesis of Piperidine Scaffolds. J. Am. Chem. Soc. 2016, 138, 15798–15800. 10.1021/jacs.6b07024. 27960354

[ref19] Farnberger J. E.; Richter N.; Hiebler K.; Bierbaumer S.; Pickl M.; Skibar W.; Zepeck F.; Kroutil W. Biocatalytic methylation and demethylation via a shuttle catalysis concept involving corrinoid proteins. Commun. Chem. 2018, 1, 1–8. 10.1038/s42004-018-0083-2.

[ref20] Wandrey C.; Fiolitakis E.; Wichmann U. L-Amino Acids from a Racemic Mixture of α-Hydroxy Acids. Ann. N. Y. Acad. Sci. 1984, 434, 91–94. 10.1111/j.1749-6632.1984.tb29805.x. 6596905

[ref21] Willetts A. J.; Knowles C. J.; Levitt M. S.; Roberts S. M.; Sandey H.; Shipston N. F. Biotransformation of *endo*-Bicyclo[2.2.1]heptan-2-ols and *endo*-Bicyclo[3.2.0]-hept-2-en-6-ol into the Corresponding Lactones. J. Chem. Soc., Perkin Trans. 1 1991, 1608–1610. 10.1039/p19910001608.

[ref22] Mutti F. G.; Knaus T.; Scrutton N. S.; Breuer M.; Turner N. J. Conversion of alcohols to enantiopure amines through dual-enzyme hydrogen-borrowing cascades. Science 2015, 349, 1525–1529. 10.1126/science.aac9283. 26404833 PMC4883652

[ref23] Montgomery S. L.; Mangas-Sanchez J.; Thompson M. P.; Aleku G. A.; Dominguez B.; Turner N. J. Direct Alkylation of Amines with Primary and Secondary Alcohols through Biocatalytic Hydrogen Borrowing. Angew. Chem., Int. Ed. 2017, 56, 10491–10494. 10.1002/anie.201705848. 28671344

[ref24] Knaus T.; Mutti F. G. Biocatalytic hydrogen-borrowing cascades. Chim. Oggi. 2017, 35, 34–37. PMC5837015 29515288

[ref25] Ramsden J. I.; Heath R. S.; Derrington S. R.; Montgomery S. L.; Mangas-Sanchez J.; Mulholland K. R.; Turner N. J. Biocatalytic *N*-Alkylation of Amines Using Either Primary Alcohols or Carboxylic Acids via Reductive Aminase Cascades. J. Am. Chem. Soc. 2019, 141, 1201–1206. 10.1021/jacs.8b11561. 30601002

[ref26] Gomm A.; O’Reilly E. Transaminases for chiral amine synthesis. Curr. Opin. Chem. Biol. 2018, 43, 106–112. 10.1016/j.cbpa.2017.12.007. 29278779

[ref27] Eliot A. C.; Kirsch J. F. Pyridoxal Phosphate Enzymes: Mechanistic, Structural, and Evolutionary Considerations. Annu. Rev. Biochem. 2004, 73, 383–415. 10.1146/annurev.biochem.73.011303.074021. 15189147

[ref28] Liang J.; Han Q.; Tan Y.; Ding H.; Li J. Current Advances on Structure-Function Relationships of Pyridoxal 5′-Phosphate-Dependent Enzymes. Front. Mol. Biosci. 2019, 6, 1–21. 10.3389/fmolb.2019.00004. 30891451 PMC6411801

[ref29] Savile C. K.; Janey J. M.; Mundorff E. C.; Moore J. C.; Tam S.; Jarvis W. R.; Colbeck J. C.; Krebber A.; Fleitz F. J.; Brands J.; Devine P. N.; Huisman G. W.; Hughes G. J. Biocatalytic asymmetric synthesis of chiral amines from ketones applied to sitagliptin manufacture. Science 2010, 329, 305–309. 10.1126/science.1188934. 20558668

[ref30] Xu J.; Green A. P.; Turner N. J. Chemo-Enzymatic Synthesis of Pyrazines and Pyrroles. Angew. Chem., Int. Ed. 2018, 57, 16760–16763. 10.1002/anie.201810555. PMC6391939 30335228

[ref31] Mortzfeld F. B.; Hashem C.; Vranková K.; Winkler M.; Rudroff F. Pyrazines: Synthesis and Industrial Application of these Valuable Flavor and Fragrance Compounds. Biotechnol. J. 2020, 15, 1–7. 10.1002/biot.202000064.

[ref32] Stöckigt J.; Antonchick A. P.; Wu F.; Waldmann H. The Pictet-Spengler Reaction in Nature and in Organic Chemistry. Angew. Chem., Int. Ed. 2011, 50, 8538–8564. 10.1002/anie.201008071. 21830283

[ref33] Quevedo R.; Baquero E.; Rodriguez M. Regioselectivity in isoquinoline alkaloid synthesis. Tetrahedron Lett. 2010, 51, 1774–1778. 10.1016/j.tetlet.2010.01.115.

[ref34] Pesnot T.; Gershater M. C.; Ward J. M.; Hailes H. C. Phosphate mediated biomimetic synthesis of tetrahydroisoquinoline alkaloids. Chem. Commun. 2011, 47, 3242–3244. 10.1039/c0cc05282e. 21270984

[ref35] Zhao J.; Méndez-Sánchez D.; Ward J. M.; Hailes H. C. Biomimetic Phosphate-Catalyzed Pictet-Spengler reaction for the Synthesis of 1,1’-Disubstituted and Spiro-Tetrahydroisoquinoline Alkaloids. J. Org. Chem. 2019, 84, 7702–7710. 10.1021/acs.joc.9b00527. 31095375 PMC7007230

