# Supplementary material for: Transaminase-Mediated Amine Borrowing via Shuttle Biocatalysis
Source: Org Lett. 2021 Dec 15;24(1):74–9. doi: 10.1021/acs.orglett.1c03320 (PMC8762705; doi:10.1021/acs.orglett.1c03320)
Supplement: Supplementary file 1 — ol1c03320_si_001.pdf [file ol1c03320_si_001.pdf]

# Supporting Information for

## Transaminase-Mediated *Amine Borrowing via* Shuttle Biocatalysis

Freya Taday,[b] James Ryan,[a] Rachel O'Sullivan[a] and Elaine O'Reilly[a]*

[a]School of Chemistry, University College Dublin, Science Centre South, Belfield, Dublin 4, Ireland.
[b]School of Chemistry, University of Nottingham, University Park, Nottingham, NG7 2RD, UK.

**Email**
elaine.oreilly@ucd.ie

## Table of Contents

**General Methods and Materials** **2**

**Procedure for the synthesis of diones** **3**

**Procedure for the synthesis of ketones 8a and 8c** **3**

**Biotransformations for ATA-Knorr Pyrrole cascade reaction** **8**
General Procedure for Optimization Studies: Analytical-Scale Biotransformations for the Preparation of Pyrrole (**5a**)[4] 8
Table S1. Optimization of biocatalytic Knorr-pyrrole synthesis 8
General Procedure for Preparative-Scale Biotransformations: Preparation of Pyrroles (**5a-l**)[4] 9
Representative example of a preparative-scale procedure

**Characterisation of pyrroles (5a-f)** **10**

**Procedure for analytical scale Pictet-Spengler reactions** **14**

**Analytical scale synthesis of THIQ 11-13 *via* TA/Pictet-Spengler cascade** **14**
Table S2. Optimisation of biocatalytic Pictet-Spengler reaction 14
Figure S1. Optimizing Pictet-Spengler reaction conditions for the condensation of racemic **9a** and **7a**. 14
Table S3. Concentration screen for substrates **8c** and **6b** 14
Figure S1. Optimizing the Pictet-Spengler reaction conditions for the condensation of racemic **9a** and **7a**. 15
Table S4. Concentration screen for substrates **8c** and **6b**. 16

**Calculation used to work out the conversion of biotransformations *via* quantitative NMR analysis** **17**

**$^1$H NMR and $^{13}$C NMR Spectra** **18**

**Appendix** **39**
Procedure for analytical scale Pictet-Spengler reactions with ß-phenylethylamines 40
Analytical scale biotransformations for the synthesis of THIQ (**S18**) from aldehydes **S16a** and **S16b** 40
Procedure for the synthesis of aldehydes for the Pictet-Spengler reaction 41
$^1$H and $^{13}$C NMR 43

**References** **45**

## General Methods and Materials

**Methods**: NMR spectra were recorded on a Bruker DPX 400, a Bruker AV(III)500 spectrometer or a Varian VnmrS (400 MHz for $^{1}H$ and 100 MHz for $^{13}C$). The chemical shift values (δ) are reported in ppm with the residual solvent referenced to $CDCl_3$: δ 7.26 for $^{1}H$-NMR, δ 77.0 for$^{13}C$ NMR; or MeOD: δ 3.31 for $^{1}H$-NMR, δ 49.0 for $^{13}C$ NMR. Coupling constants (*J*) are reported in Hz and refer to the observed peak multiplicities. Where needed, 2D NMR was preformed to assign specific carbon atoms and determine stereochemistry. Analytical HPLC was performed on a Thermo Ultimate 3000 uHPLC system equipped with PDA el detector (l = 210 – 400 nm). The mobile phase composed of 0.1% trifluoroacetic acid in $H_2O$ (Solvent A) and 0.1% trifluoroacetic acid in acetonitrile (Solvent B). The analysis of the chromatograms was conducted using Chromeleon 7 software. Mass spectra were recorded on a Bruker MicroTOF II or an Agilent 6546 Quadrupole Time-Of-Flight MS System spectrometer using Electron Spray Ionization (ESI). IR spectra were recorded on a Bruker ATR or solution cell. GC-FID analysis was performed on a Bruker Trace 1310 series GC equipped with an autosampler and a Chirasil Dex CB (25 m x 0.25 mm x 0.25 mm) column following **gradient 1**: oven was held at 40 °C for 2 min, before the temperature was increased to 150 °C at a rate of 20 °C $min^{-1}$ and held for 5 min, the temperature was then increased to 200 °C at a rate of 30 °C $min^{-1}$ and held for 18 min; or on an Aligent 8860 GC System equipped with an autosampler and a HP-5 column (30 m x 0.32 mm x 0.25 μm) following **gradient 2**: the oven was held at 40 °C for 2 min, before the temperature was increased to 150 °C at a rate of 20 °C $min^{-1}$ and held for 5 min, the temperature was then increased to 270 °C at a rate of 30 °C $min^{-1}$ and held for 18 min. Preparative reverse-phase HPLC was performed using a Waters 1525 binary pump HPLC equipped with a dual wavelength UV detector set to 210 nm and 280 nm. Preparative HPLC was performed on a Waters Sunfire 5 μm (C-18) preparative column with 5-μm particle size, 19 x 150 mm, operating at a flow rate of 6 mL min-1 using a mobile phase of 0.1% trifluoroacetic acid in water (Solvent A) and 0.1% trifluoroacetic acid in acetonitrile (Solvent B) using the gradient specified in the experimental section. Unless otherwise stated, heated reactions were performed on a hotplate equipped with a Drysyn® and temperature probe.

**Materials:** Commercially available reagents and solvents were purchased from Acros Chemicals, Fluorochem, Sigma Aldrich and Thermo Fisher Scientific. 1-(4-nitrophenyl)propane-1,2-dione **2d**, sourced from Fluorochem, was purified by column chromatography before use, while all other commercial chemicals were used without further purification. Anhydrous $CH_2Cl_2$ was obtained from a Pure Solvent apparatus. Thin layer chromatography was performed on Alfa Aesar silica gel 60 F254 plates. Flash column chromatography was performed on silica gel (60 Å, 230-400 mesh). Commercially available transaminases, ATA117, ATA025 and ATA256, were purchased from Codexis® in the form of lyophilized cell extract.

## Procedure for the synthesis of diones

### 1-(4-Aminophenyl)propane-1,2-dione (2e)

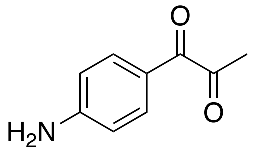

To a solution of 1-(4-nitrophenyl)propane-1,2-dione (1.976 g, 10.23 mmol) in $H_2O$:EtOH (34 mL(8:5)) was added iron powder (1.713 g, 30.96 mmol), and ammonium chloride (2.736 g, 51.15 mmol). The reaction mixture was heated under reflux for 1 h and monitored by TLC ($Et_2O$). Upon completion, celite was added to form a slurry and the mixture was filtered through a pad of celite and washed with dichloromethane (40 mL). The aqueous layer was separated and washed with dichloromethane (40 mL x2), the combined organic layers were washed with brine (50 mL) and dried with anhydrous magnesium sulfate, filtered and concentrated *in vacuo*. The crude product was purified by column chromatography ($Et_2O$) to give the title compound as a brown oil (872 mg, 52 %). $^1$H NMR (400 MHz, $CDCl_3$) $\delta_H$ 7.85 (2H, d, $J$ = 8.7 Hz, ArC**H**), 6.64 (2H, d, $J$ = 8.7 Hz, ArC**H**), 4.33 (2H, s, N**H**$_2$), 2.47 (3H, s, C**H**$_3$); $^{13}$C NMR (100 MHz, $CDCl_3$) $\delta_C$ 202.2 (**C**=O), 190.0 (**C**=O), 152.8 (Ar**C**$_q$), 133.2 (ArCH), 121.8 (Ar**C**$_q$), 114.1 (Ar**C**H), 26.8 (**C**H$_3$); FTIR (ATR) $\upsilon_{max}$: 3467, 3363, 3233, 1703, 1578, 1320, 1151 cm$^{-1}$; HRMS (ESI) m/z: $[M + H]^+$ Calcd for 164.0706 for $C_9H_{10}NO_2^+$; Found 164.0704.

## Procedure for the synthesis of ketones 8a and 8c

### 2-(3-nitrophenyl)acetic acid (I-1)

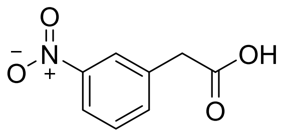

To a solution of concentrated sulfuric acid (8 mL), glacial acetic acid (8 mL), in water (8 mL) was added 2-(3-nitrophenyl)acetonitrile (5 g, 30.8 mmol) the reaction mixture was heated under reflux for 1 h. The reaction mixture was cooled and diluted with water (20 mL) washed with EtOAc (30 mL x3). The combined organic layers were washed with water (40 mL x3), brine (40 mL), dried with anhydrous magnesium sulfate and filtered. The resultant residue was concentrated *in vacuo* to give the title compound as a white solid with no further purification (5.393 g, 29.7 mmol, 97 %). m.p 102 – 105 °C; $^1$H NMR (400 MHz, $CDCl_3$) $\delta_H$ 8.20 - 8.13 (2H, m, Ar**H**), 7.68 - 7.60 (1H, m, Ar**H**), 7.57 - 7.48 (1H, m, Ar**H**), 3.79 (2H, s, C**H**$_2$); $^{13}$C NMR (100 MHz, CDCl3) $\delta_C$ 176.2 (**C**=O), 148.5 (Ar**C**$_q$), 135.8 (Ar**C**H), 135.1 (Ar**C**$_q$), 129.7 (Ar**C**H), 124.7 (Ar**C**H), 122.7 (Ar**C**H), 40.4 (**C**H$_2$); FTIR (ATR) $\upsilon_{max}$: 3068, 2927, 1703, 1523, 1347, 713 cm$^{-1}$; HRMS (ESI) m/z $[M+H]^+$ Calcd for $C_8H_8NO_4^+$ 182.0448; Found 182.0450. In accordance to literature data. [1]

**N-methoxy-N-methyl-2-(3-nitrophenyl)acetamide (I-2)**

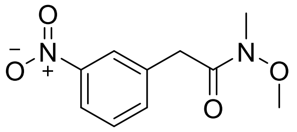

2-(3-nitrophenyl)acetic acid **I-1** (5.390 g, 29.7 mmol) and 1,1'-carbonyldiimidazole (4.825 g, 29.8 mmol) were dissolved in dichloromethane (100 mL) and mixed for 1 h before N,O-dimethylhydroxylamine (3.61 g, 59.5 mmol) was added. The reaction mixture was stirred for 24 h. Once the reaction was completed it was diluted in water (50 mL), the organic layer was separated, and the aqueous layer was washed with dichloromethane (50 mL x2). The combined organic layers were dried with anhydrous magnesium sulfate, filtered and concentrated *in vacuo* to give N-methoxy-N-methyl-2-(3-nitrophenyl)acetamide as a white solid (5.638 g, 24.7 mmol, 84 %) without further purification. m.p 90 - 92 °C; $^1$H NMR (400 MHz, $CDCl_3$) $\delta_H$ 8.17 – 8.08 (2H, m, ArC**H**), 7.68 – 7.61 (1H, m, ArC**H**), 7.49 (1H, t, *J* = 7.9 Hz, ArC**H**), 3.87 (2H, s, C**H**$_2$), 3.71 (3H, s, OC**H$_3$**), 3.21 (3H, s, C**H$_3$**); $^{13}$C NMR (100 MHz, $CDCl_3$) $\delta_C$ 207.1 (**C**=O), 148.4 (Ar**C**$_q$), 136.9 (Ar**C**$_q$), 136.0 (Ar**C**H), 129.5 (Ar**C**H), 124.7 (Ar**C**H), 122.2 (Ar**C**H), 61.6 (O**C**H$_3$), 38.8 (**C**H$_2$), 31.1 (**C**H$_3$); FTIR (ATR) $\upsilon_{max}$: 3038, 2920, 1654, 1518, 1342 cm$^{-1}$; HRMS (ESI) m/z: $[M+H]^+$ Calcd for $C_{10}H_{13}N_2O_4^+$ 225.0870; Found 225.0873.

**2-(3-aminophenyl)-N-methoxy-N-methylacetamide (I-3)**

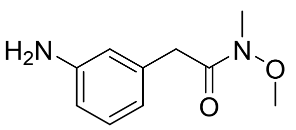

To a solution of *N*-methoxy-N-methyl-2-(3-nitrophenyl)acetamide **I-2** (5.60 g, 24.9 mmol) in $H_2O$:EtOH (39 mL (8:5)) was added iron powder (4.18 g, 74.9 mmol), and ammonium chloride (6.670 g, 124 mmol). The reaction mixture was heated under reflux for 1 h. Once the reaction was completed celite was added to from a slurry and the mixture was filtered through a pad of celite and washed with dichloromethane (40 mL). The aqueous layer was separated and washed with dichloromethane (40 mL x2), the combined organic layers were washed with brine (40 mL) and dried with anhydrous magnesium sulfate, filtered and concentrated *in vacuo*. The crude product was purified by column chromatography ($Et_2O$) to give the title compound as a brown oil (3.242, 16.7 mmol, 67 %). $^1$H NMR (400 MHz, $CDCl_3$) $\delta_H$ 7.09 (1H, t, *J* = 8.0 Hz, ArC**H**), 6.73-6.66 (2H, m, ArC**H**), 6.61 – 6.55 (1H, m, ArC**H**), 3.68 (3H, s, C**H**$_2$), 3.60 (3H, s, OC**H**$_3$), 3.19 (3H, s, C**H**$_3$); $^{13}$C NMR (100 MHz, $CDCl_3$) $\delta_C$ 172.6 (**C**=O), 146.3 (Ar**C**$_q$), 136.2 (Ar**C**$_q$), 129.6 (Ar**C**H), 120.0 (Ar**C**H) 116.2 (Ar**C**H), 114.0 (Ar**C**H), 61.5 (O**C**H$_3$), 39.5 (**C**H$_2$), 32.4 (**C**H$_3$); FTIR (ATR) $\upsilon_{max}$: 3437, 3351, 2936, 1646, 1602, 1406, 994 cm$^{-1}$; HRMS (ESI) m/z: $[M+H]^+$ Calcd. for $C_{10}H_{15}N_2O_2^+$ 195.1128; Found 195.1127. In accordance to literature data. [2]

**2-(3-(dimethylamino)phenyl)-N-methoxy-N-methylacetamide (I-4)**

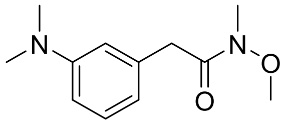

To 2-(3-aminophenyl)-N-methoxy-N-methylacetamide **I-3** (3.200 g, 16.4 mmol), tetrabutylammonium iodide (426 mg, 1.15 mmol) and potassium hydroxide (2.300 g, 41.2 mmol) dissolved in toluene:water (24 mL (7:1)), iodomethane (2.56 mL, 41.2 mmol) was added dropwise. The reaction mixture was heated at 90 °C for 24 h. The reaction was diluted in water (10 mL) and extracted into ethyl acetate (30 mL x3), the combined organic layers were dried with anhydrous magnesium sulfate, filtered and concentrated *in vacuo*. The crude residue was purified by column chromatography ($EtO_2$) to yield 2-(3-(dimethylamino)phenyl)-N-methoxy-N-methylacetamide (1.966 g, 8.84 mmol, 54 %). $^1$H NMR (400 MHz, $CDCl_3$) $\delta_H$ 7.17 (1H, t, $J$ = 7.9 Hz, ArC**H**), 6.73-6.59 (3H, m, ArC**H**), 3.73 (3H, s, C**H**$_2$), 3.59 (3H, s, OC**H**$_3$), 3.19 (3H, s, C**H**$_3$), 2.94 (6H, s, C**H**$_3$); $^{13}$C NMR (100 MHz, $CDCl_3$) $\delta_C$ 172.9 (**C**=O), 150.9 (Ar**C**H), 135.8 (Ar**C**$_q$), 129.2 (Ar**C**H), 117.7 (Ar**C**$_q$), 113.5 (Ar**C**H), 111.3 (Ar**C**H), 61.4 (O**C**H$_3$), 40.7 (**C**H$_3$), 40.0 (**C**H$_2$), 32.4 (**C**H$_3$); FTIR (ATR) $\upsilon_{max}$: 3436, 3351, 2936, 1636, 1602, 1461, 1382, 1096 cm$^{-1}$; HRMS (ESI) m/z: $[M+H]^+$ Calcd. for $C_{12}H_{19}N_2O_2^+$ 223.1441; Found 223.1444.

**1-(3-(dimethylamino)phenyl)propan-2-one (8a)**

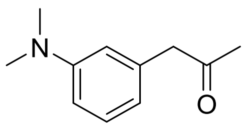

To a solution of 2-(3-(dimethylamino)phenyl)-*N*-methoxy-*N*-methylacetamide **I-4** (1.900 g, 8.5 mmol), in dry THF (20 mL) at -20 °C was added methyl magnesium bromide (4.3 mL, 3 M solution in $CH_2Cl_2$, 12.8 mmol) dropwise and the reaction mixture was warmed to room temperature and was stirred for 16 h. Water (10 mL) was added to the reaction and the mixture was basified and extracted into with dichloromethane (20 mL x3). The combined organic layers were dried with anhydrous magnesium sulfate, filtered and concentrated *in vacuo* to give 1-(3-(dimethylamino)phenyl)propan-2-one (1.27 g, 7.17 mmol, 84 %) with no further purification. $^1$H NMR (500 MHz, $CDCl_3$) $\delta_H$ 7.20 (1H, t, $J$ = 7.8 Hz, ArC**H**), 6.68 – 6.62 (1H, m, ArC**H**), 6.60 – 6.52 (2H, m, ArC**H**), 3.63 (2H, s, C**H**$_2$), 2.94 (6H, s, C**H**$_3$), 2.14 (3H, s, C**H**$_3$); $^{13}$C NMR (126 MHz, $CDCl_3$) $\delta_C$ 207.2 (**C**=O), 151.1 (Ar**C**$_q$), 135.3 (Ar**C**$_q$), 129.6 (Ar**C**H), 117.7 (Ar**C**$_q$), 113.4 (Ar**C**H), 111.4 (Ar**C**$_q$), 51.9 (**C**H$_3$), 40.7 (**C**H$_3$), 29.2 (**C**H$_2$); FTIR (ATR) $\upsilon_{max}$: 2920, 2803, 1706, 1600, 1497, 1352 cm$^{-1}$; HRMS (ESI) m/z: $[M+H]^+$ Calcd. for $C_{11}H_{16}NO^+$ 178.1226; Found 178.1230. In accordance to literature data.[3]

**3-(2-aminopropyl)-*N,N*-dimethylaniline (9a)**

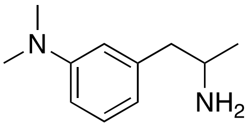

1-(3-(Dimethylamino)phenyl)propan-2-one **8a** (200 mg, 1.23 mmol), sodium cyanoborohydride (71 mg, 1.23 mmol) and ammonium acetate (870 mg, 11.28 mmol) were dissolved in methanol (10 mL), the reaction mixture was stirred overnight. The reaction was diluted with water (10 mL) and the methanol removed *in vacuo* before being basified to pH 9 with sodium hydroxide (5 M), the aqueous layer was washed with ethyl acetate (20 mL, x3). The combines organic layers were dried with anhydrous magnesium sulfate, filtered and concentrated *in vacuo*. The crude product was purified by column chromatography ($CH_2Cl_2$:MeOH (95:5)) to give title compound as a brown oil (70 mg, 39 %). $^1$H NMR (400 MHz, $CDCl_3$) $\delta_H$ 7.17 (1H, t, *J* = 8.0 Hz, ArC**H**), 6.63 – 6.50 (1H, m, ArC**H**), 6.58 – 6.54 (2H, m, ArC**H**), 3.34 – 3.26 (1H, m, C**H**), 3.14 (1H, s, N**H**$_2$), 2.94 (6H, s, C**H**$_3$), 2.78 – 2.63 (1H, m, C**H**$_2$), 1.17 (3H, d, *J* = 6.4 Hz, C**H**$_3$); $^{13}$C NMR (101 MHz, $CDCl_3$) $\delta_C$ 150.8 (Ar**C**$_q$), 139.3 (Ar**C**$_q$), 129.2 (Ar**C**H), 117.5 (Ar**C**H), 113.5 (Ar**C**H), 110.8 (Ar**C**H), 48.7 (**C**H), 45.4 (**C**H$_2$), 40.6 (**C**H$_3$), 21.9 (**C**H$_3$); FTIR (ATR) $\upsilon_{max}$: 3349, 2957, 2802, 1601, 1497, 1348, 1060 cm$^{-1}$; HRMS (ESI) m/z: $[M+H]^+$ Calcd. for $C_{11}H_{19}N_2^+$ 179.1543 Found 179.1556.

**1-(3-Hydroxyphenyl)propan-2-one (8c)**

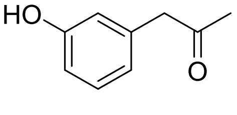

A solution of hydrogen bromide (9 mL, 48 % solution in water) and commercially available 1-(3-methoxyphenyl)propan-2-one **8b** (500 mg, 3.04 mmol) was heated at 120 °C for 2 h. Once cooled the reaction was diluted with water (10 mL) and neutralised with sat. sodium hydrogen carbonate solution before being extracted into dichloromethane (20 mL X3), the combine organic extracts where dried over anhydrous magnesium sulfate, filtered and concentrated *in vacuo* (water bath at room temperature). The crude product was then purified by column chromatography ($Et_2O$:pentane (3:7)) to yield the product as a yellow oil (267 mg, 1.78 mmol, 59%). $^1$H NMR (400 MHz, $CDCl_3$) $\delta_H$ 7.20 (1H, t, *J* = 7.8 Hz, ArC**H**), 6.79 – 6.73 (2H, m, ArC**H**), 6.70-6.67 (1H, m, ArC**H**), 3.65 (2H, s, C**H**$_2$), 2.16 (3H, s, C**H**$_3$); $^{13}$C NMR (126 MHz, $CDCl_3$) $\delta_C$ 207.6 (**C**=O), 156.3 (Ar**C**$_q$), 135.7 (Ar**C**$_q$), 130.2 (Ar**C**H), 121.7 (Ar**C**H), 116.4 (Ar**C**H), 114.5 (Ar**C**H), 51.0 (**C**H$_2$), 29.4 (**C**H$_3$); FTIR (ATR) $\upsilon_{max}$: 3335, 2962, 1696, 1587, 1356, 1156 cm$^{-1}$; HRMS (ESI) m/z: $[M+Na]^+$ Calcd. for $C_9H_{10}NaO_2^+$ 173.0573; Found 173.0574.

**3-(2-Aminopropyl)phenol (9c)**

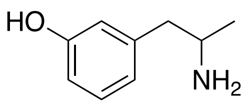


1-(3-(hydroxyphenyl)propan-2-one **8c** (100 mg, 0.66 mmol), sodium cyanoborohydride (46 mg, 0.80 mmol) and ammonium acetate (1.00 g, 13.2 mmol) were dissolved in methanol (10 mL), the reaction mixture was stirred overnight. The reaction was diluted with water (10 mL) and the methanol removed *in vacuo* before being basified to pH 9 with sodium hydroxide (5M), the aqueous layer was washed with ethyl acetate (20 mL, x3). The combines organic layers were dried with anhydrous magnesium sulfate, filtered and concentrated *in vacuo*. The crude product was purified by column chromatography (EtOAc:MeOH ($NH_3$ 7 N) (95:5)) to give title compound as a white solid (30 mg, 30 %). $^1$H NMR (400 MHz, MeOD) $\delta_H$ 7.09 (1H, t, *J* = 8.0 Hz, ArC**H**), 6.67 – 6.60 (3H, m, ArC**H**), 3.09 (1H, dt, *J* = 7.5, 6.2 Hz, C**H**), 2.62 – 2.49 (2H, m, C**H**$_2$), 1.08 (3H, d, *J* = 6.4 Hz, C**H**$_3$); $^{13}$C NMR (101 MHz, MeOD) $\delta_C$ 158.8 (ArC$_q$), 142.0 (ArC$_q$), 130.3 (ArC**H**), 121.3 (ArC**H**), 117.1 (ArC**H**), 114.4 (ArC**H**), 49.5 (C**H**), 46.5 (C**H**$_2$), 22.4 (C**H**$_3$); FTIR (ATR) $\upsilon_{max}$: 3342, 3289, 2961, 2854, 1592, 1454, 1259 cm$^{-1}$; HRMS (ESI) m/z: $[M+H]^+$ Calcd. for $C_9H_{14}NO^+$ 152.1070; Found 152.1075.

## Biotransformations for ATA-Knorr Pyrrole cascade reaction

### General Procedure for Optimization Studies: Analytical-Scale Biotransformations for the Preparation of Pyrrole (5a)[4]

The corresponding amount of *β*-amino-ester **1a** (5 - 100 mM) was dissolved in a HEPES buffer (100 mM, pH 5 -9) containing PLP (1 mM), and the resulting stock was then pH adjusted before 250 µL of the solution was aliquoted into separate microcentrifuge tubes. 1-Phenylpropane-1,2-dione **2a** (from a stock solution in DMSO) was added to the tubes, after which 200 µL of enzyme stock solution (commercially available ATA117 rehydrated in HEPES buffer containing PLP (1 mM)), to give a total volume 500 µL. The resulting mixture was incubated at 30 °C for 72 h in a shaking incubator (200 rpm). 100 µL samples were taken at 24 h intervals, the samples where diluted to a total volume of 500 µL and analyzed *via* reverse phase HPLC using a Waters Sunfire 5 µm, 2.1 x 150 mm column (C-18) at a flow rate of 0.6 mL min$^{-1}$ with a 40 to 100 % gradient.

**Table S1.** Optimization of biocatalytic Knorr-pyrrole synthesis

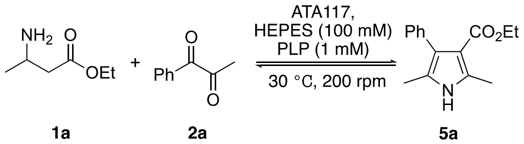


| Entry | Conc. of **2** (mM) | Enzyme loading (mg/mL$^{-1}$) | pH | Conv. (%) 24 h | Conv. (%) 48 h |
|---|---|---|---|---|---|
| 1 | 5 | 20 | 7 | 59 | 73 |
| 2 | 5 | 10 | 7 | 39 | 56 |
| 3 | 5 | 5 | 7 | 34 | 48 |
| 4 | 5 | 2 | 7 | 32 | 41 |
| 5 | 5 | 1 | 7 | 28 | 40 |
| 6 | 5 | 5 | 5 | 0 | 1 |
| 7 | 5 | 5 | 6 | 13 | 30 |
| 8 | 5 | 5 | 7 | 34 | 48 |
| 9 | 5 | 5 | 8 | 68 | 71 |
| 10 | 5 | 5 | 9 | 77 | 75 |
| 11 | 10 | 5 | 9 | 88 | 90 |
| 12 | 20 | 5 | 9 | 86 | 90 |
| 13 | 30 | 5 | 9 | 94 | 93 |
| 14 | 40 | 5 | 9 | 94 | 96 |
| 15 | 50 | 5 | 9 | 91 | 93 |
| 16 | 60 | 5 | 9 | 79 | 78 |
| 17 | 70 | 5 | 9 | 68 | 65 |
| 18 | 80 | 5 | 9 | 64 | 65 |
| 19 | 90 | 5 | 9 | 46 | 47 |
| 20 | 100 | 5 | 9 | 36 | 41 |

*Reaction conditions: 1-phenylpropane-1,2-dione **2a**, ethyl 3-aminobutanoate **1a** (2 racemic equiv. 1 equiv. **R-1a** available to enzyme), HEPES (100 mM, 0.5 mL), DMSO (10 % v/v), 30 °C, 200 rpm. Conversion was measured by HPLC. Results are the mean of three replicates.*

**General Procedure for Preparative-Scale Biotransformations: Preparation of Pyrroles (5a-l)**[4]

Commercially available ATA117 (5 mg/mL) was rehydrated in HEPES buffer (9.5 mL, 100 mM, pH 9) containing PLP (1 mM) and the corresponding *β*-amino-ester (*R*)/(*S*)-**1a** (for **5a**-**f**) or (*R*)-**1b** (for **5g**-**l**) (80 mM or 40 mM respectively). To this, was added the corresponding diketone **2a-f** (1 mL, 400 mM stock in DMSO) and the mixture was incubated at 30 °C for 72 h in a shaking incubator (200 rpm). The reaction mixture was acidified to pH 2 using aq. HCl (4 M, 1 mL) and extracted with EtOAc (3 x 20 mL). The combined organic layers were washed with water (20 mL), dried over anhydrous sodium sulfate and the solvent removed *in vacuo*. The corresponding pyrrole were purified by flash column chromatography (**5a**-**d** and **5f)** or preparative HPLC (**5e** and **5k**).

**Table S2.** Preparative-scale reactions between enantiopure ß-amino methyl ester **1b** and a range of diketones **2a-f**.

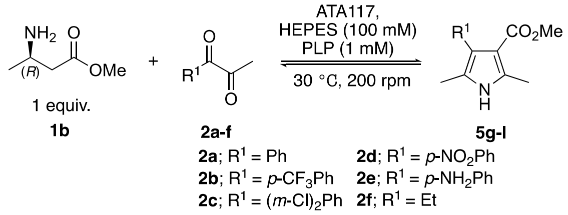


| Entry | Product | $R^1$ | ß-amino ester | Conv. (%) 72 h[a] | Yield (%) |
|---|---|---|---|---|---|
| 1 | g | Ph | 1 | 87 | 55[b] |
| 2 | h | *p*-$CF_3$Ph | 1 | 44 | 15[b] |
| 3 | i | (*m*-Cl)$_2$Ph | 1 | 95 | 80[b] |
| 4 | j | *p*-$NO_2$Ph | 1 | 44 | 32[b] |
| 5 | k | *p*-$NH_2$Ph | 1 | 76 | 55[c] |
| 6 | l | Et | 1 | 25 | 19[b] |

Reaction conditions: (*R*)-methyl 3-aminobutanoate **(*R*)-1b**, diketone **2a-f** (40 mM), HEPES (100 mM, pH 9), ATA117 (5 mg mL$^{-1}$), DMSO (10% v/v), 30 °C, 200 rpm, final volume of 10 mL. (a) Conversion measured by HPLC, (b) isolated yield after column chromatography, (c) isolated yield after preparative HPLC.1b to the α-diketone acceptor **2c**, generating reactive species **3b** and **4c**. The spontaneous Knorr pyrrole condensation functions to effectively displace the reaction equilibrium using stoichiometric equivalents (40 mM) of donor and acceptor, resulting in 95% conversion (80 % yield) to pyrrole **5i**.

**Representative example of a preparative-scale procedure**

**Ethyl 2,5-dimethyl-4-phenyl-1*H*-pyrrole-3-carboxylate (5a)**[4]

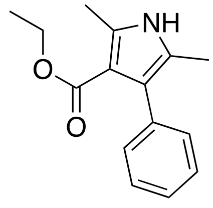


Commercially available ATA117 (50 mg, 5 mg/mL) was rehydrated in HEPES buffer (9 mL, 100 mM, pH 9) containing PLP (1 mM) and the ethyl 3-aminobutane (105 mg, 0.8 mmol, 80 mM). To this, was added a stock solution of 1-phenylpropane-1,2-dione (1 mL, 400 mM stock in DMSO) and the mixture was incubated at 30 °C for 72 h in a shaking incubator (200 rpm). The reaction mixture was acidified to pH 2 using aq. HCl (4 M, 1 mL) and extracted with EtOAc (3 x 20 mL). The combined organic layers were washed with water (20 mL), dried over anhydrous sodium sulfate and the solvent removed *in vacuo*. The crude product was purified by flash column chromatography ($CH_2Cl_2$) to give **5a** was obtained as a white solid (51 mg, 52 %). m.p. 105 - 107 °C; $^1$H NMR (500 MHz, MeOD) $\delta_H$ 7.30 – 7.25 (2H, m, ArC**H**), 7.22 – 7.15 (3H, m, ArC**H**), 4.00 (2H, q, *J* = 7.1 Hz, OC**H**$_2$CH$_3$), 2.45 (3H, s, C**H**$_3$), 2.05 (3H, s, C**H**$_3$), 1.04 (3H, t, *J* = 7.1 Hz, OCH$_2$C**H**$_3$); $^{13}$C NMR (126 MHz,

MeOD) $\delta_C$ 168.2 (**C**=O), 138.3 (Ar**C**$_q$), 135.8 (Ar**C**$_q$), 131.6 (Ar**C**$_q$), 128.2 (Ar**C**$_q$), 126.6 (Ar**C**$_q$), 124.9 (Ar**C**$_q$), 123.5 (Ar**C**$_q$), 110.6 (Ar**C**$_q$), 60.0 (O**C**H$_2$CH$_3$), 14.4 (**C**H$_3$), 13.5 (**C**H$_3$), 11.0 (OCH$_2$**C**H$_3$); FTIR (ATR) $\upsilon_{max}$: 3262, 2982, 2906, 1655, 1382, 1290, 1082, 751, 698 cm$^{-1}$; HRMS (ESI) m/z: $[M+H]^+$ Calcd. for $C_{15}H_{18}O_2^+$ 244.1332; Found 244.1328. In accordance to literature data.[4]

## Characterisation of pyrroles (5b-f)

### Ethyl 2,5-dimethyl-4-(4-(trifluoromethyl)phenyl-1*H*-pyrrole-3-carboxylate (5b)[4]

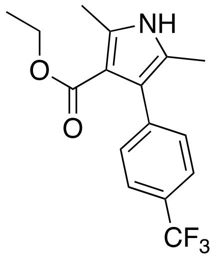


The reaction of 1-(4-(trifluoromethyl)phenyl)propane-1,2-dione **2b** (40 mM) and ethyl 3-aminobutanoate **1a** (80 mM) with ATA117 proceeded in 99 % conversion after 72 h. **5b** was obtained as a pale yellow solid (80 mg, 64 %) after flash chromatography ($CH_2Cl_2$). Due to co-elution, compound produced was not fully pure. m.p. 88 - 90 °C; $^1$H NMR (500 MHz, MeOD) $\delta_H$ 7.53 – 7.41 (4H, m, ArC**H**), 4.01 (2H, q, *J* = 7.1 Hz, OC**H**$_2$CH$_3$), 2.47 (3H, s, C**H**$_3$), 2.07 (3H, s, C**H**$_3$), 1.02 (3H, t, *J* = 7.1 Hz, OCH$_2$C**H**$_3$); $^{13}$C NMR (126 MHz, MeOD) $\delta_C$ 167.7 (**C**=O), 139.4 (Ar**C**$_q$), 136.5 (Ar**C**$_q$), 135.2 (ArCH), 129.0 (ArCH), 128.3 (q, *J* = 3.9 Hz, Ar**CF$_3$**), 125.6 (Ar**C**$_q$), 123.3 (Ar**C**$_q$), 121.9 (Ar**C**$_q$), 110.5 (Ar**C**$_q$), 60.1 (O**C**H$_2$CH$_3$), 14.3 (**C**H$_3$), 13.4 (**C**H$_3$), 10.9 (OCH$_2$**C**H$_3$); FTIR (ATR) $\upsilon_{max}$: 3317, 2949, 2865, 1673, 1445, 1327, 1013 cm$^{-1}$; HRMS (ESI) m/z: $[M+H]^+$ Calcd. for $C_{16}H_{17}F_3NO_2^+$ 312.1206; Found 312.1212. In accordance to literature data.[4]

### Ethyl 4-(3,5-dichlorophenyl)-2,5-dimethyl-1*H*-pyrrole-3- carboxylate (5c)[4]

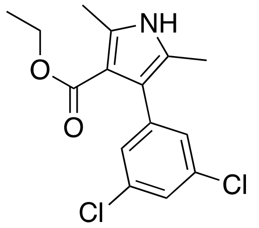


The reaction of 1-(3,5-dichlorophenyl)propane-1,2-dione **2c** (40 mM) and ethyl 3-aminobutanoate **1a** (80 mM) with ATA117 proceeded in 78 % conversion after 72 h. **5c** was obtained as a pale yellow solid (58 mg, 46 %) after flash chromatography ($CH_2Cl_2$). Due to co-elution, compound produced was not fully pure. m.p. 103 - 105 °C; $^1$H NMR (500 MHz, MeOD) $\delta_H$ 7.27 (1 H, t, *J* = 1.9 Hz, ArC**H**), 7.13 (2H, d, *J* = 2.0 Hz, ArC**H**), 4.05 (2H, q, *J* = 7.1 Hz, OC**H**$_2$CH$_3$), 2.45 (3H, s, C**H**$_3$), 2.08 (3H, s, C**H**$_3$), 1.11 (3H, t, *J* = 7.1 Hz, OCH$_2$C**H**$_3$); $^{13}$C NMR (126 MHz, MeOD) $\delta_C$ 166.1 (**C**=O), 140.4 (Ar**C**$_q$), 133.3 (Ar**C**$_q$), 128.8 (ArCH), 126.5 (ArCH), 125.0 (Ar**C**$_q$), 123.8 (Ar**C**$_q$), 119.3 (Ar**C**$_q$), 110.4 (Ar**C**$_q$), 58.8 (O**C**H$_2$CH$_3$), 13.0 (**C**H$_3$), 12.0 (**C**H$_3$), 9.5 (OCH$_2$**C**H$_3$); FTIR (ATR) $\upsilon_{max}$: 3294, 3075, 2981, 2938, 1662, 1587, 1557, 1419, 1096, 1016, 799 cm$^{-1}$; HRMS (ESI) m/z: $[M+H]^+$ Calcd. for $C_{15}H_{16}Cl_2NO_2^+$ 312.0553; Found 312.0547. In accordance to literature data.[4]

### Ethyl 2,5-dimethyl-4-(4-nitrophenyl)-1*H*-pyrrole-3-carboxylate (5d)

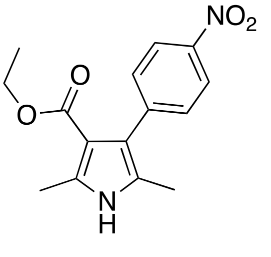


The reaction of 1-(4-nitrophenyl)propane-1,2-dione **2d** (40 mM) and ethyl 3-aminobutanoate **1a** (80 mM) with ATA117 proceeded in 41 % conversion after 72 h. **5d** was obtained as a pale yellow solid (29 mg, 25 %) after flash chromatography ($CH_2Cl_2$). Due to co-elution, compound produced was not fully pure. m.p. 123 - 125 °C; $^1$H NMR (500 MHz, MeOD) $\delta_H$ 8.18 (2H, d, *J* = 8.8 Hz, ArC**H**), 7.42 (2H, d, *J* = 8.8 Hz, ArC**H**), 4.06 (2H, q, *J* = 7.1 Hz, OC**H**$_2$CH$_3$), 2.46 (3H, s, C**H**$_3$), 2.11 (3H, s,

C**H**$_3$), 1.10 (3H, t, *J* = 7.1 Hz, OCH$_2$C**H**$_3$); $^{13}$C NMR (126 MHz, MeOD) $\delta_C$ 167.5 (**C**=O), 147.2 (Ar**C**$_q$), 145.8 (Ar**C**$_q$), 136.8 (Ar**C**$_q$), 132.4 (Ar**C**H), 126.3 (Ar**C**H), 123.5 (Ar**C**H), 121.5 (Ar**C**$_q$), 110.5 (Ar**C**$_q$), 60.3 (O**C**H$_2$CH$_3$), 14.5 (**C**H$_3$), 13.5 (**C**H$_3$), 11.0 (OCH$_2$**C**H$_3$); FTIR (ATR) $\upsilon_{max}$: 3306, 2980, 2923, 1773, 1668, 1593, 1511, 1341, 1089 cm$^{-1}$; HRMS (ESI) m/z: [M+H]$^+$ Calcd. for $C_{15}H_{17}N_2O_4^+$ 298.1183; Found 298.1193.

**Ethyl 2,5-dimethyl-4-(4-nitrophenyl)-1*H*-pyrrole-3-carboxylate (5e)**

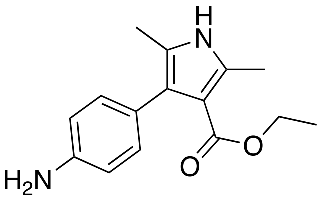

The reaction of 1-(4-amino)propane-1,2-dione **2e** (40 mM) and ethyl 3-aminobutanoate **1a** (80 mM, 3 mL) with ATA117 proceeded in 83 % conversion after 72 h. The acidified (pH 2) reaction mixture was centrifuged (10 min, 4000 rpm) before being directly injected onto the preparative HPLC (20 to 80 % gradient), where **5e** was obtained as a pale yellow solid (17 mg, 54 %). m.p. degrades at 147 °C; $^1$H NMR (400 MHz, MeOD) $\delta_H$ 7 7.37 – 7.28 (4H, m, ArC**H**), 4.04 (2H, q, *J* = 7.1 Hz, OC**H**$_2$CH$_3$), 2.46 (3H, s, C**H$_3$**), 2.06 (3H, s, C**H$_3$**), 1.10 (3H, t, *J* = 7.1 Hz, OCH$_2$C**H**$_3$); $^{13}$C NMR (101 MHz, MeOD) $\delta_C$ 167.8 (**C**=O), 139.1 (Ar**C**$_q$), 136.3 (Ar**C**$_q$), 133.2 (Ar**C**H), 130.1 (Ar**C**$_q$), 125.5 (Ar**C**$_q$), 122.6 (Ar**C**H), 121.9 (Ar**C**$_q$), 110.5 (Ar**C**$_q$), 60.1 (O**C**H$_2$CH$_3$), 14.5 (OCH$_2$**C**H$_3$), 13.5 (**C**H$_3$), 10.9 (**C**H$_3$); FTIR (ATR) $\upsilon_{max}$: 2981, 2922, 1646, 1172, 1135 cm$^{-1}$; HRMS (ESI) m/z: [M+H]$^+$ Calcd. for $C_{14}H_{17}N_2O_2^+$ theoretical 259.1441; Found 259.1454.

**Ethyl 4-ethyl-2,5-dimethyl-1*H*-pyrrole-3-carboxylate (5f)**

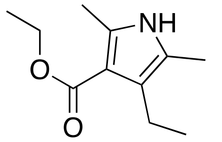

The reaction of pentane-1,2-dione **2f** (40 mM)and ethyl 3-aminobutanoate **1a** (80 mM) with ATA117 proceeded in 34 % conversion after 72 h. **5f** was obtained as a white solid (21 mg, 28 %) after flash chromatography (CH$_2$Cl$_2$). m.p. 94 - 96 °C;$^1$H NMR (500 MHz, MeOD) $\delta_H$ 4.20 (2H, q, *J* = 7.1 Hz, OC**H**$_2$CH$_3$), 2.59 (2H, q, *J* = 7.4 Hz, C**H**$_2$CH$_3$), 2.38 (3H, s, C**H**$_3$), 2.07 (3H, s, C**H**$_3$), 1.33 (3H, t, *J* = 7.1 Hz, OCH$_2$C**H**$_3$), 1.04 (3H, t, *J* = 7.4 Hz, CH$_2$C**H**$_3$); $^{13}$C NMR (126 MHz, MeOD) $\delta_C$ 168.5 (**C**=O), 135.5 (Ar**C**$_q$), 123.5 (Ar**C**$_q$), 123.0 (Ar**C**$_q$), 109.9 (Ar**C**$_q$), 60.0 (O**C**H$_2$CH$_3$), 19.4 (**C**H$_2$CH$_3$), 16.5 (**C**H$_3$), 14.8 (OCH$_2$**C**H$_3$), 13.8 (**C**H$_3$), 10.1 (CH$_2$**C**H$_3$); FTIR (ATR) $\upsilon_{max}$: 3270, 2924, 2422,1649, 1243, 1148, 1100 cm$^{-1}$; HRMS (ESI) m/z: [M+H]$^+$ Calcd. for $C_{11}H_{18}NO_2^+$ 196.1332 Found 196.1328.

**Methyl 2,5-dimethyl-4-phenyl-1*H*-pyrrole-3- carboxylate (5g)**[4]

The reaction of 1-phenylpropane-1,2-dione **2a** (40 mM) and ethyl 3-aminobutanoate **(*R*)-2b** (40 mM) with

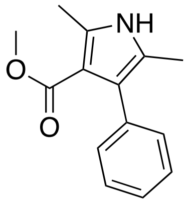

ATA117 proceeded in 87 % conversion after 72 h. **5g** was obtained as a white solid (50 mg, 55 %) after flash chromatography (CH$_2$Cl$_2$). m.p. 135 - 138 °C; $^1$H NMR (500 MHz, MeOD) $\delta_H$ 7.31 – 7.24 (2H, m, ArC**H**), 7.21 – 7.14 (3H, m, ArC**H**), 3.55 (3H, s, OC**H**$_3$), 2.45 (3H, s, C**H**$_3$), 2.05 (3H, s, C**H**$_3$); $^{13}$C NMR (126 MHz, MeOD) $\delta_C$ 168.5 (**C**=O), 138.1 (Ar**C**$_q$), 131.5 (Ar**C**$_q$), 131.5 (Ar**C**H), 128.3 (Ar**C**H), 126.6 (Ar**C**H), 125.1 (Ar**C**$_q$), 123.1 (Ar**C**$_q$), 110.4 (Ar**C**$_q$), 50.6 (O**C**H$_3$), 13.5 (**C**H$_3$), 11.0 (**C**H$_3$); FTIR (ATR) $\upsilon_{max}$: 3281, 2951, 2500, 1660, 1446, 1403, 1163,

1080 cm$^{-1}$; HRMS (ESI) m/z: $[M+H]^+$ Calcd. for $C_{14}H_{16}NO_2^+$ 230.1176; Found 230.1182. In accordance to literature data.[4]

**Methyl 2,5-dimethyl-4-(4-(trifluoromethyl)phenyl-1*H*-pyrrole-3-carboxylate (5h)**

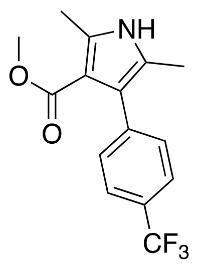


The reaction of 1-(4-(trifluoromethyl)phenyl)propane-1,2-dione **2b** (40 mM) and methyl 3-aminobutanoate **(*R*)-1b** (40 mM) with ATA117 proceeded in 44 % conversion after 72 h. **5h** was obtained as a pale yellow solid (18 mg, 15 %) after flash chromatography ($CH_2Cl_2$). Compound not fully pure due to degradation. m.p. 109-113 °C; $^1$H NMR (500 MHz, MeOD) $\delta_H$ 7.51 – 7.40 (4H, m, ArC**H**), 3.55 (3H, s, OC**H**$_3$), 2.46 (3H, s, C**H**$_3$), 2.07 (3H, s, C**H**$_3$); $^{13}$C NMR (126 MHz, $CDCl_3$) $\delta_C$ 168.1 (**C**=O), 139.1 (Ar**C**$_q$), 136.5 (Ar**C**H), 135.1 (Ar**C**$_q$), 129.0 (Ar**C**H), 128.2 (q, *J* = 3.9 Hz, Ar**CF$_3$**), 125.7 (Ar**C**$_q$), 123.2 (Ar**C**$_q$), 121.9 (Ar**C**$_q$), 110.2 (Ar**C**$_q$), 50.6 (O**C**H$_3$), 13.5 (**C**H$_3$), 10.9 (**C**H$_3$); FTIR (ATR) $\upsilon_{max}$: 3317, 2949, 455, 1672, 1327, 1113, 1069 cm$^{-1}$; HRMS (ESI) m/z: $[M+H]^+$ Calcd. for $C_{15}H_{15}F_3NO_2^+$ 298.1049; Found 298.1048.

**Methyl 4-(3,5-dichlorophenyl)-2,5-dimethyl-1*H*-pyrrole-3-carboxylate (5i)**

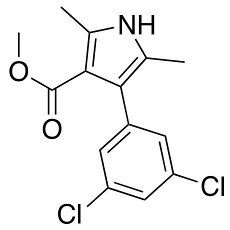


The reaction of 1-(3,5-dichlorophenyl)propane-1,2-dione **2c** (40 mM) and methyl 3-aminobutanoate **(*R*)-1b** (40 mM) with ATA117 proceeded in 95 % conversion after 72 h. **5i** was obtained as a pale yellow solid (93 mg, 80 %) after flash chromatography ($CH_2Cl_2$). Compound not fully pure due to degradation. m.p. 103 - 105 °C; $^1$H NMR (500 MHz, MeOD) $\delta_H$ 7.27 (1H, t, *J* = 2.0 Hz, ArC**H**), 7.12 (2H, d, *J* = 1.9 Hz, ArC**H**), 3.61 (3H, s, OC**H**$_3$), 2.44 (3H, s, C**H**$_3$), 2.08 (3H, s, C**H**$_3$); $^{13}$C NMR (126 MHz, $CDCl_3$) $\delta_C$ 167.9 (**C**=O), 141.7 (Ar**C**$_q$), 134.6 (Ar**C**$_q$), 130.0 (Ar**C**$_q$), 126.5 (Ar**C**H), 126.4 (Ar**C**H), 125.0 (Ar**C**$_q$), 120.8 (Ar**C**$_q$), 110.2 (Ar**C**$_q$), 50.8 (O**C**H$_3$), 13.5 (**C**H$_3$), 10.9 (**C**H$_3$); FTIR (ATR) $\upsilon_{max}$: 3320, 2945, 2450, 1674, 1415, 1170, 1094 cm$^{-1}$; HRMS (ESI) m/z: $[M+H]^+$ Calcd. for $C_{14}H_{14}Cl_2NO_2^+$ 298.0396; Found 298.0388.

**Methyl 2,5-dimethyl-4-(4-nitrophenyl)-1*H*-pyrrole-3-carboxylate (5j)**

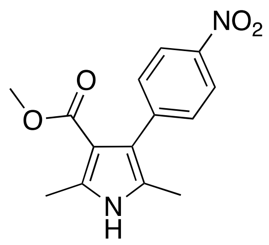


The reaction of 1-(4-nitrophenyl)propane-1,2-dione **2d** (40 mM) and methyl 3-aminobutanoate **(*R*)-1b** (40 mM) with ATA117 proceeded in 44 % conversion after 72 h. **5j** was obtained as a pale yellow solid (35 mg, 32 %) after flash chromatography ($CH_2Cl_2$). Compound not fully pure due to degradation. m.p. 145 - 149 °C; $^1$H NMR (500 MHz, MeOD) $\delta_H$ 8.17 (2H, d, *J* = 8.8 Hz, ArC**H**), 7.40 (2H, d, *J* = 8.8 Hz, ArC**H**), 3.59 (3H, s, OC**H**$_3$), 2.46 (3H, s, C**H**$_3$), 2.11 (3H, s, C**H**$_3$); $^{13}$C NMR (126 MHz, MeOD) $\delta_C$ 167.9 (**C**=O), 147.1 (Ar**C**$_q$), 145.6 (Ar**C**$_q$), 136.9 (Ar**C**$_q$), 132.2 (Ar**C**H), 126.4 (Ar**C**$_q$), 123.5 (Ar**C**H), 121.5 (Ar**C**$_q$), 110.3 (Ar**C**$_q$), 50.8 (O**C**H$_3$), 13.5 (**C**H$_3$), 11.1 (**C**H$_3$); FTIR (ATR) $\upsilon_{max}$: 3314, 2948, 2447, 1667, 1593, 1328, 1077 cm$^{-1}$; HRMS (ESI) m/z: $[M+H]^+$ Calcd. for $C_{14}H_{15}N_2O_4^+$ 275.1026; Found 275.1032.

**Methyl 2,5-dimethyl-4-(4-aminophenyl-1H-pyrrole-3-carboxylate (5k)**

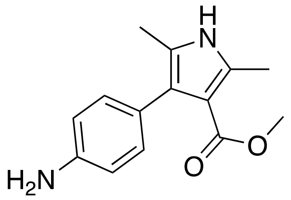


The reaction of 1-(4-amino)propane-1,2-dione **2e** (40 mM) and methyl 3-aminobutanoate **(*R*)-1b** (40 mM, 3 mL) with ATA117 proceeded in 76 % conversion after 72 h. The acidified reaction mixture was centrifuged (10 min, 4000 rpm) before being directly injected onto the preparative HPLC (20 to 80 % gradient), where **5k** was obtained as a pale yellow solid (16 mg, 55 %). m.p. degrades at 150 °C; $^1$H NMR (400 MHz, MeOD) $\delta_H$ 7.36 – 7.28 (4H, m, ArC**H**), 3.58 (3H, s, OC**H**$_3$), 2.45 (3H, s, C**H**$_3$), 2.06 (3H, s, C**H**$_3$); $^{13}$C NMR (101 MHz, MeOD) $\delta_C$ 168.1 (**C**=O), 139.1 (Ar**C**$_q$), 136.3 (Ar**C**$_q$), 133.1 (ArCH), 129.9 (Ar**C**$_q$), 125.6 (Ar**C**$_q$), 122.7 (Ar**C**H), 121.9 (Ar**C**$_q$), 110.2 (Ar**C**$_q$), 50.7 (O**C**H$_3$), 13.6 (**C**H$_3$), 10.9 (**C**H$_3$); FTIR (ATR) $\upsilon_{max}$: 2966, 2922, 1638, 1453, 1407, 1129 cm$^{-1}$; HRMS (ESI) m/z: [M+H]$^+$ Calcd. for $C_{14}H_{17}N_2O_2^+$ 245.1285; Found 245.1289.

**Methyl 4-ethyl-2,5-dimethyl-1*H*-pyrrole-3-carboxylate (5l)**

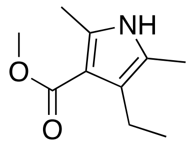


The reaction of pentane-1,2-dione **2f** (40 mM)and methyl 3-aminobutanoate **(*R*)-1b** (40 mM) with ATA117 proceeded in 25 % conversion after 72 h. **5l** was obtained as a white solid (14 mg, 19 %) after flash chromatography ($CH_2Cl_2$). m.p. 129 - 132 °C; $^1$H NMR (500 MHz, MeOD) $\delta_H$ 3.74 (3H, s, OC**H**$_3$), 2.58 (2H, q, *J* = 7.4 Hz, C**H**$_2$CH$_3$), 2.37 (3H, s, C**H**$_3$), 2.07 (3H, s, C**H**$_3$), 1.03 (3H, t, *J* = 7.4 Hz, 3 CH$_2$C**H**$_3$); $^{13}$C NMR (126 MHz, MeOD) $\delta_C$ 168.9 (**C**=O), 135.5 (Ar**C**$_q$), 123.6 (Ar**C**$_q$), 123.0 (Ar**C**$_q$), 109.7 (Ar**C**$_q$), 50.6 (O**C**H$_3$), 19.4 (**C**H$_2$CH$_3$), 16.4 (**C**H$_3$), 13.8 (**C**H$_3$), 10.1 (CH$_2$**C**H$_3$); FTIR (ATR) $\upsilon_{max}$: 3294, 2954, 2865, 2447, 1660, 1410, 1283, 1093 cm$^{-1}$; HRMS (ESI) m/z: [M+H]$^+$ Calcd. for $C_{10}H_{16}NO_2^+$ 182.1176; Found 182.1173.

## Procedure for analytical scale Pictet-Spengler reactions

A stock solution of 1-(3-(dimethylamino)phenyl)propan-2-one **8a** (200 mM) in a $KP_i$ buffer (100 mM) was then pH adjusted (pH 6 – 9) before 100 µL was aliquoted into separate microcentrifuge tubes. To this benzaldehyde stock (500 µL, 88 mM in co-solvent) was added, after which 400 µL of $KP_i$ buffer (100 mM) to give a total volume 1000 µL. The resulting mixture was incubated at 50 °C for 24 - 48 h in a shaking incubator (200 rpm). After completion, the pH of each solution was adjusted to 12 using aq. NaOH (50 µL, 10 M) and extracted with EtOAc (1000 µL). The resulting organic layer was analyzed by GC-FID *via* gradient **1**. See Figure 3 in the main article for results.

## Analytical scale synthesis of THIQ 11-13 *via* TA/Pictet-Spengler cascade

The corresponding amount of amine was dissolved in a $KP_i$ buffer (100 mM) containing PLP (1 mM) and the resulting stock was then pH adjusted before 250 µL was aliquoted into separate microcentrifuge tubes. To this ketone **8a-c** stock (in DMSO) was added, after which 200 µL of enzyme stock solution (commercially available ATA rehydrated in HEPES buffer containing PLP (1 mM)), to give a total volume 1000 µL. The resulting mixture was incubated at 50 °C for 48 h in a shaking incubator (200 rpm). 500 µL sample was taken, the samples where diluted to a total volume of 600 µL with a dDMSO containing sodium formate standard or maleic acid and analysed *via* NMR. Reaction conditions and results are shown in Table S3.

## Table S3. Optimisation of biocatalytic Pictet-Spengler reaction

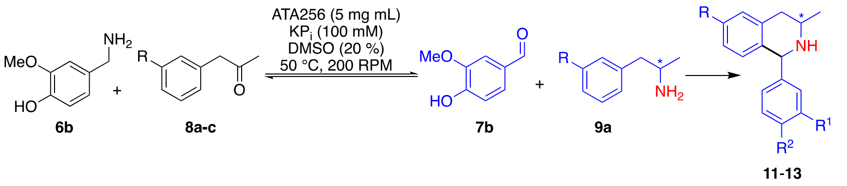


| Entry | R | Conc. of ketone **8a - c** (mM) | Equiv. amine **6a** | pH | % DMSO (v/v) | Conv. to amine **9a** (%) 48 h | Conv. to THIQ **11-13**[(a)] (%) 48 h |
|---|---|---|---|---|---|---|---|
| 1 | $NMe_2$ | 40 | 1.1 | 7 | 20 | 3 | 2 |
| 2 | $NMe_2$ | 40 | 1.1 | 7.5 | 20 | 3 | 1 |
| 3 | $NMe_2$ | 40 | 1.1 | 8 | 20 | 2 | 1 |
| 4 | $NMe_2$ | 40 | 2 | 7.5 | 20 | 4 | 2 |
| 5 | $NMe_2$ | 10 | 1.1 | 7.5 | 20 | 5 | 4 |
| 6 | $NMe_2$ | 50 | 1.1 | 7.5 | 20 | 3 | 2 |
| 7 | $NMe_2$ | 100 | 1.1 | 7.5 | 20 | 3 | 3 |
| 8 | OMe | 50 | 1.1 | 7.5 | 20 | 32 | 0 |
| 9 | OH | 40 | 1.1 | 6 | 20 | 17 | 7 |
| 10 | OH | 40 | 1.1 | 7 | 20 | 15 | 15 |
| 11 | OH | 40 | 1.1 | 7.5 | 20 | 14 | 17 |

| | | | | | | | |
|---|---|---|---|---|---|---|---|
| 12 | OH | 40 | 1.1 | 8 | 20 | 13 | 13 |
| 13 | OH | 40 | 1.1 | 9 | 20 | 13 | 8 |
| 14 | OH | 40 | 2 | 7.5 | 20 | 21 | 27 |
| 15 | OH | 40 | 5 | 7.5 | 20 | 27 | 31 |
| 16 | OH | 10 | 1.1 | 7.5 | 20 | 13 | 4 |
| 17 | OH | 25 | 1.1 | 7.5 | 20 | 14 | 12 |
| 18 | OH | 50 | 1.1 | 7.5 | 20 | 14 | 17 |
| 19 | OH | 75 | 1.1 | 7.5 | 20 | 15 | 20 |
| 20 | OH | 100 | 1.1 | 7.5 | 20 | 14 | 22 |
| 21 | OH | 100 | 1.1 | 7.5 | 15 | 11 | 26 |
| 22 | OH | 100 | 1.1 | 7.5 | 10 | 11 | 28 |
| 23 | OH | 100 | 1.1 | 7.5 | 5 | 10 | 22 |

*pH and concentration of substrate screen, with samples taken at 48 h. Reaction conditions, phenylacetone derivate **8a - c**, vanillamine **6b**, HEPES (100 mM, 0.5 mL), DMSO (v/v), 50 °C, 200 rpm. Conversion was measured by NMR against a standard. The results in the table are the mean of three replicates. (a) d.r. 2:3 measured via NMR.*

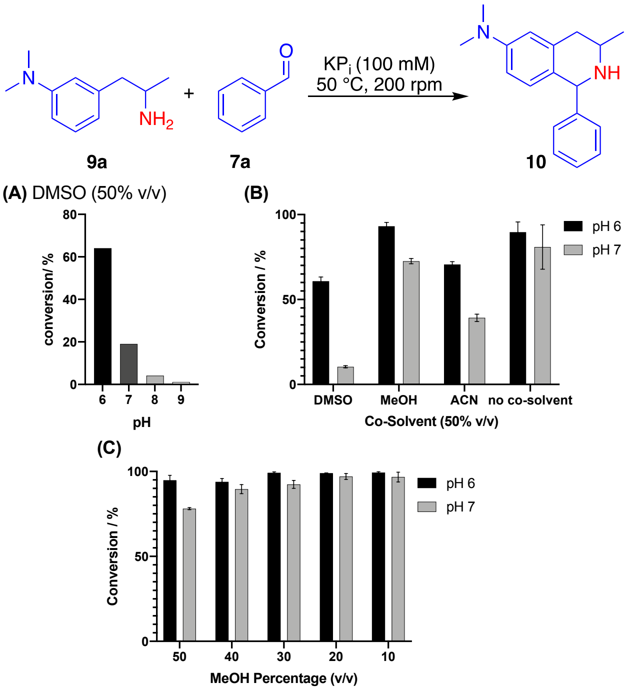


*Figure S1. Optimizing the Pictet-Spengler reaction conditions for the condensation of racemic **9a** and **7a**. The effect of pH in DMSO **(A)**, various co-solvents (50% v/v) **(B)** and % MeOH **(B)** on the conversion to THIQ **10**.*

**Table S4.** Concentration screen for substrates 8c and 6b

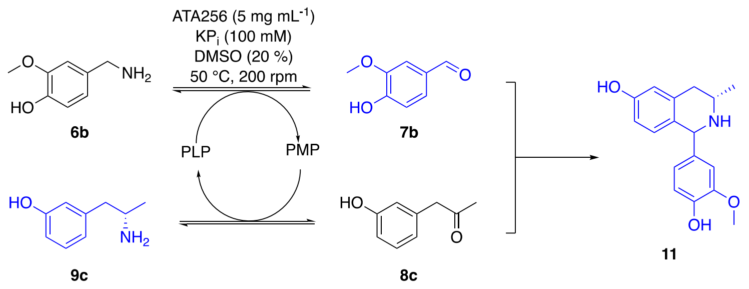


| Entry | Conc. of ketone **8c** (mM) | Conv. to amine **9c** (%) 48 h | Conv. to Vanillin **7b** (%) 48 h |
|---|---|---|---|
| 1 | 10 | 13 | 42 |
| 2 | 25 | 14 | 35 |
| 3 | 50 | 14 | 24 |
| 4 | 75 | 15 | 19 |
| 5 | 100 | 14 | 15 |

*Reaction conditions, m-1-(3-(hydroxyphenyl)propan-2-one* ***8c****, vanillamine* ***6b****, HEPES (100 mM, 0.5 mL), DMSO (20 % v/v), 50 °C, 200 rpm. Conversion was measured by NMR against a standard. The results in the table are the mean of three replicates. At lower concentrations of ketone* ***8c*** *there is a discrepancy between the conversion to amine* ***9c*** *and vanillin* ***7b****. This is likely amine* ***9c*** *decomposing faster than the PS reaction can occur.*

**Calculation used to work out the conversion of biotransformations *via* quantitative NMR analysis**

The quantitative integration of NMR spectra was undertaken by referencing peaks of interest to the known concentration of the standard peak, in this case, maleic acid. The peak for maleic acid is observed at 6.30 ppm in the spectra, and the integration of this peak was set to the concentration of maleic acid added (50 mM) x number of protons contributing to the signal (2).

This allowed quantitative examination of the other peaks of the NMR by referencing to this standard, as the relative integrations could then be divided by the number of protons present in the peak to give the relative concentration of the product or reactant.

To account for the dilution factor used in the experiment (where 100 μL was added to 500 μL of sample, to give 600 μL total volume), the below formula was used to determine the concentration of the reagent/product of interest:

$$conc.\ of\ reactant\ or\ product(mM) = \left(\frac{integration\ of\ peak}{no.\ of\ protons\ in\ peak}\right) \times \frac{6}{5}$$

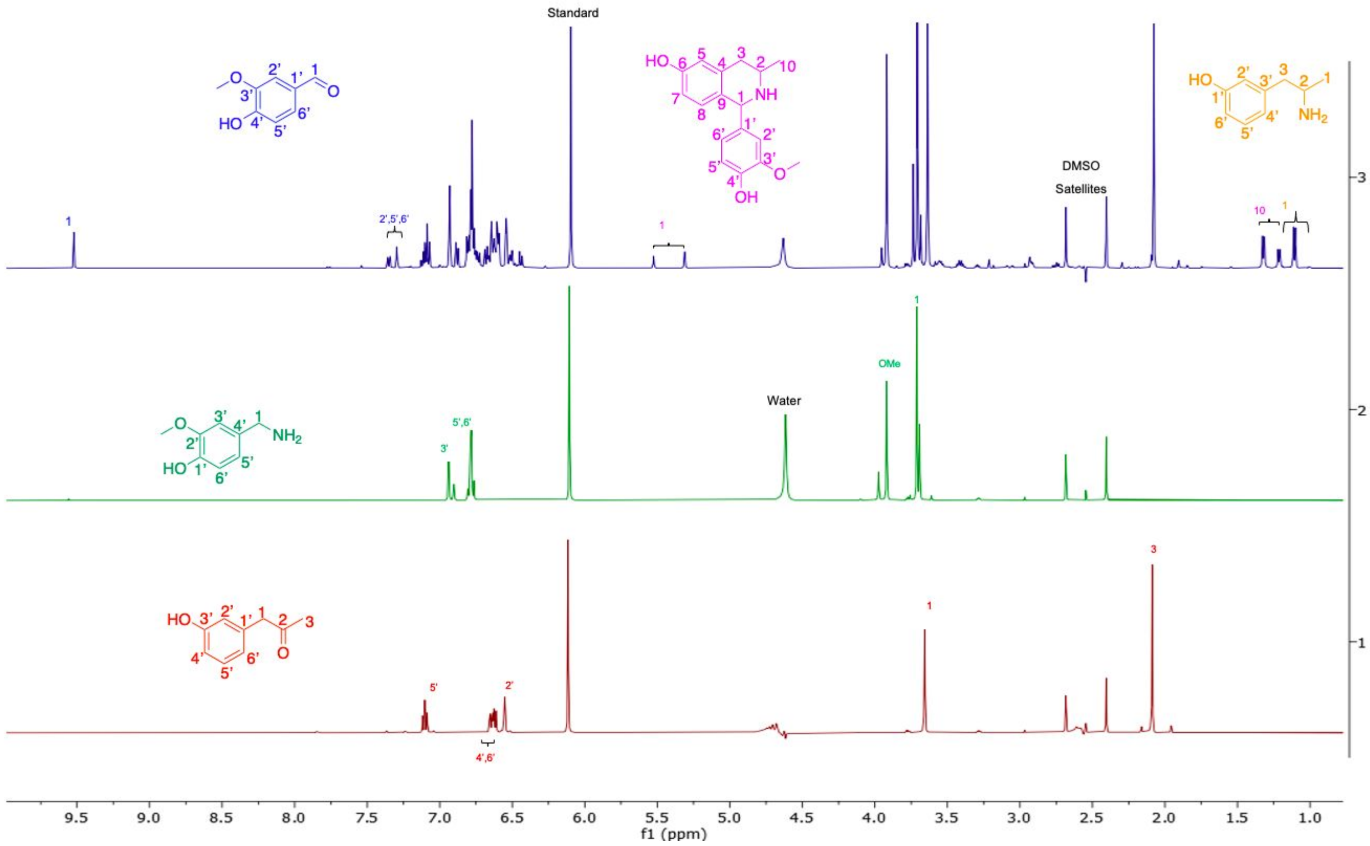

# $^{1}$H NMR and $^{13}$C NMR Spectra

## 2e

$^{1}$H NMR (400 MHz, $CDCl_3$) δ 7.85 (d, $J$ = 8.7 Hz, 2H), 6.64 (d, $J$ = 8.7 Hz, 2H), 4.33 (s, 2H), 2.47 (s, 3H).

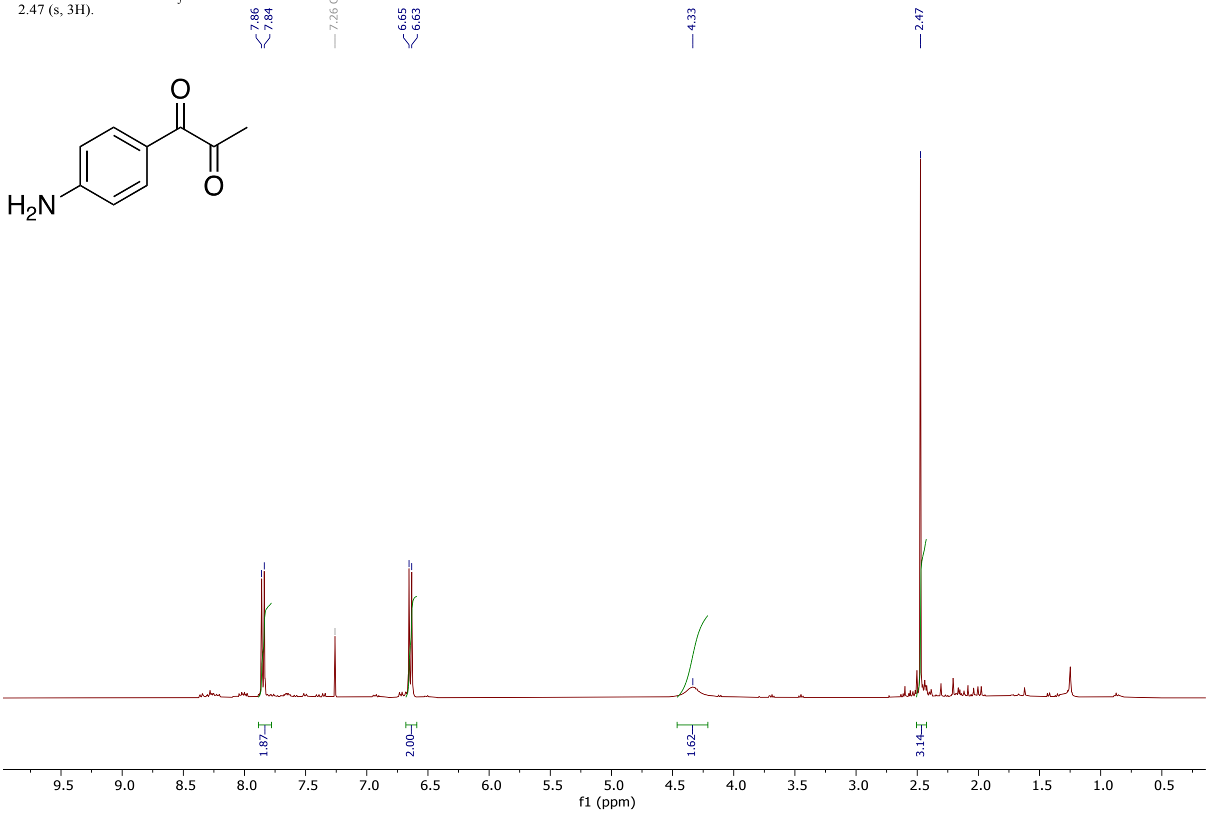


$^{13}$C NMR (101 MHz, $CDCl_3$) δ 202.2, 190.0, 152.8, 133.2, 121.8, 114.1, 26.8.

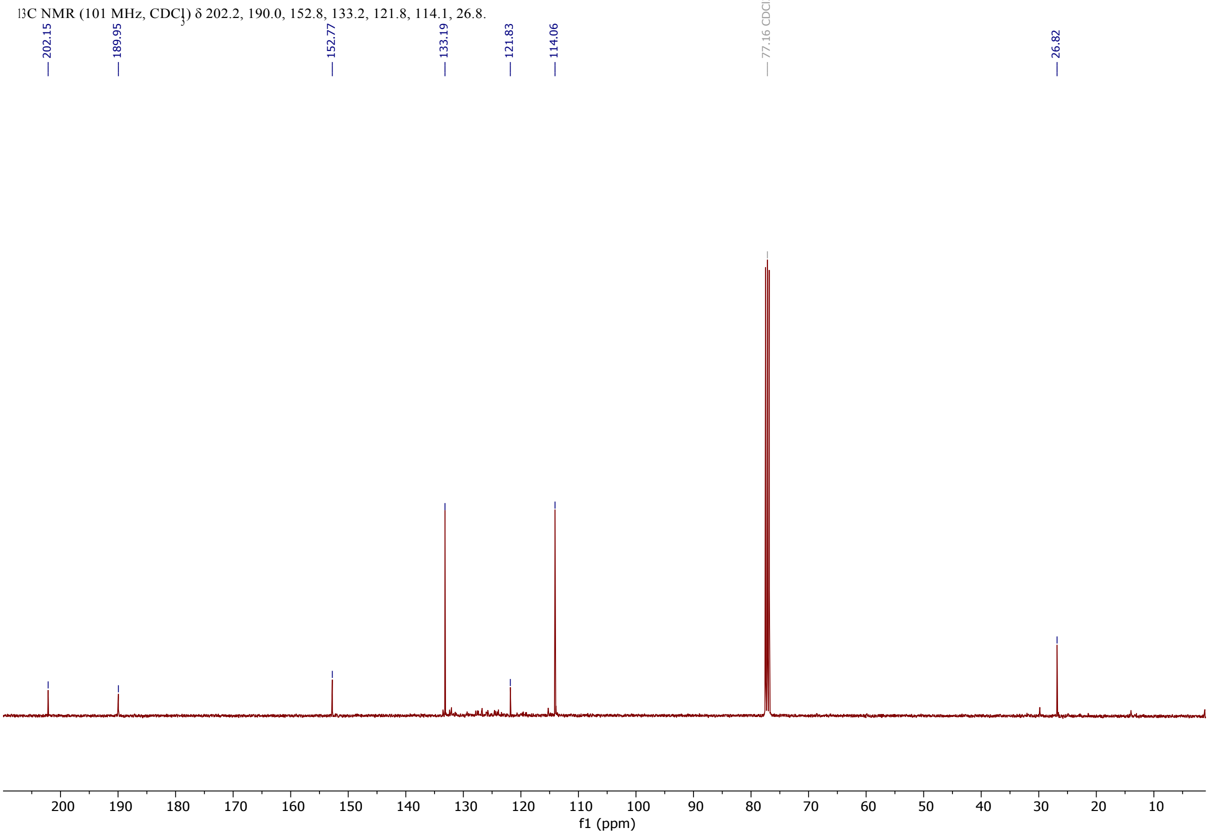

# 5a

$^{1}$H NMR (500 MHz, MeOD)δ 7.30 – 7.25 (m, 2H), 7.22 – 7.15 (m, 3H), 4.00 (q, $J$ = 7.1 Hz, 2H), 2.45 (s, 3H), 2.05 (s, 3H), 1.04 (t, $J$ = 7.1 Hz, 3H).

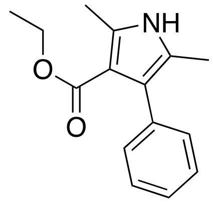


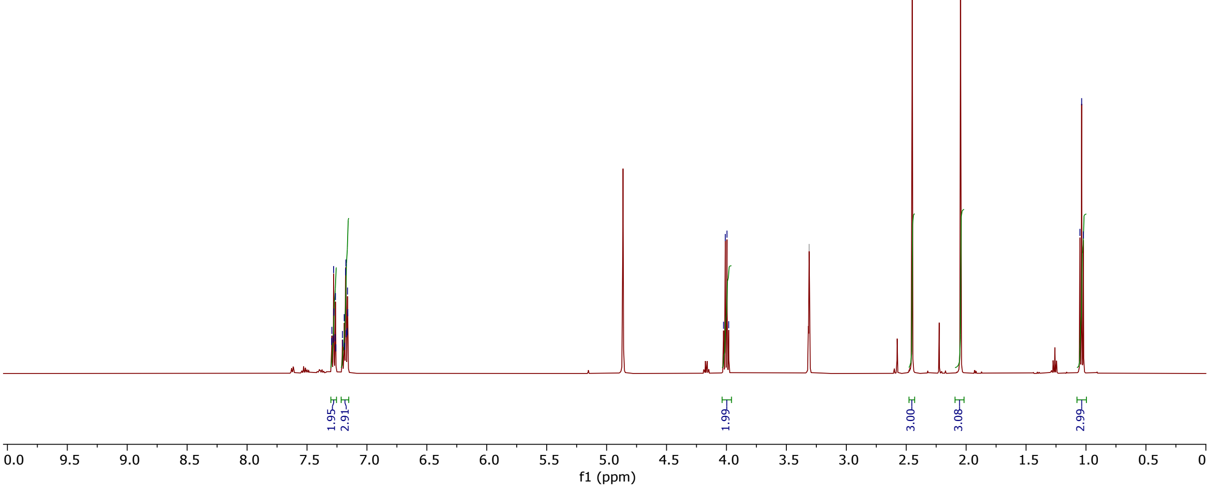


$^{13}$C NMR (126 MHz, MeOD)δ 168.2, 138.3, 135.8, 131.6, 128.2, 126.6, 124.9, 123.5, 110.6, 60.0, 14.4, 13.5, 11.0.

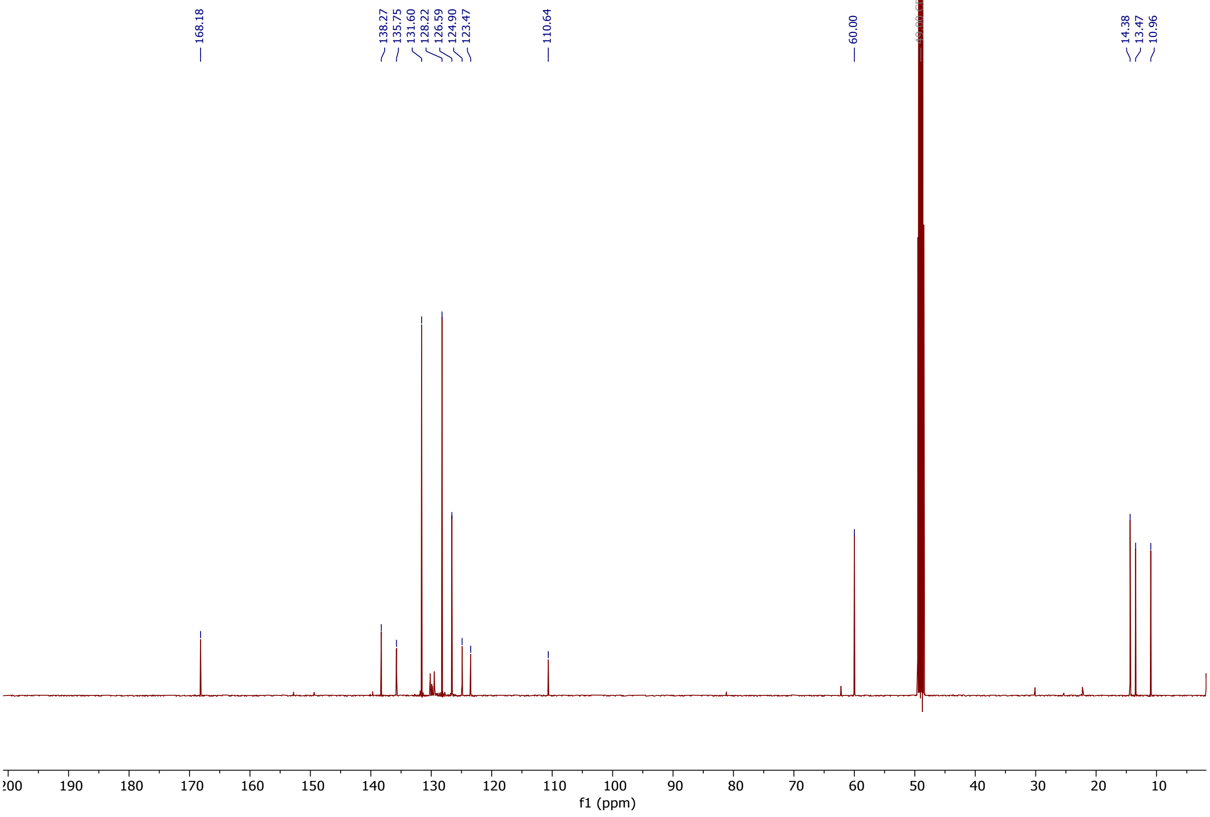

# 5b

1H NMR (500 MHz, MeOD)δ 7.53 – 7.41 (m, 4H), 4.01 (q, *J* = 7.1 Hz, 2H), 2.47 (s, 3H), 2.07 (s, 3H), 1.02 (t, *J* = 7.1 Hz, 3H).

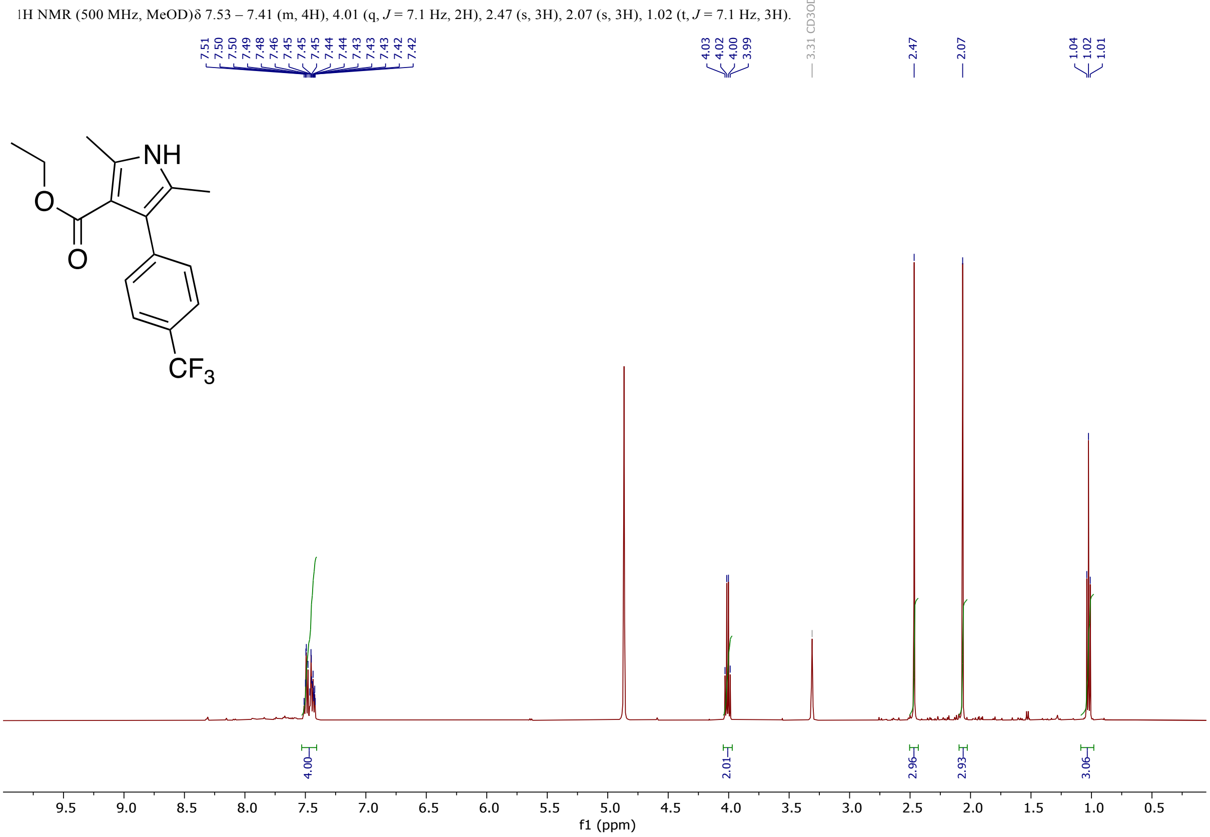


13C NMR (126 MHz, MeOD)δ 167.7, 139.4, 136.5, 135.2, 129.0, 128.3 (q, *J* =3.9) , 125.6, 123.3, 121.9, 110.5, 60.1, 14.3, 13.4, 10.9.

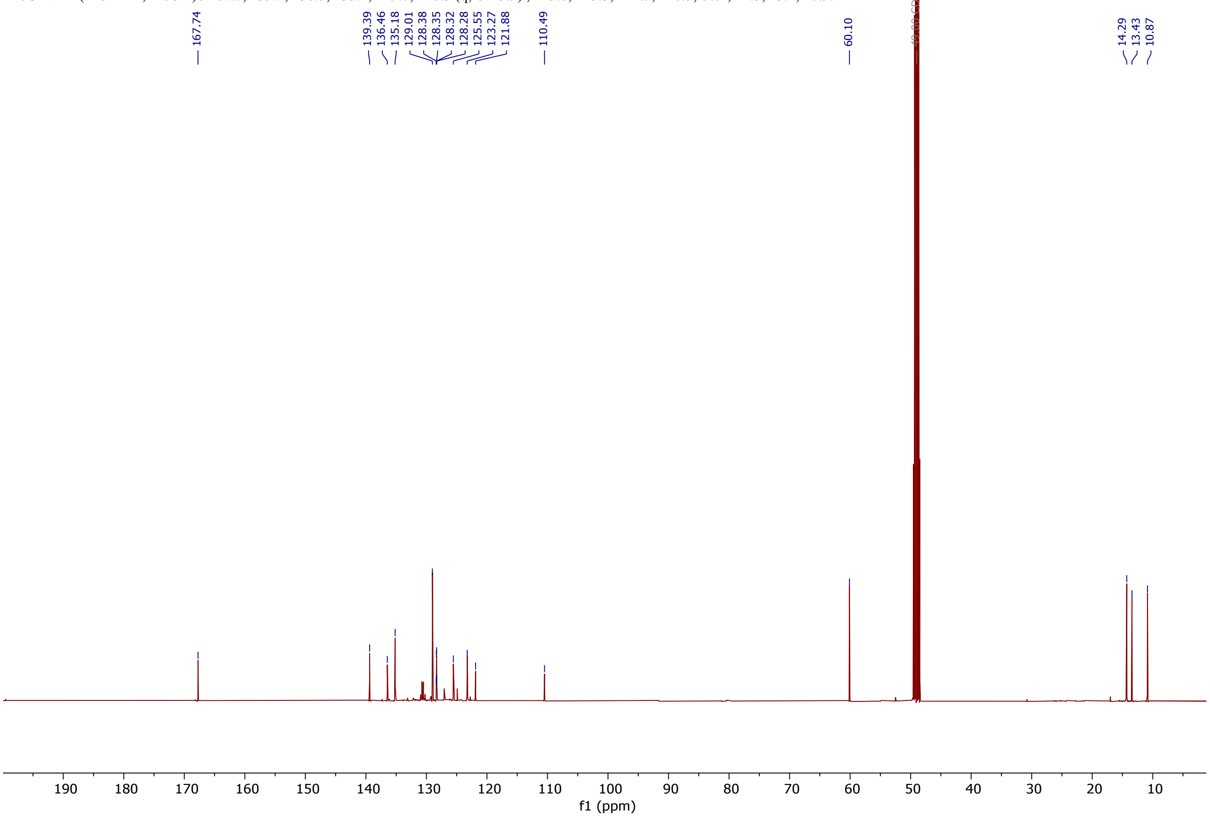

**5c**

$^1$H NMR (500 MHz, MeOD)δ 7.27 (t, $J$ = 1.9 Hz, 1H), 7.13 (d, $J$ = 2.0 Hz, 2H), 4.05 (q, $J$ = 7.1 Hz, 2H), 2.45 (s, 3H), 2.08 (s, 3H), 1.11 (t, $J$ = 7.1 Hz, 3H).

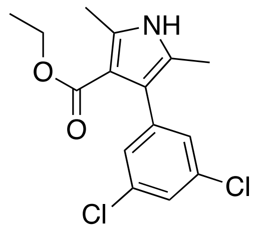

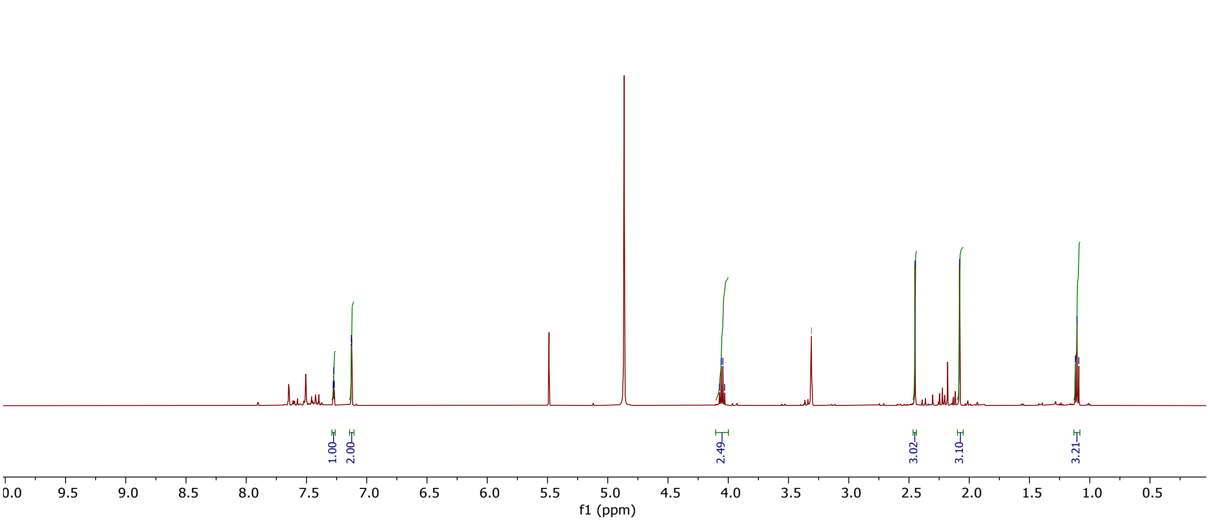


$^{13}$C NMR (126 MHz, MeOD)δ 166.1, 140.4, 133.3, 128.8, 126.5, 125.0, 123.8, 119.3, 58.8, 13.0, 12.0, 9.5.

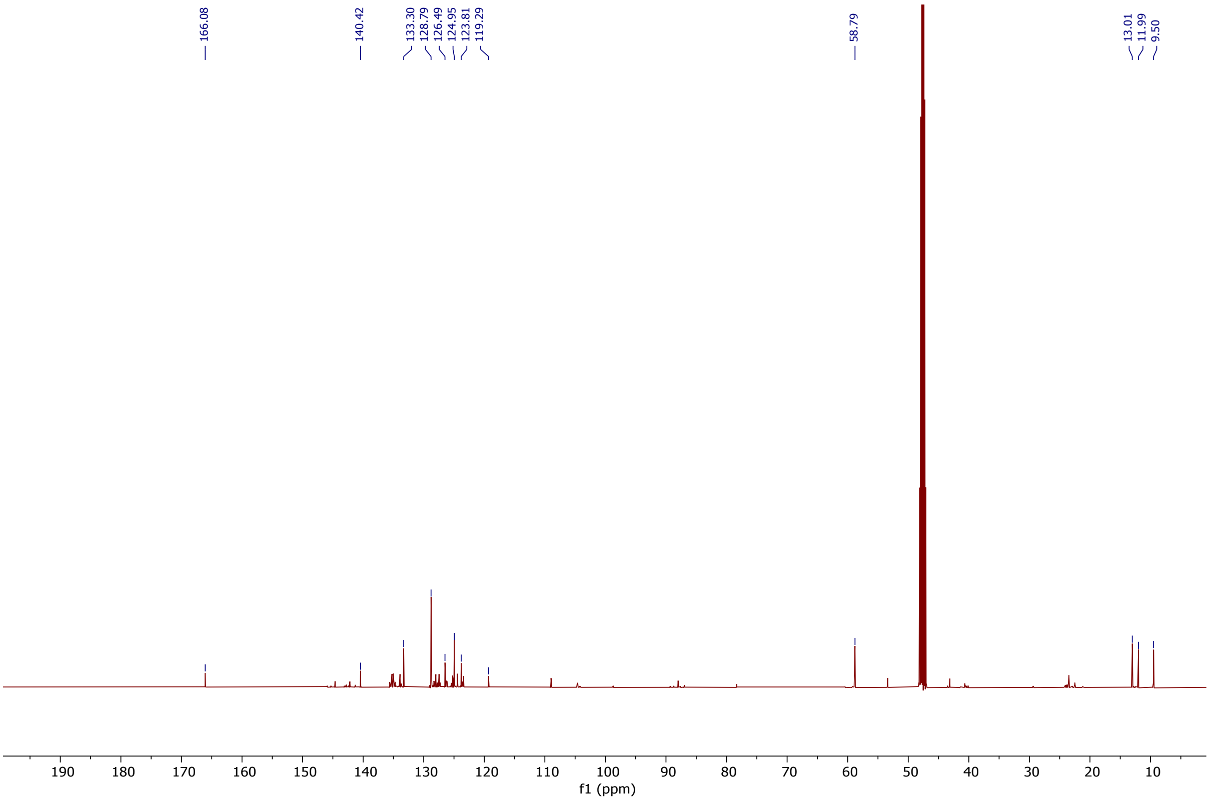

**5d**

1H NMR (500 MHz, MeOD) δ 8.18 (d, *J* = 8.8 Hz, 2H), 7.42 (d, *J* = 8.9 Hz, 2H), 4.06 (q, *J* = 7.1 Hz, 2H), 2.47 (s, 3H), 2.11 (s, 3H), 1.10 (t, *J* = 7.1 Hz, 3H).

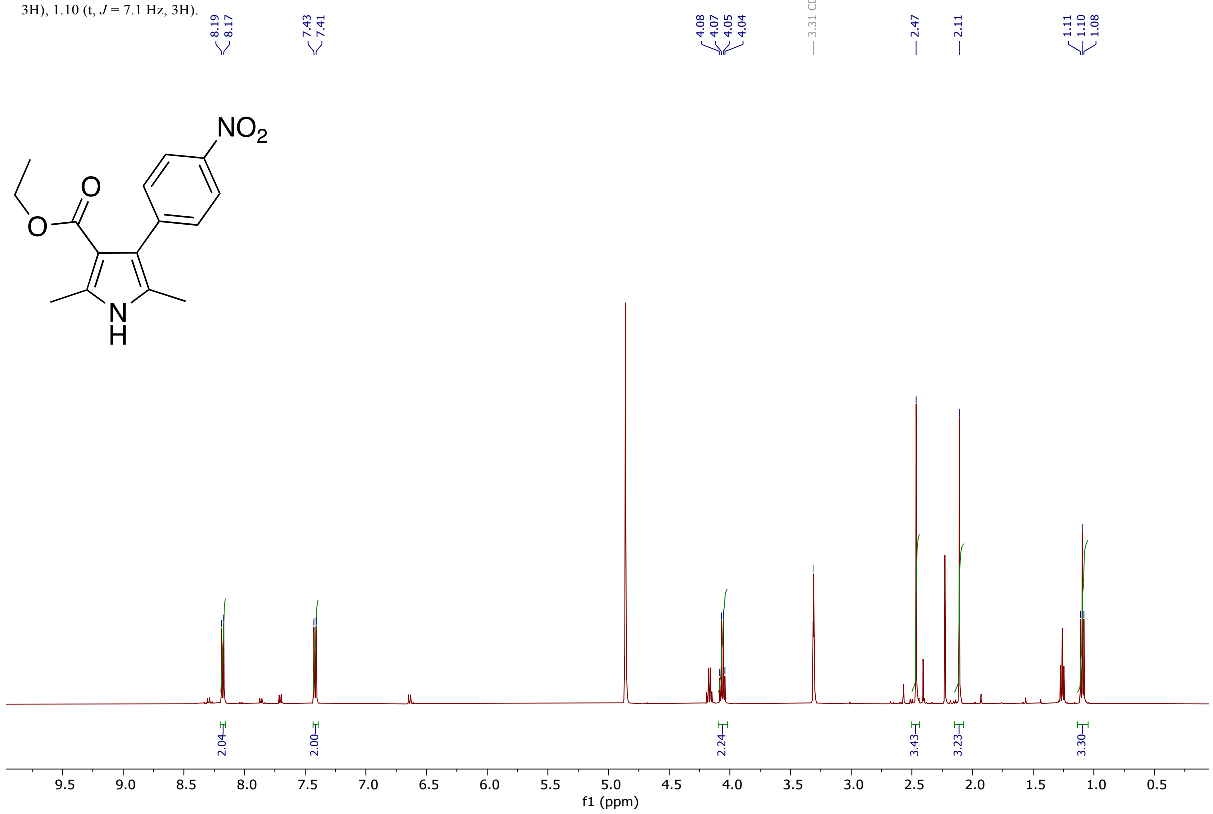


13C NMR (126 MHz, MeOD) δ 167.5, 147.2, 145.8, 136.8, 132.4, 126.3, 123.5, 121.5, 110.5, 60.3, 14.5, 13.5, 11.0.

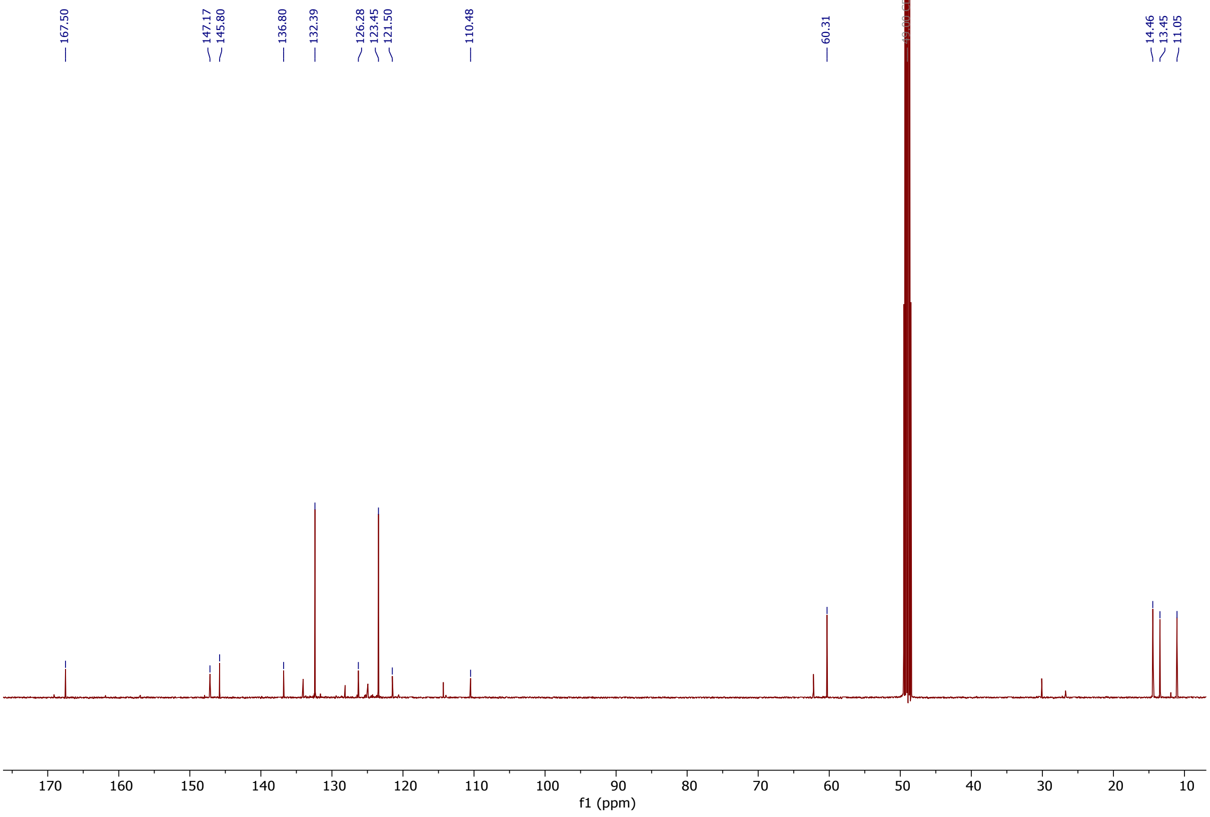

**5e**

$^{1}$H NMR (400 MHz, MeOD) δ 7.40 – 7.25 (m, 4H), 4.04 (q, *J* = 7.1 Hz, 2H), 2.46 (s, 3H), 2.06 (s, 3H), 1.10 (t, *J* = 7.1 Hz, 3H).

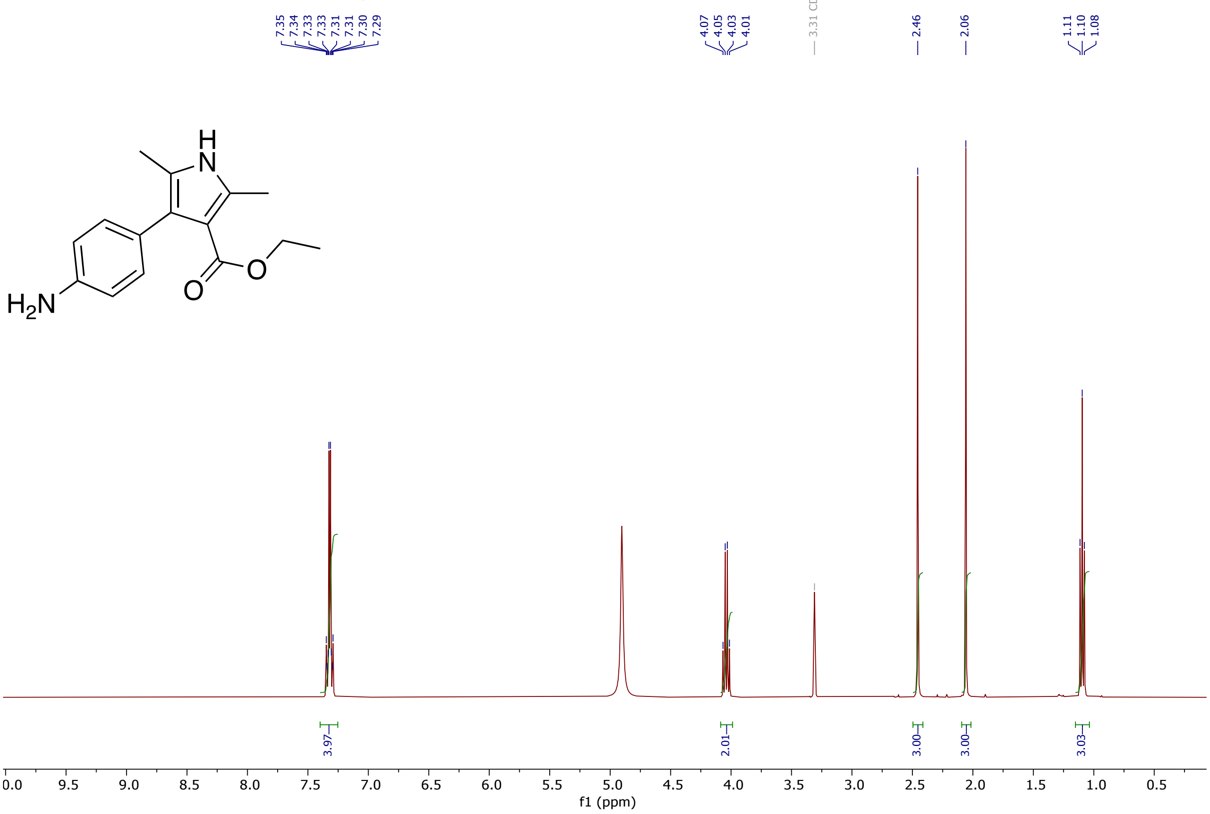


$^{13}$C NMR (101 MHz, MeOD) δ 167.75, 139.12, 136.27, 133.23, 130.06, 125.50, 122.59, 121.87, 110.47, 60.14, 14.50, 13.54, 10.88.

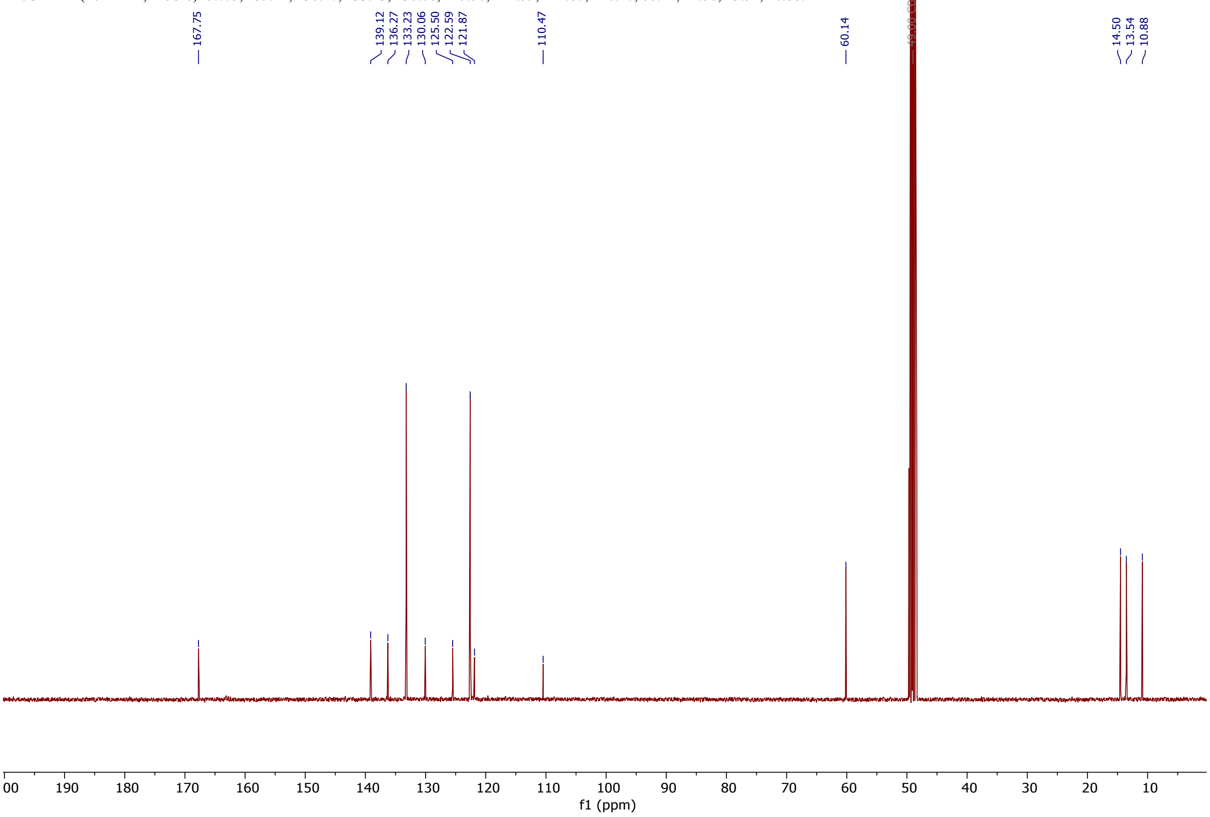


**5f**

1H NMR (500 MHz, MeOD)δ 4.20 (q, $J$ = 7.1 Hz, 2H), 2.59 (d, $J$ = 7.4 Hz, 2H), 2.38 (s, 3H), 2.07 (s, 3H), 1.33 (t, $J$ = 7.1 Hz, 3H), 1.04 (t, $J$ = 7.4 Hz, 3H).

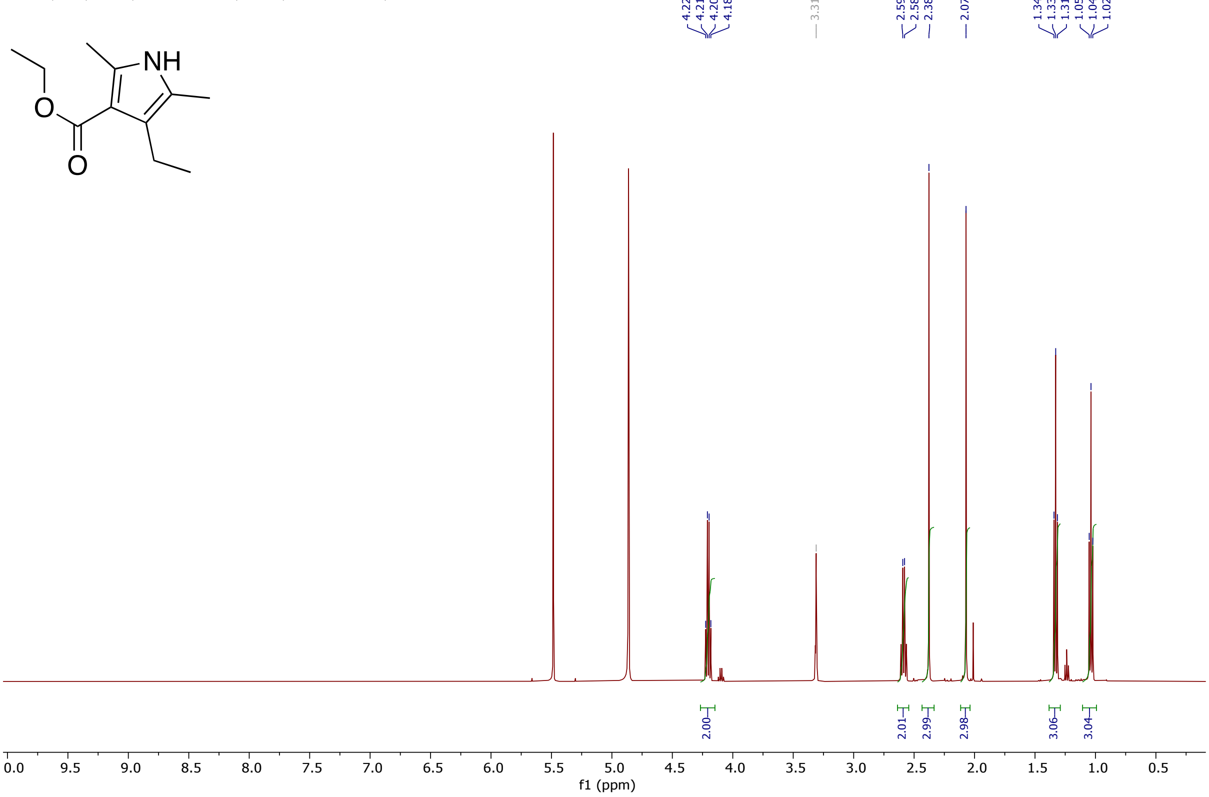


13C NMR (126 MHz, MeOD)δ 168.5, 135.5, 123.5, 123.0, 109.8, 60.0, 19.4, 16.5, 14.8, 13.8, 10.1.

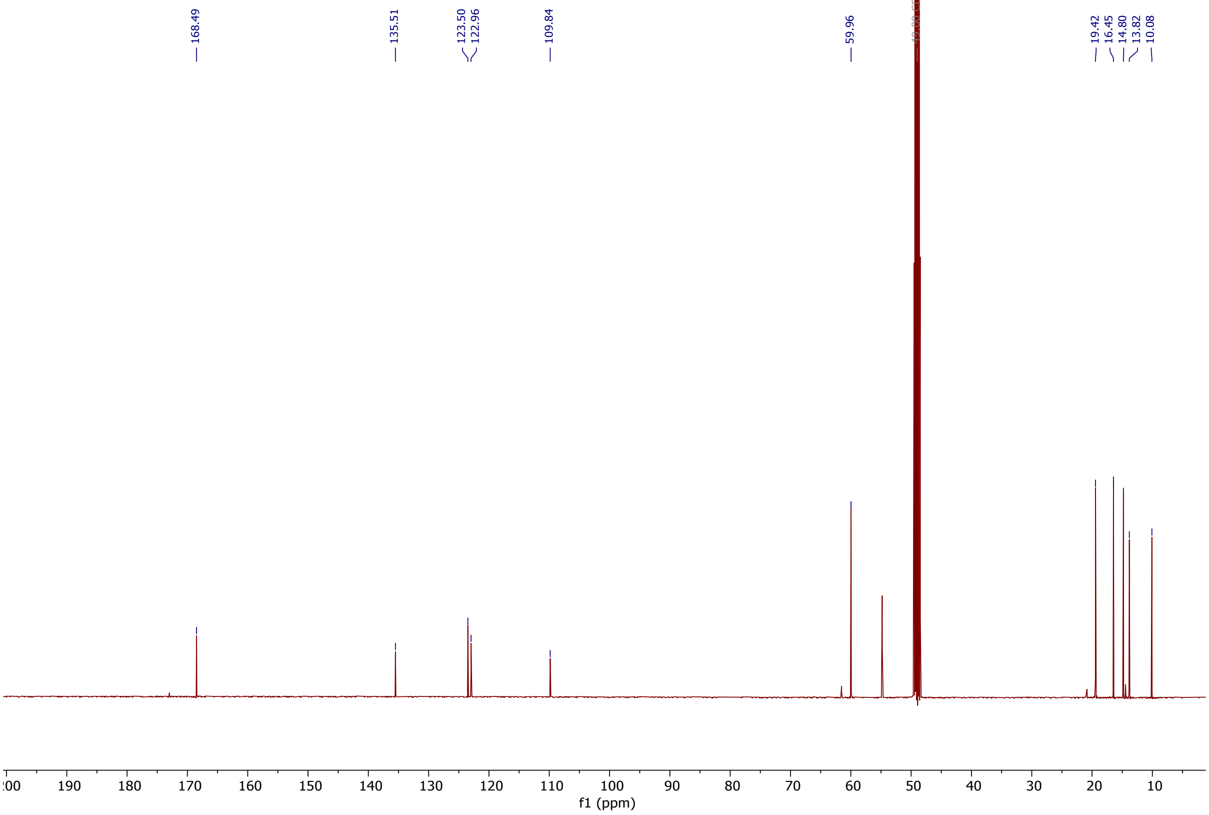

**5g**

$^{1}$H NMR (500 MHz, MeOD) δ 7.31 – 7.24 (m, 2H), 7.21 – 7.14 (m, 3H), 3.55 (s, 3H), 2.45 (s, 3H), 2.05 (s, 3H).

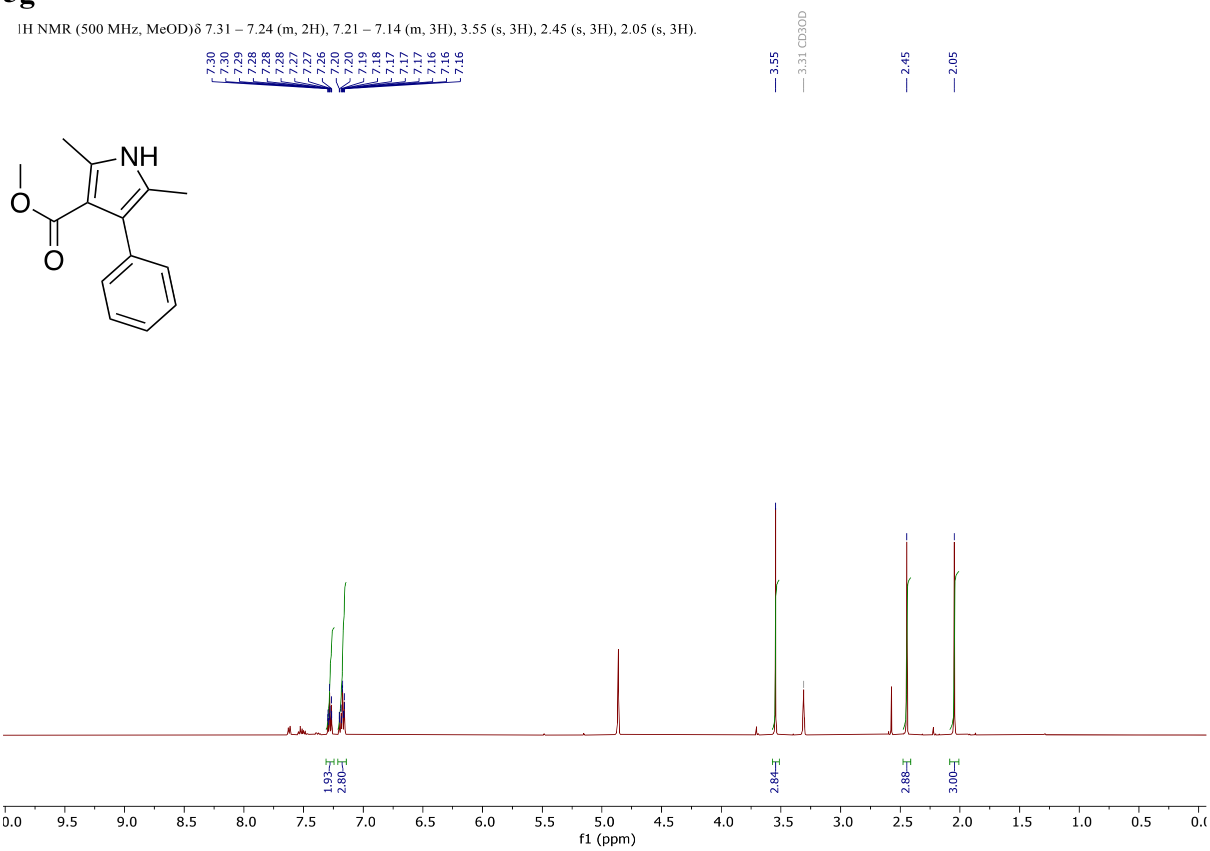


$^{13}$C NMR (126 MHz, MeOD) δ 168.5, 138.1, 135.8, 131.5, 128.3, 126.6, 125.1, 123.5, 110.4, 50.6, 13.5, 11.0.

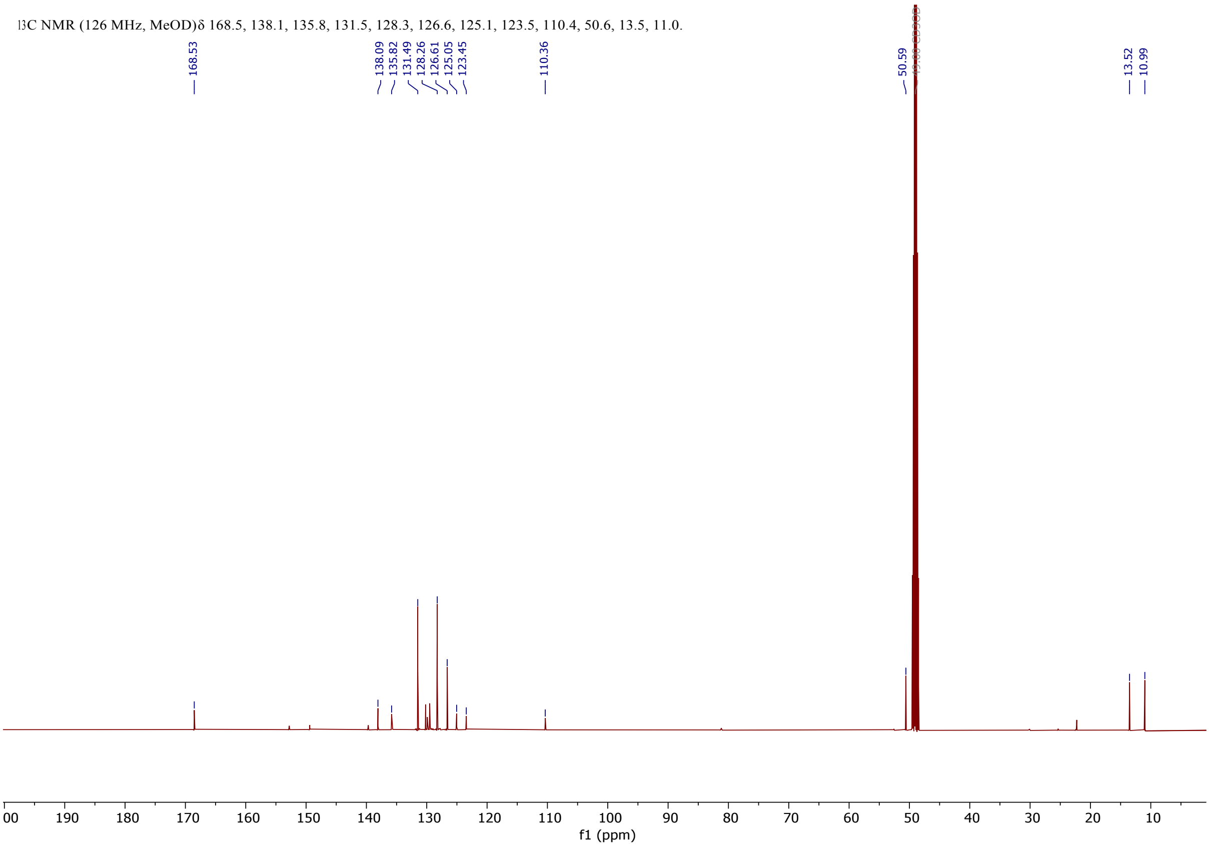

## 5h

1H NMR (500 MHz, MeOD)δ 7.51 – 7.40 (m, 4H), 3.55 (s, 3H), 2.46 (s, 3H), 2.07 (s, 3H).

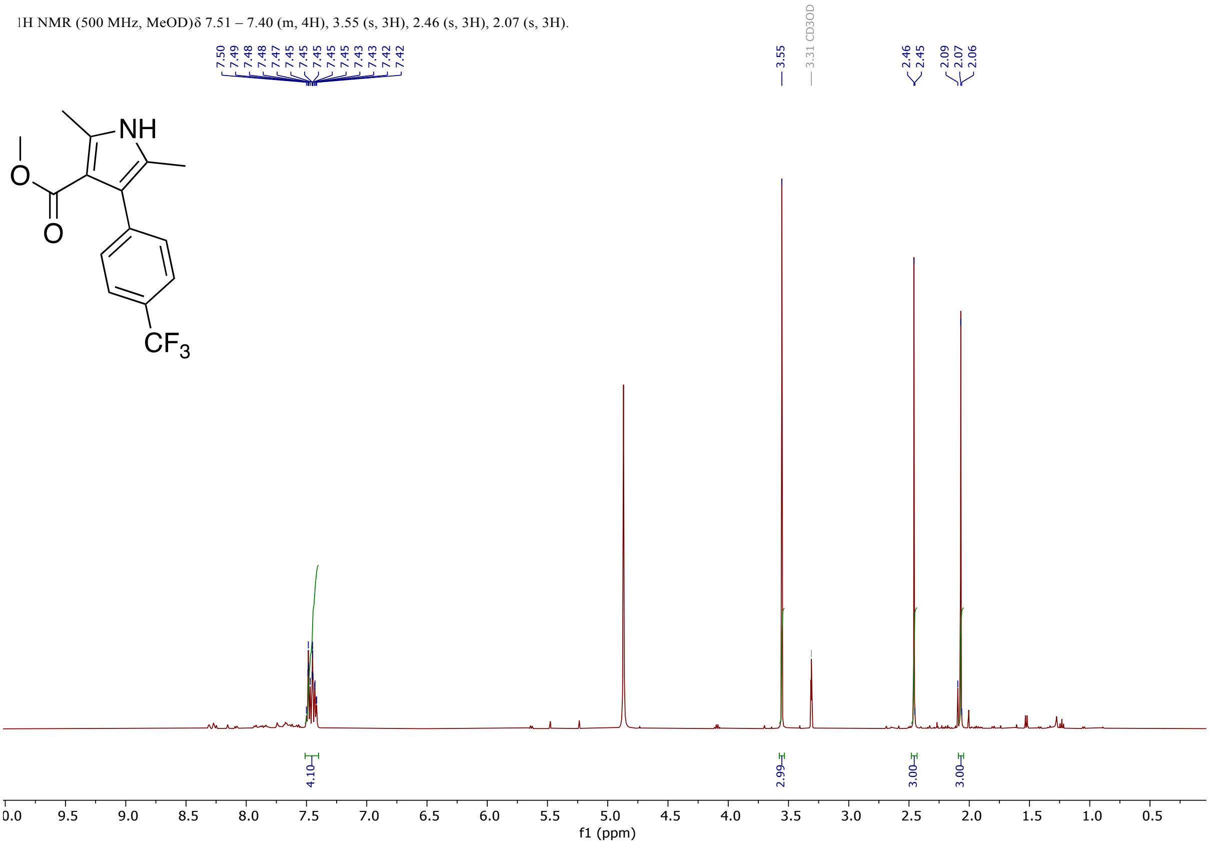


13C NMR (126 MHz, MeOD)δ 168.1, 139.1, 136.5, 135.1, 129.0, 128.2 (q, *J* = 3.9 Hz), 125.7, 123.2, 121.9, 110.2, 50.6, 13.5, 10.9.

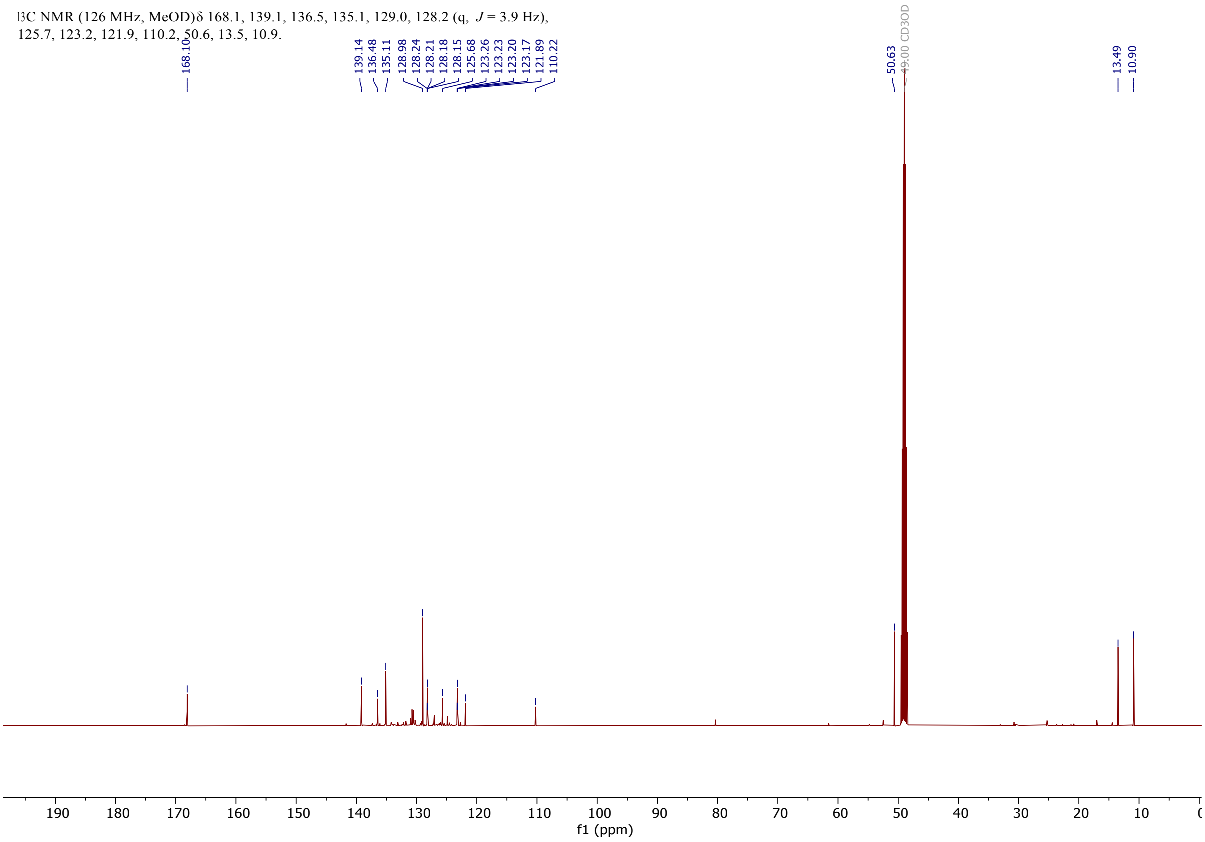

# 5i

1H NMR (500 MHz, MeOD) δ 7.27 (t, *J* = 1.9 Hz, 1H), 7.12 (d, *J* = 2.0 Hz, 2H), 3.61 (s, 3H), 2.44 (s, 3H), 2.08 (s, 3H).

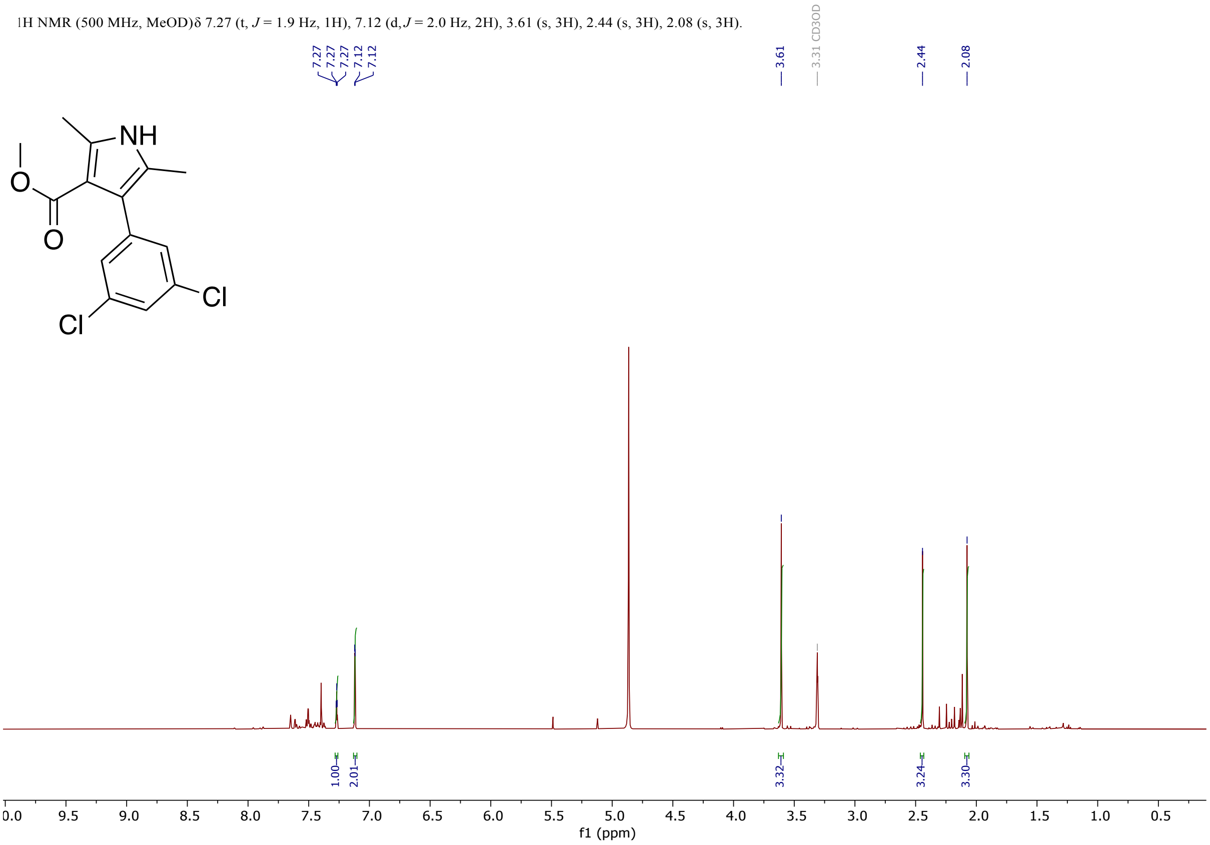


13C NMR (126 MHz, MeOD) δ 167.9, 141.7, 134.6, 130.0, 126.5, 126.4, 125.0, 120.8, 110.2, 50.8, 13.5, 10.9.

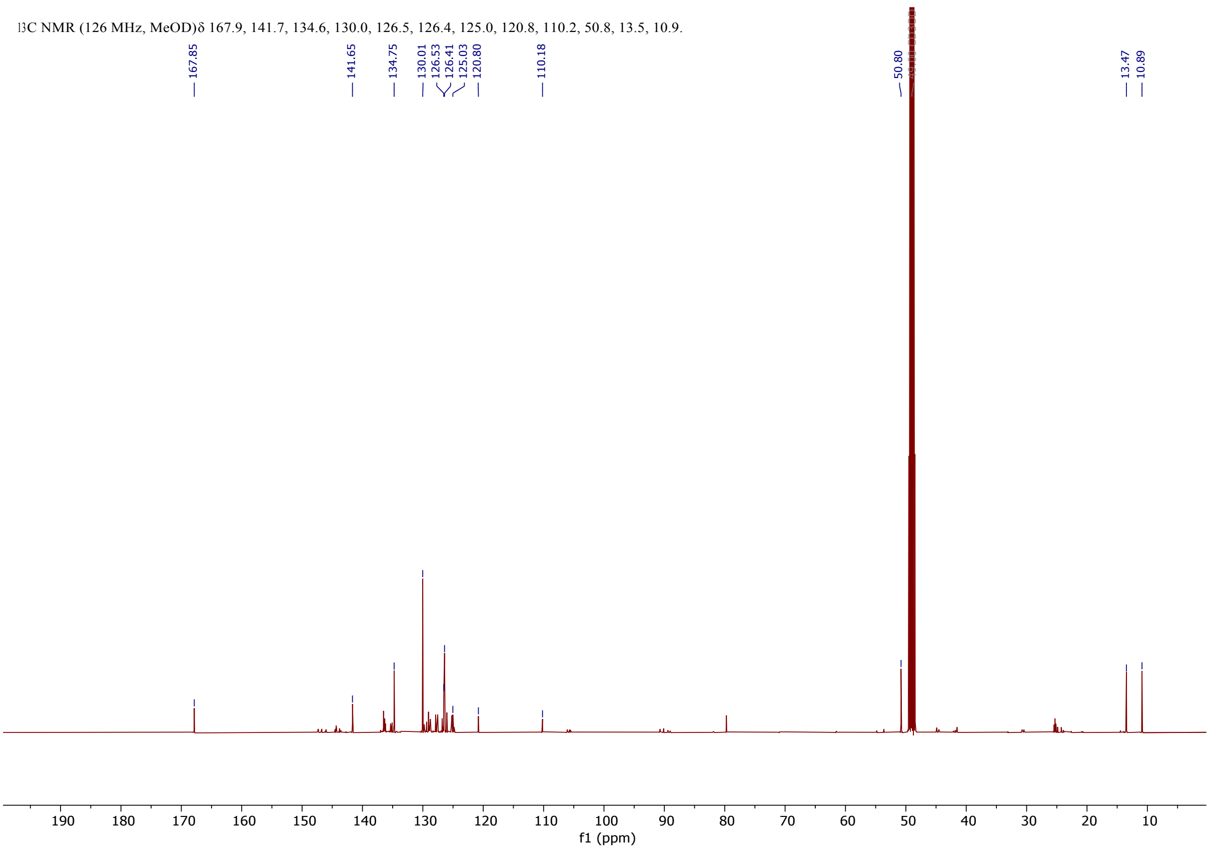

**5j**

1H NMR (500 MHz, MeOD) δ 8.17 (d, *J* = 8.8 Hz, 2H), 7.40 (d, *J* = 8.8 Hz, 2H), 3.59 (s, 3H), 2.46 (s, 3H), 2.11 (s, 3H).

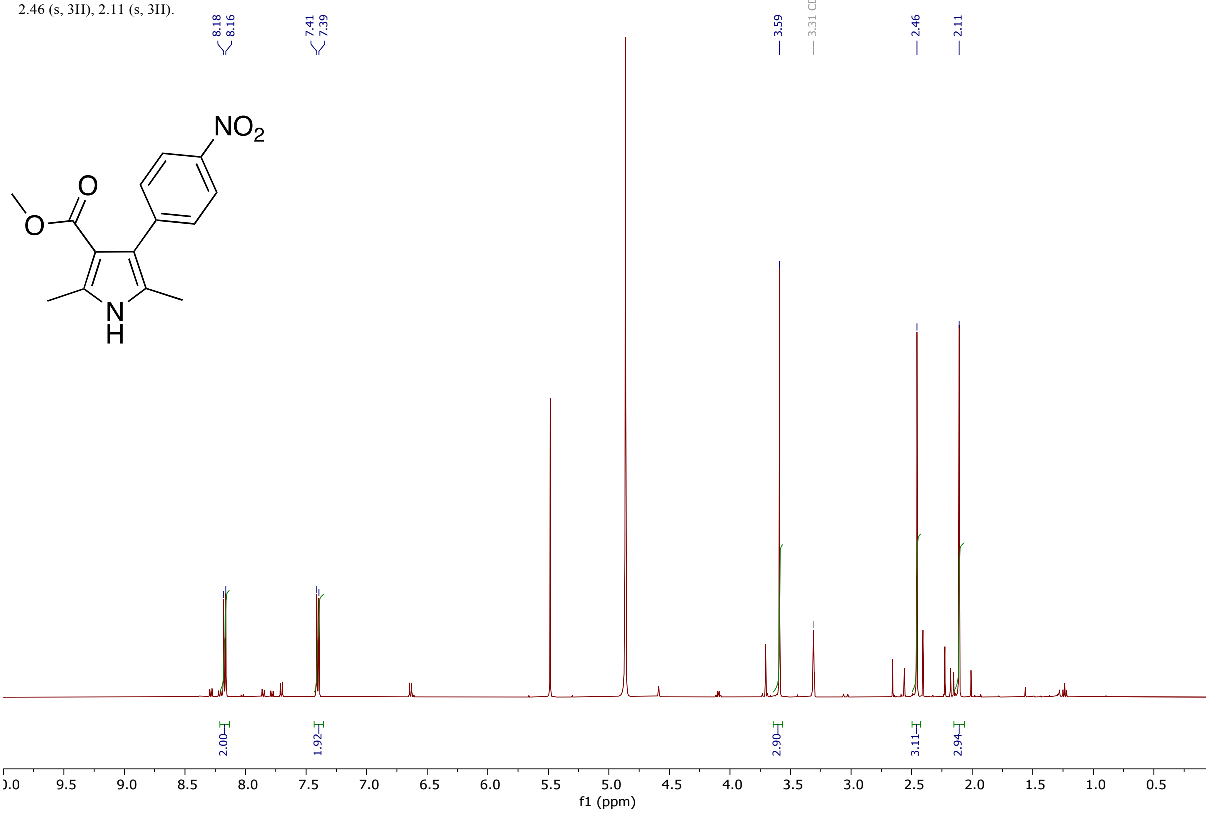


13C NMR (126 MHz, MeOD) δ 167.9, 147.1, 145.6, 136.9, 132.2, 126.4, 123.5, 121.5, 110.3, 50.8, 13.5, 11.1.

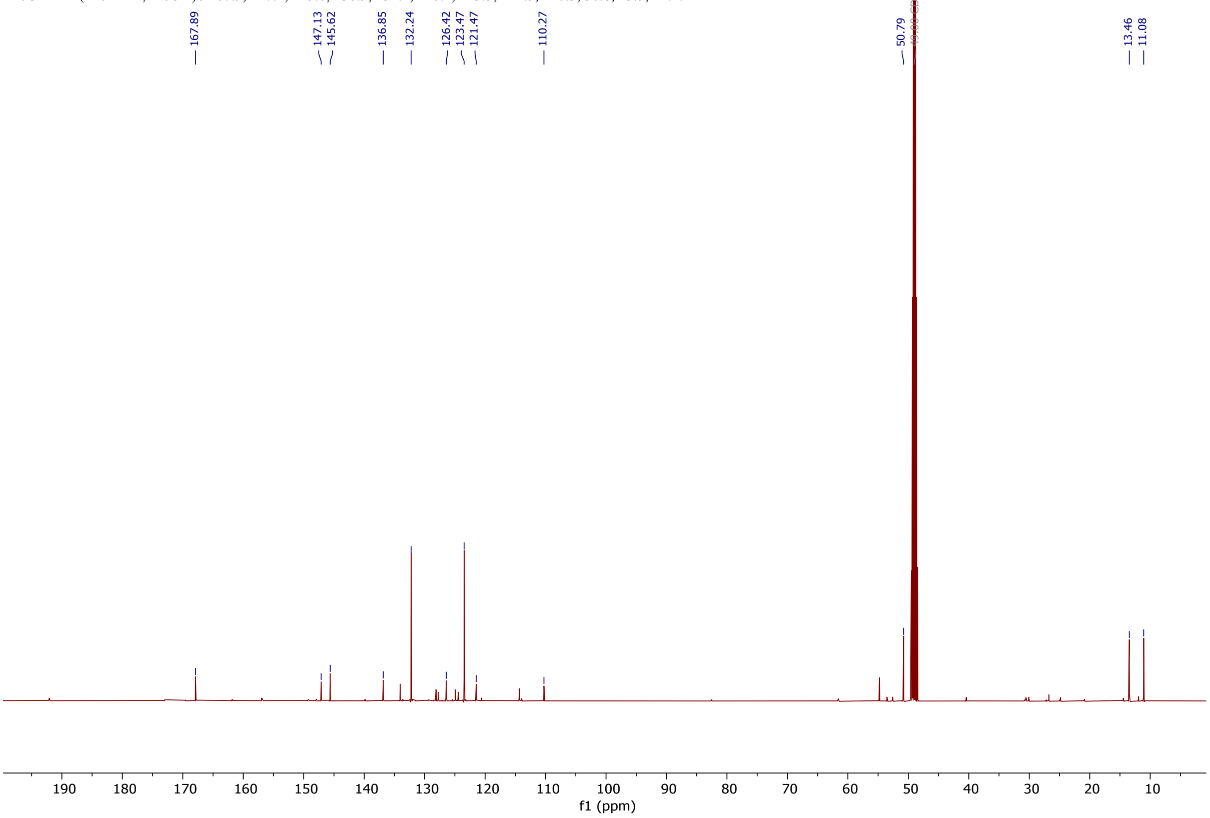

**5k**

1H NMR (400 MHz, MeOD) δ 7.36 – 7.27 (m, 4H), 3.58 (s, 3H), 2.45 (s, 3H), 2.06 (s, 3H).

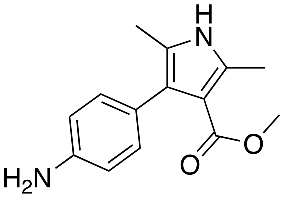

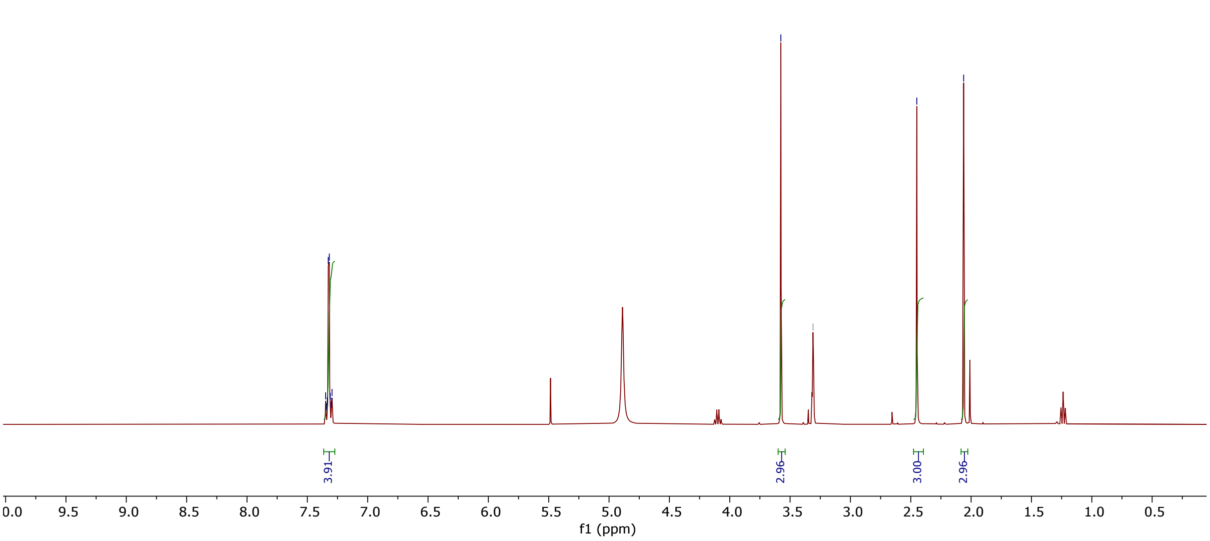


13C NMR (101 MHz, MeOD) δ 168.11, 139.08, 136.31, 133.14, 129.87, 125.63, 122.68, 121.87, 110.24, 50.68, 13.56, 10.89.

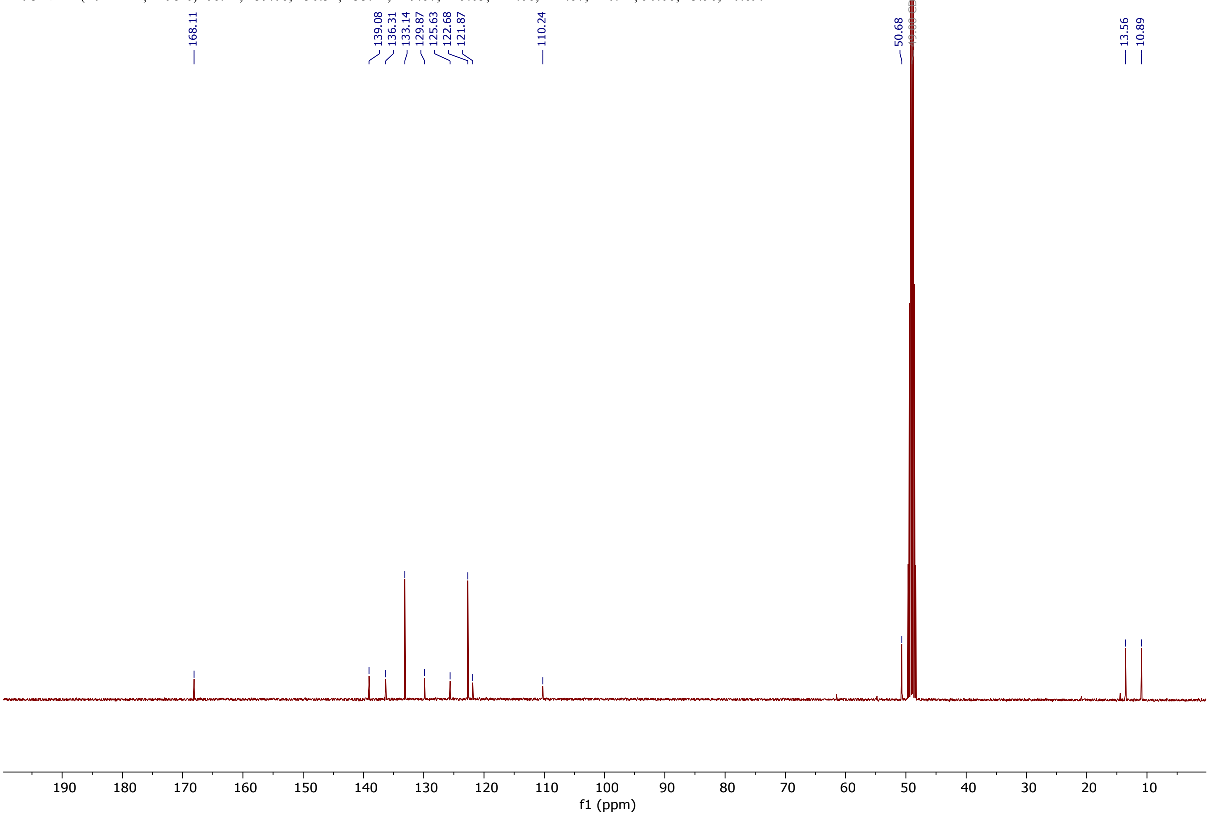


**5l**

1H NMR (500 MHz, MeOD)δ 3.74 (s, 3H), 2.58 (q, *J* = 7.4 Hz, 2H), 2.37 (s, 3H), 2.07 (s, 3H), 1.03 (t, *J* = 7.4 Hz, 3H).

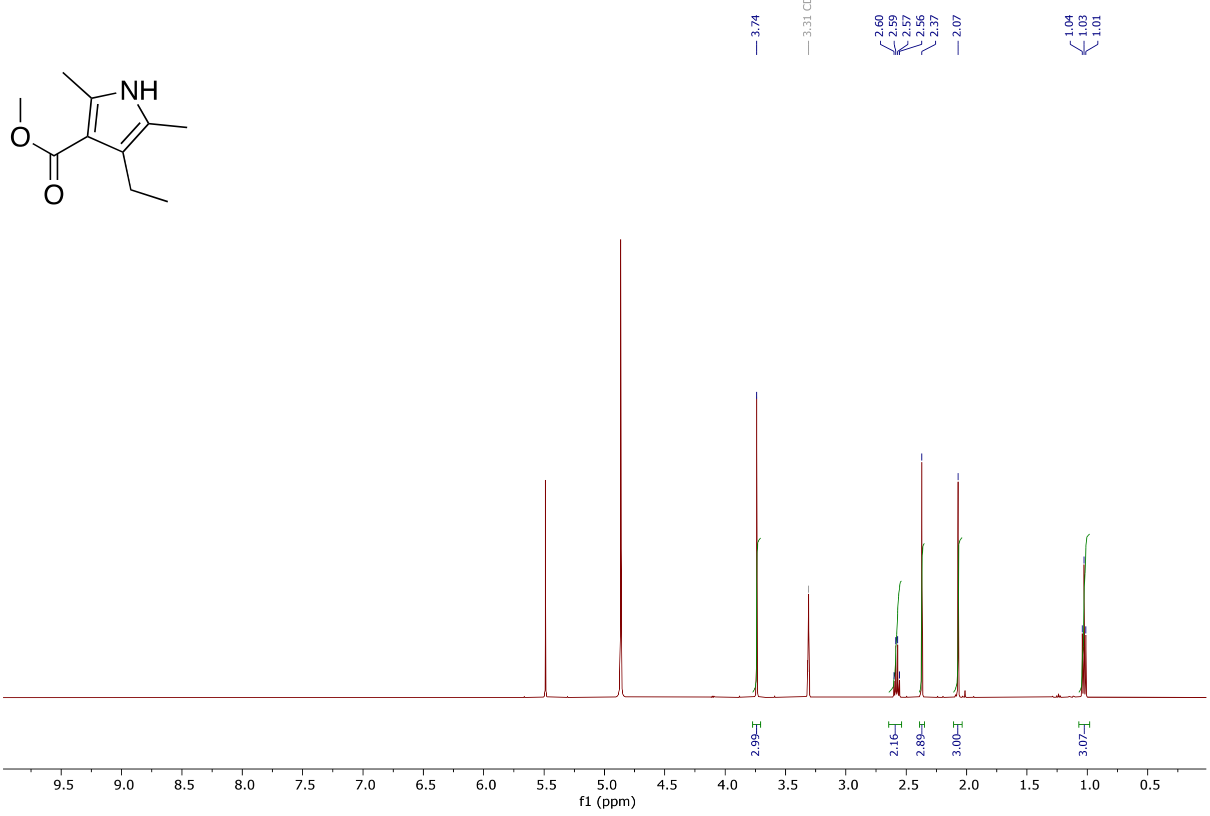


13C NMR (126 MHz, MeOD)δ 168.9, 135.5, 123.6, 123.0, 109.7, 50.6, 19.4, 16.4, 13.8, 10.1.

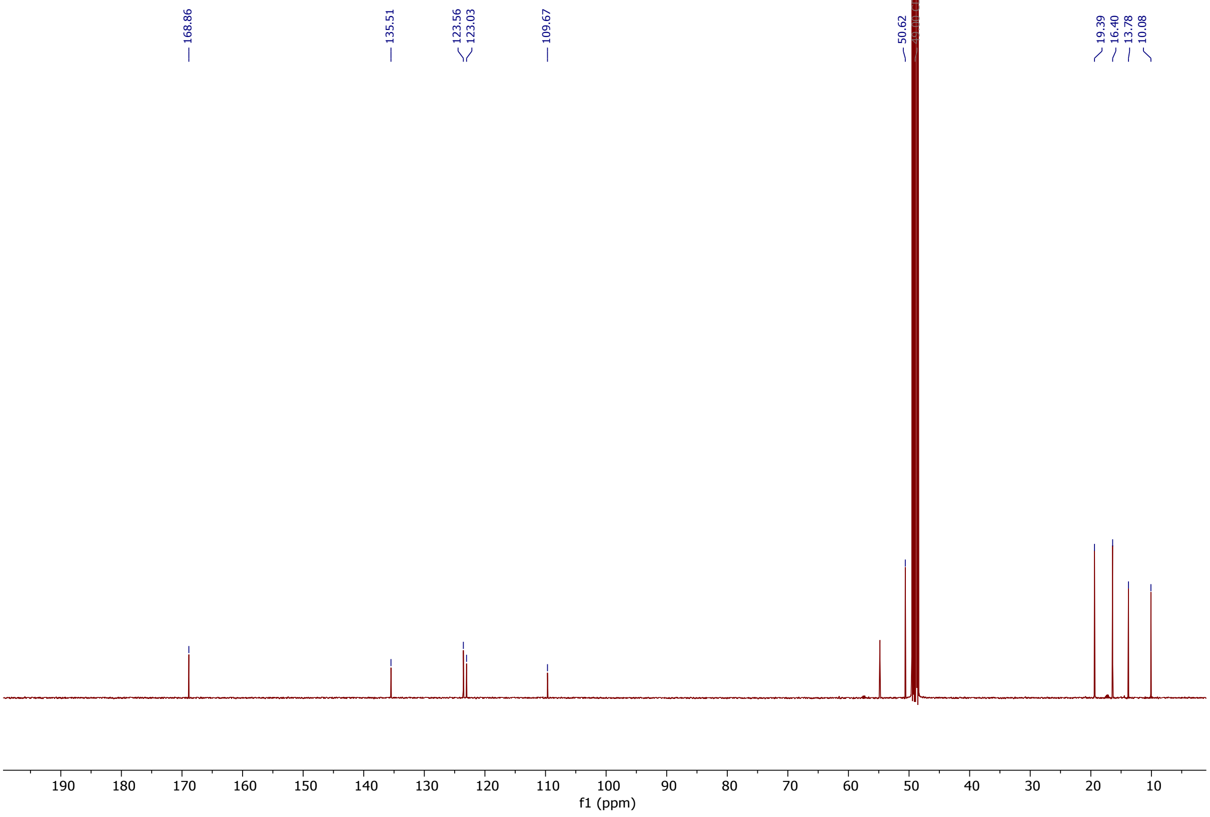

## I-1

1H NMR (400 MHz, $CDCl_3$) δ 8.20 – 8.13 (m, 2H), 7.68 – 7.60 (m, 1H), 7.57 – 7.48 (m, 1H), 3.79 (s, 2H).

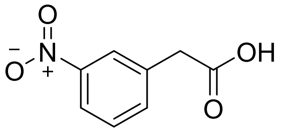


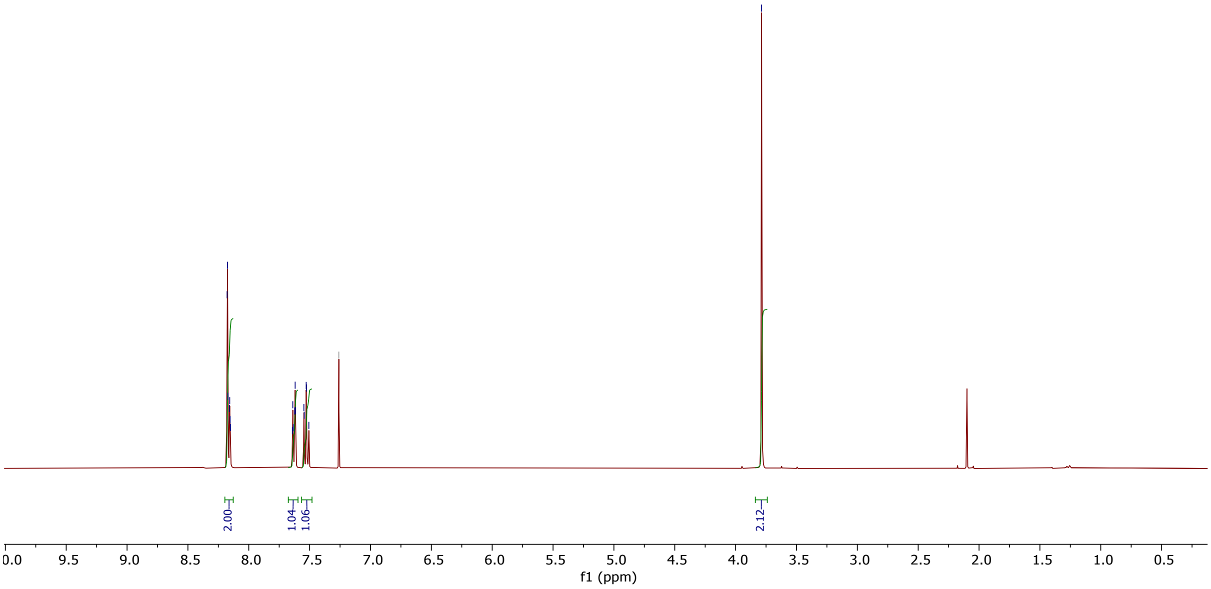


13C NMR (101 MHz, $CDCl_3$) δ 176.2, 148.5, 135.8, 135.1, 129.7, 124.7, 122.7, 40.4.

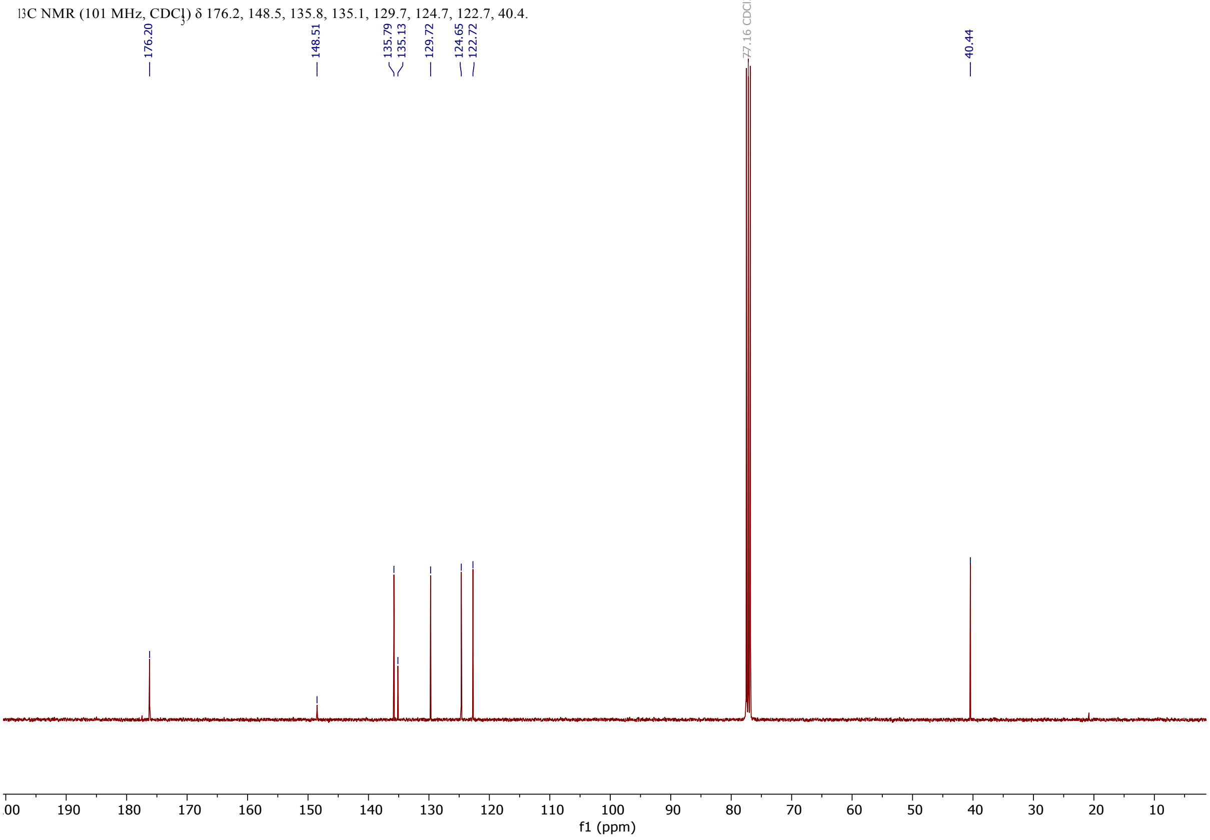

**I-2**

$^1$H NMR (400 MHz, $CDCl_3$) δ 8.19 – 8.08 (m, 2H), 7.68 – 7.61 (m, 1H), 7.49 (t, $J$ = 7.9 Hz, 1H), 3.87 (s, 2H), 3.71 (s, 3H), 3.21 (s, 3H).

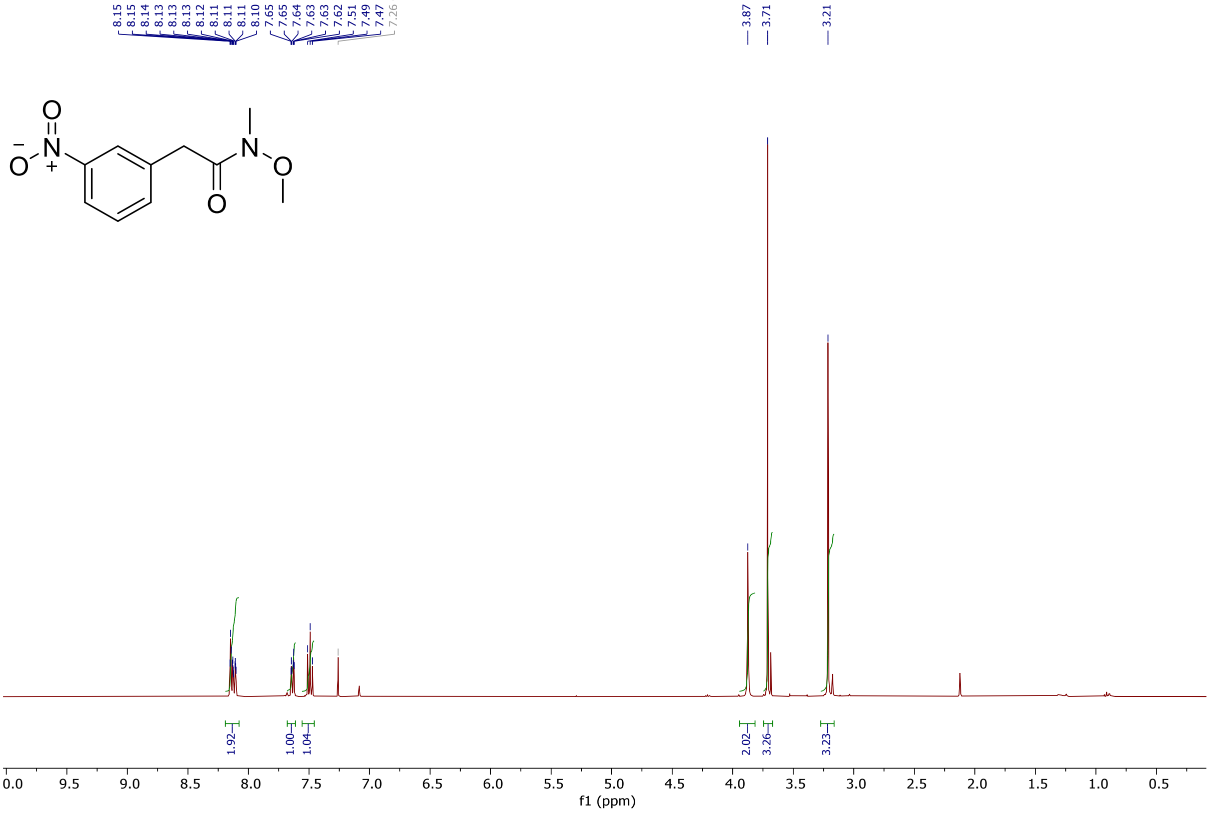


$^{13}$C NMR (101 MHz, $CDCl_3$) δ 207.1, 148.4, 136.9, 136.0, 129.5, 124.7, 122.2, 61.6, 38.8, 31.1.

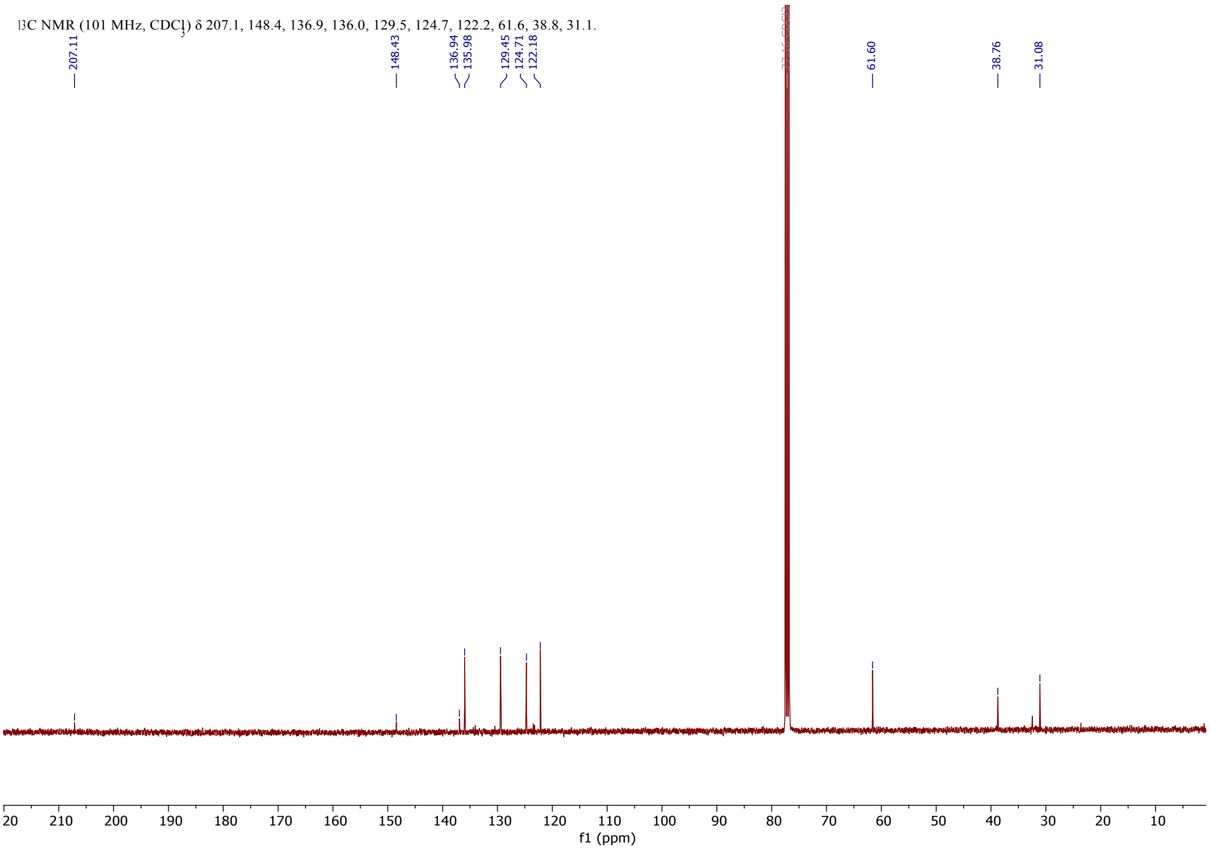


**I-3**

$^1H$ NMR (400 MHz, $CDCl_3$) δ 7.09 (t, $J$ = 8.0 Hz, 1H), 6.73 – 6.66 (m, 2H), 6.61 – 6.55 (m, 1H), 3.68 (s, 2H), 3.60 (s, 3H), 3.19 (s, 3H).

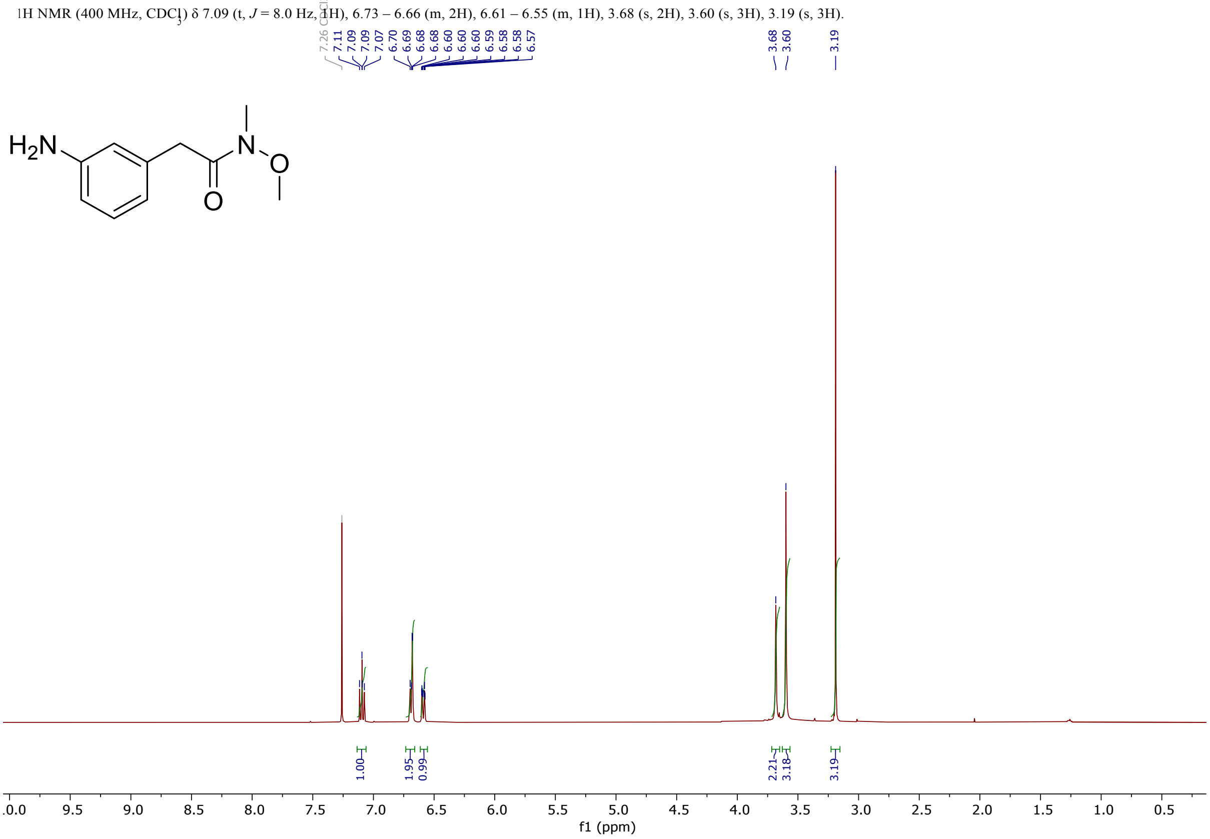


$^{13}C$ NMR (101 MHz, $CDCl_3$) δ 172.6, 146.3, 136.2, 129.6, 120.0, 116.2, 114.0, 61.5, 39.5, 32.4.

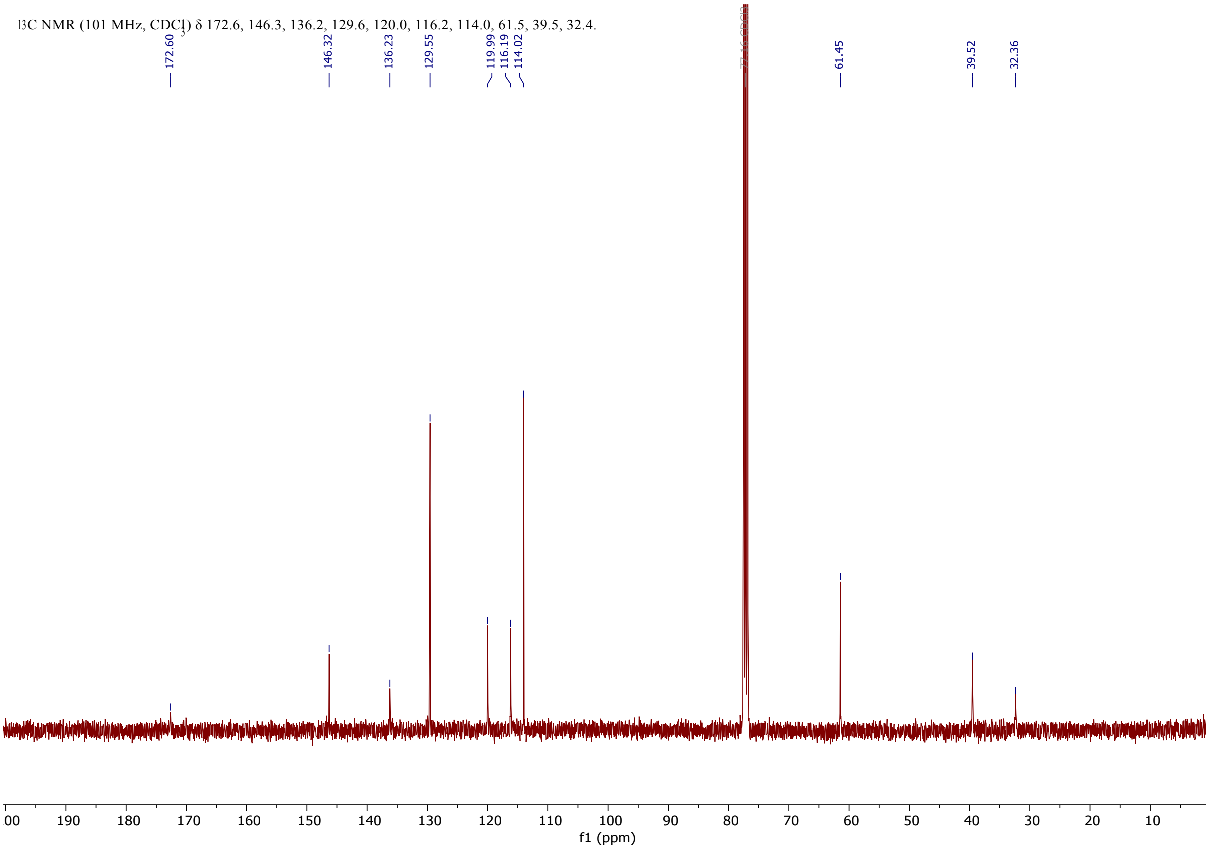

**I-4**

1H NMR (400 MHz, CDCl$_3$) δ 7.17 (t, *J* = 7.9 Hz, 1H), 6.73 – 6.59 (m, 3H), 3.73 (s, 2H), 3.59 (s, 3H), 3.19 (s, 3H), 2.94 (s, 6H).

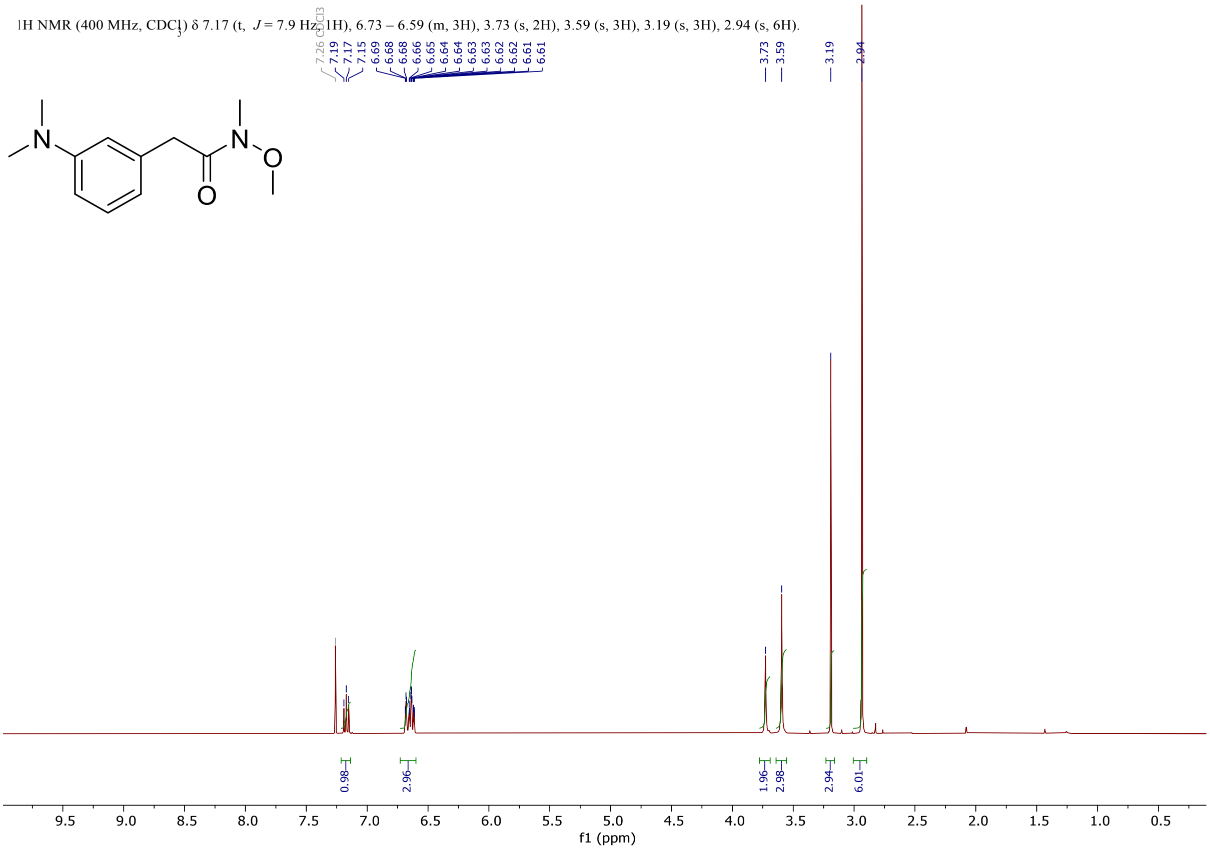


13C NMR (101 MHz, CDCl$_3$) δ 172.9, 150.9, 135.8, 129.2, 117.7, 113.5, 111.3, 61.4, 40.7, 40.0, 32.4.

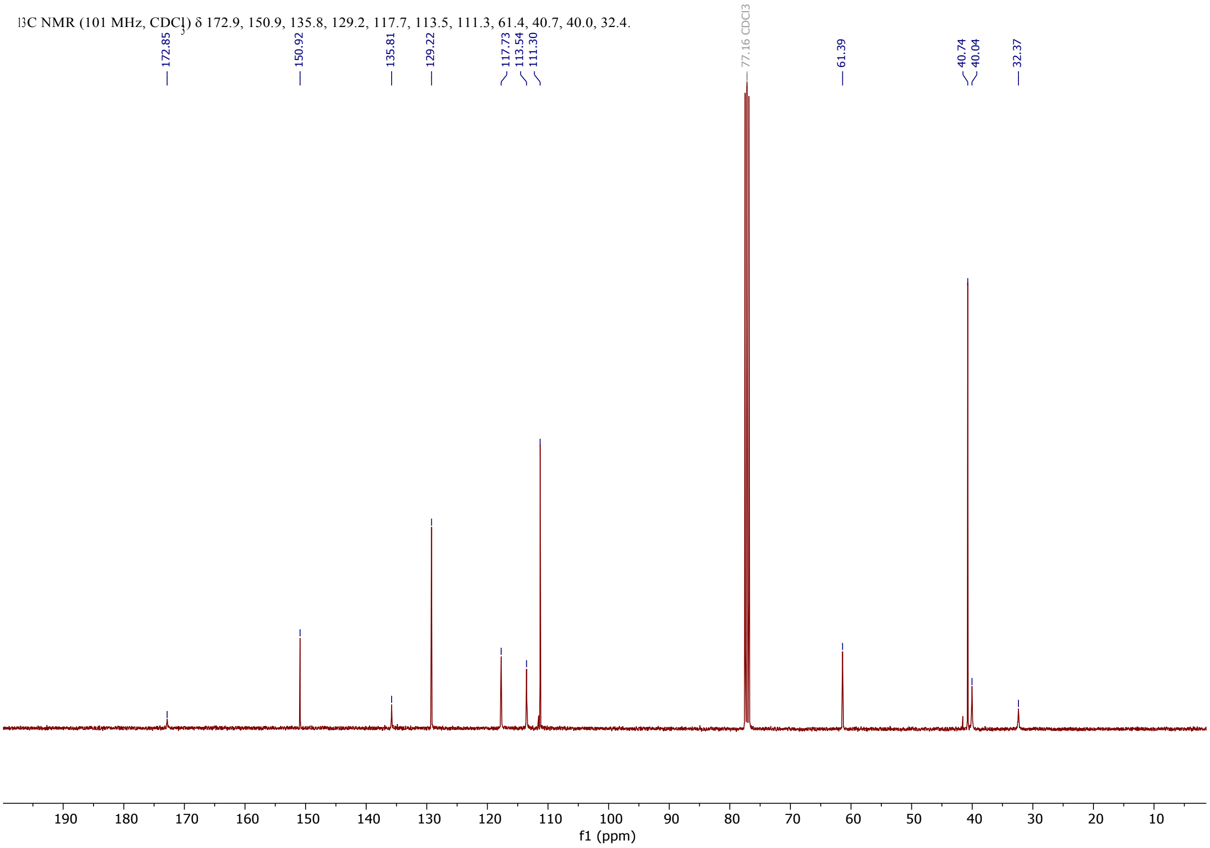

**8a**

$^1$H NMR (500 MHz, cdcl$_3$) δ 7.20 (t, $J$ = 7.8 1H), 6.68 – 6.62 (m, 1H), 6.60 – 6.52 (m, 2H), 3.63 (s, 2H), 2.94 (s, 6H), 2.14 (s, 3H).

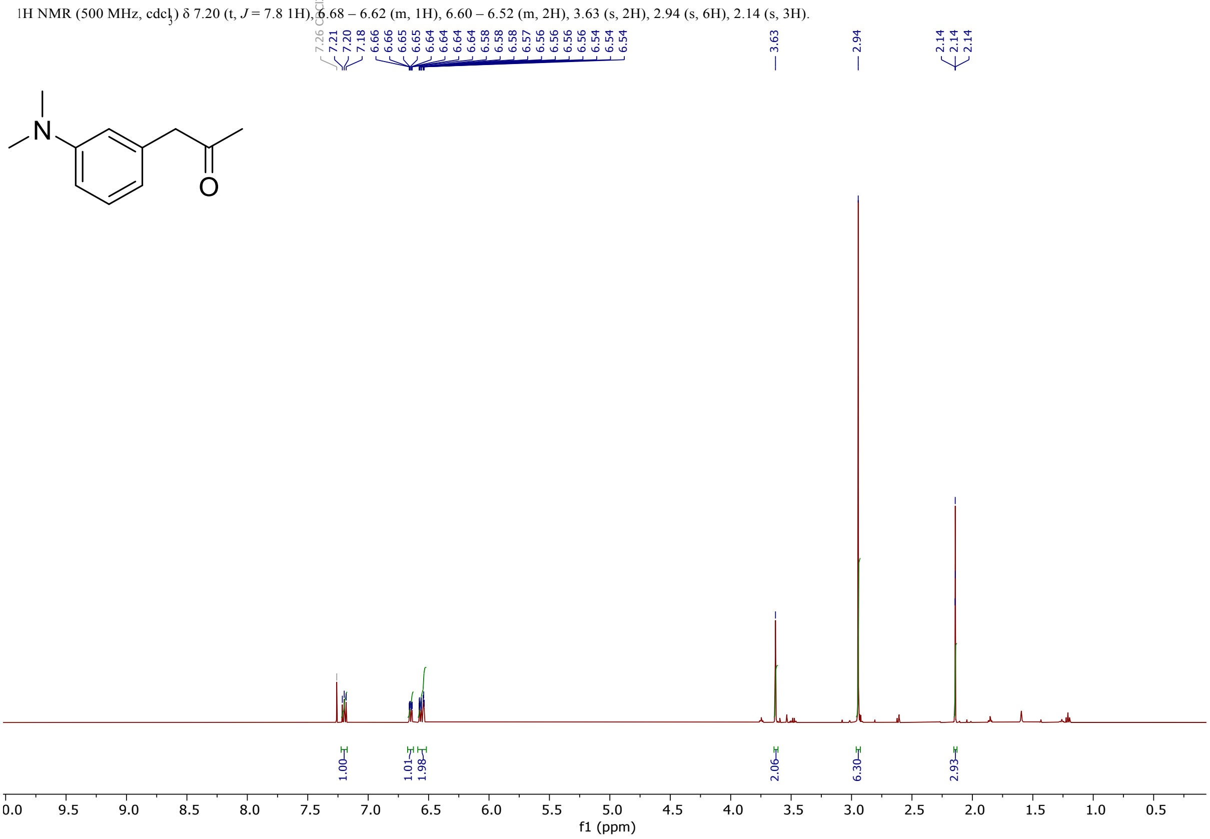


$^{13}$C NMR (126 MHz, cdcl$_3$) δ 207.2, 151.1, 135.3, 129.6, 117.7, 113.4, 111.4, 51.9, 40.7, 29.2.

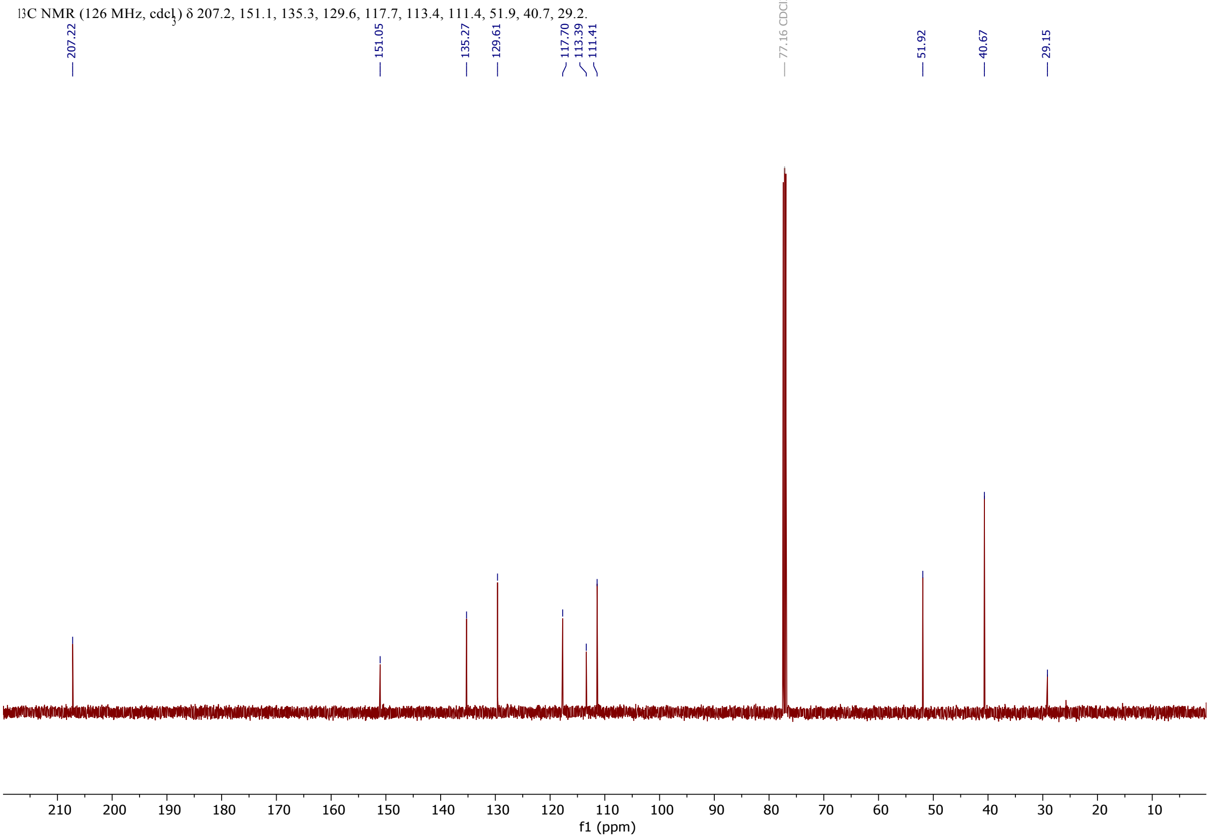


**9a**

$^1$H NMR (400 MHz, $CDCl_3$) δ 7.17 (t, *J* = 8.0 Hz, 1H), 6.63 – 6.50 (m, 3H), 3.34 – 3.26 (m, 1H), 3.14 (s, 2H), 2.94 (s, 6H), 2.78 – 2.63 (m, 2H), 1.22 (d, *J* = 6.3 Hz, 3H).

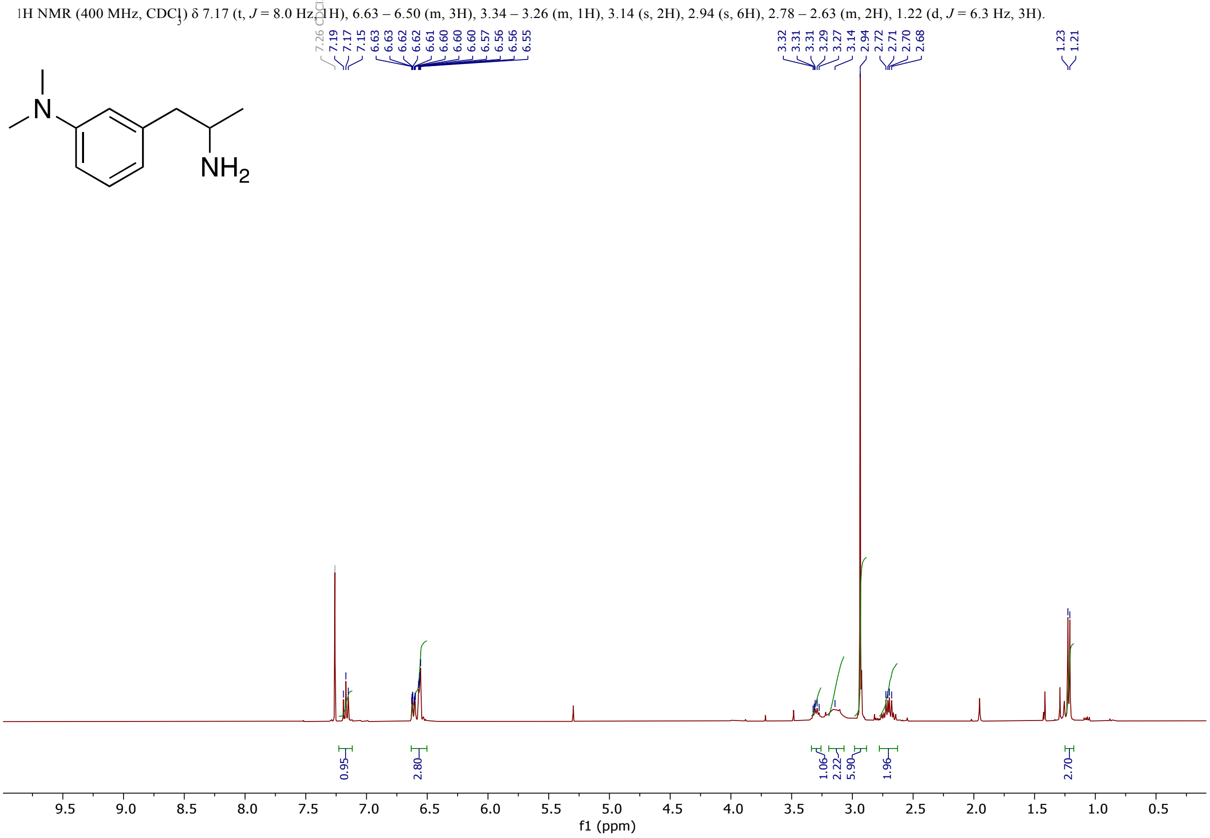


$^{13}$C NMR (101 MHz, $CDCl_3$) δ 150.8, 139.3, 129.2, 117.5, 113.5, 110.8, 48.7, 45.4, 40.6, 21.9.

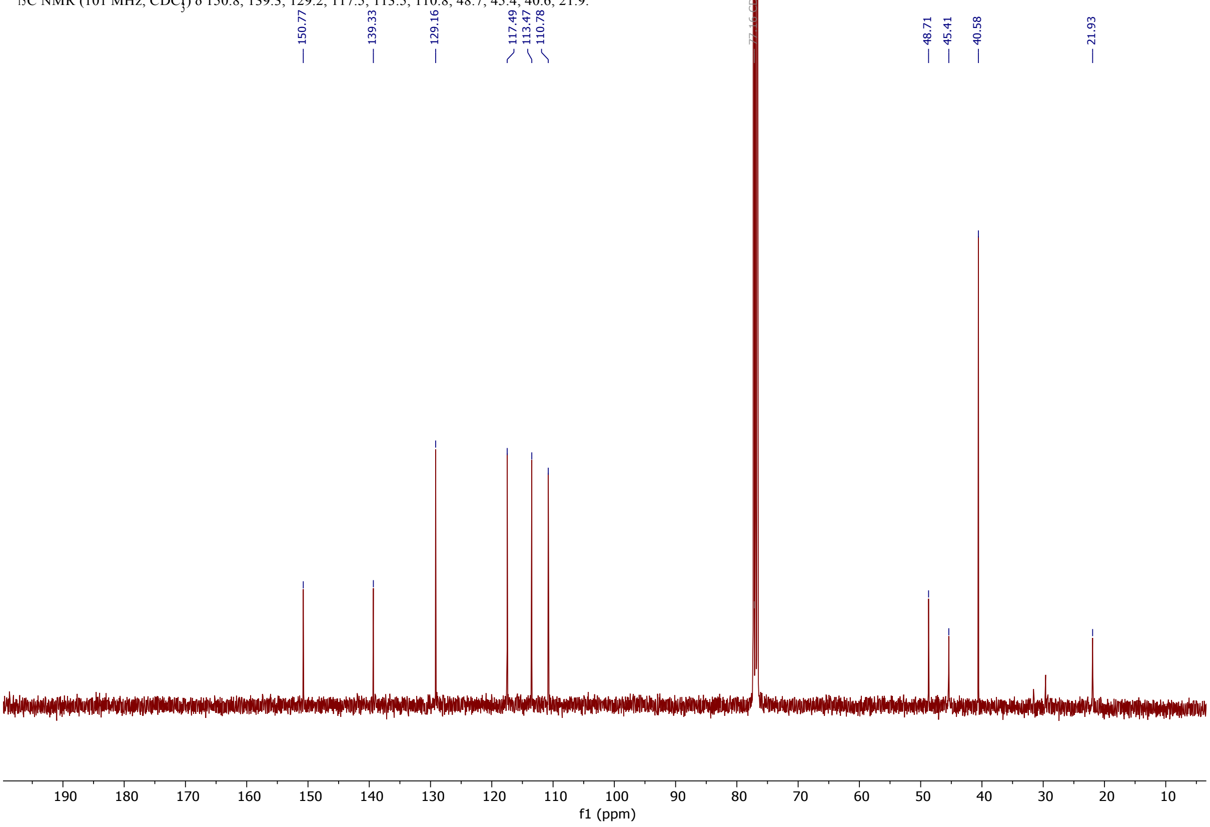

**8c**

$^{1}$H NMR (400 MHz, $CDCl_3$) δ 7.20 (t, *J* = 7.8 Hz, 1H), 6.79 – 6.73 (m, 2H), 6.73 – 6.67 (m, 1H), 3.65 (s, 2H), 2.16 (s, 3H).

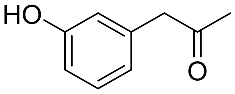

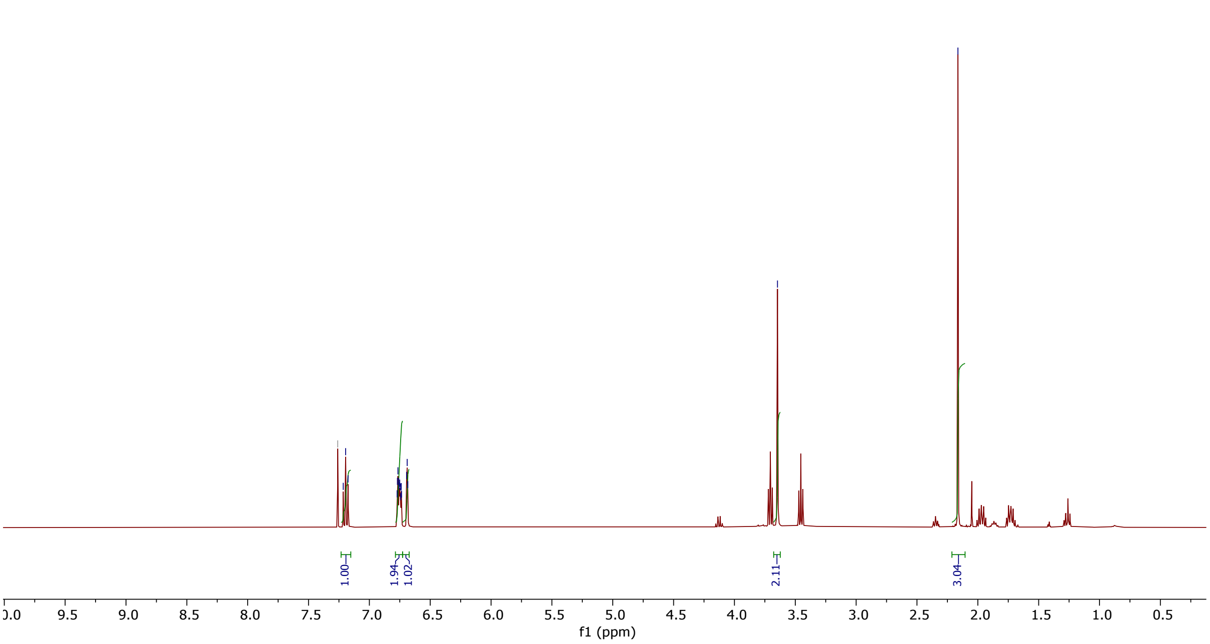


$^{13}$C NMR (126 MHz, $CDCl_3$) δ 207.6, 156.3, 135.7, 130.2, 121.7, 116.4, 114.5, 51.0, 29.4.

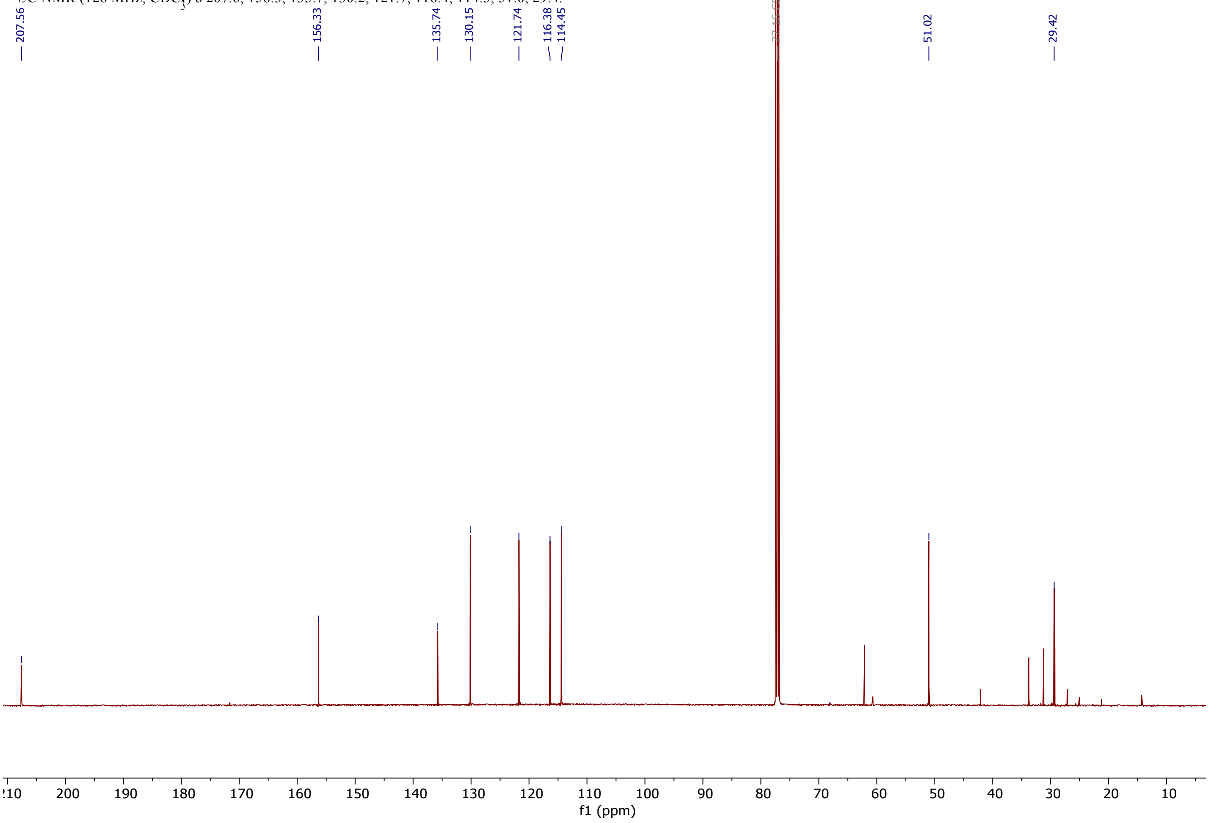


**9c**

1H NMR (400 MHz, MeOD)δ 7.11 (t, J = 8.0 Hz, 1H), 6.71 – 6.58 (m, 3H), 3.16 – 3.03 (m, 1H), 2.63 – 2.48 (m, 2H), 1.08 (d, *J* = 6.4 Hz, 3H).

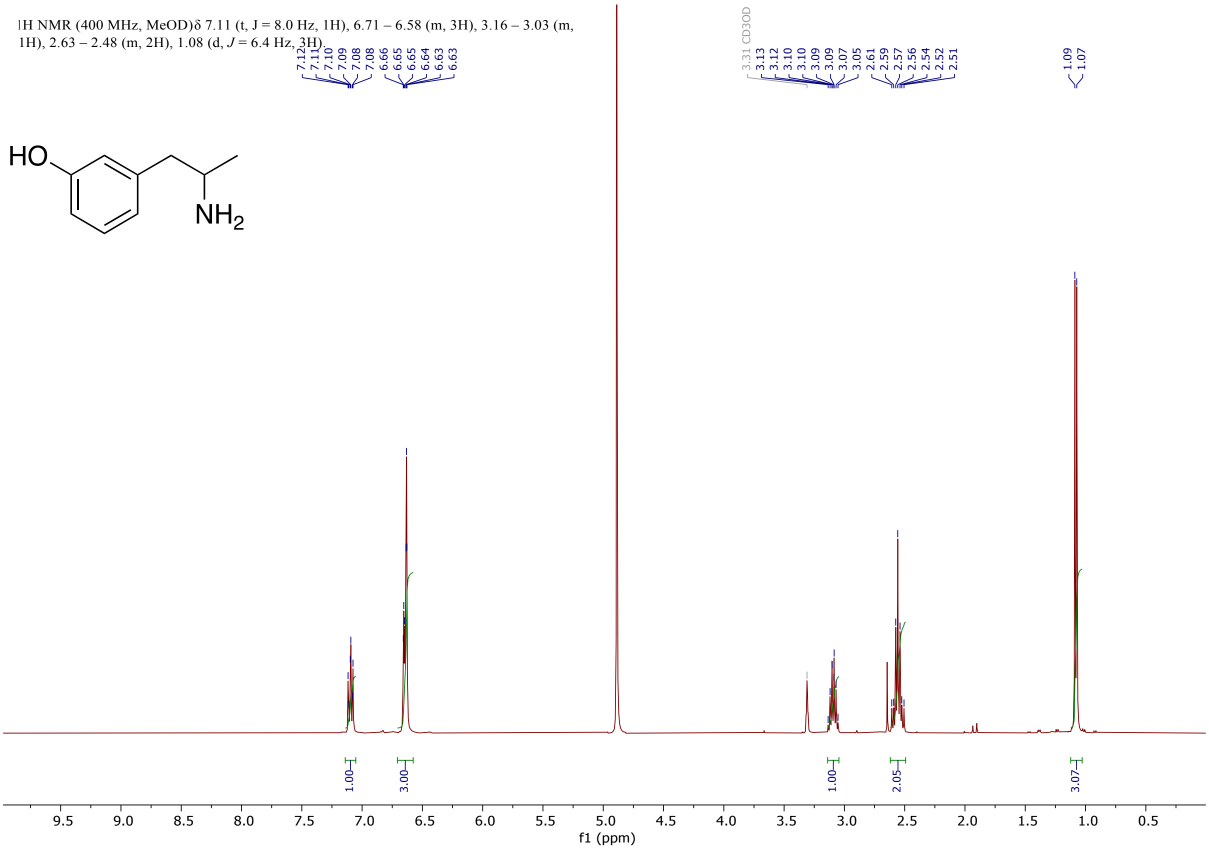


13C NMR (101 MHz, MeOD)δ 158.8, 142.0, 130.5, 121.3, 117.1, 114.4, 49.5, 46.5, 22.4.

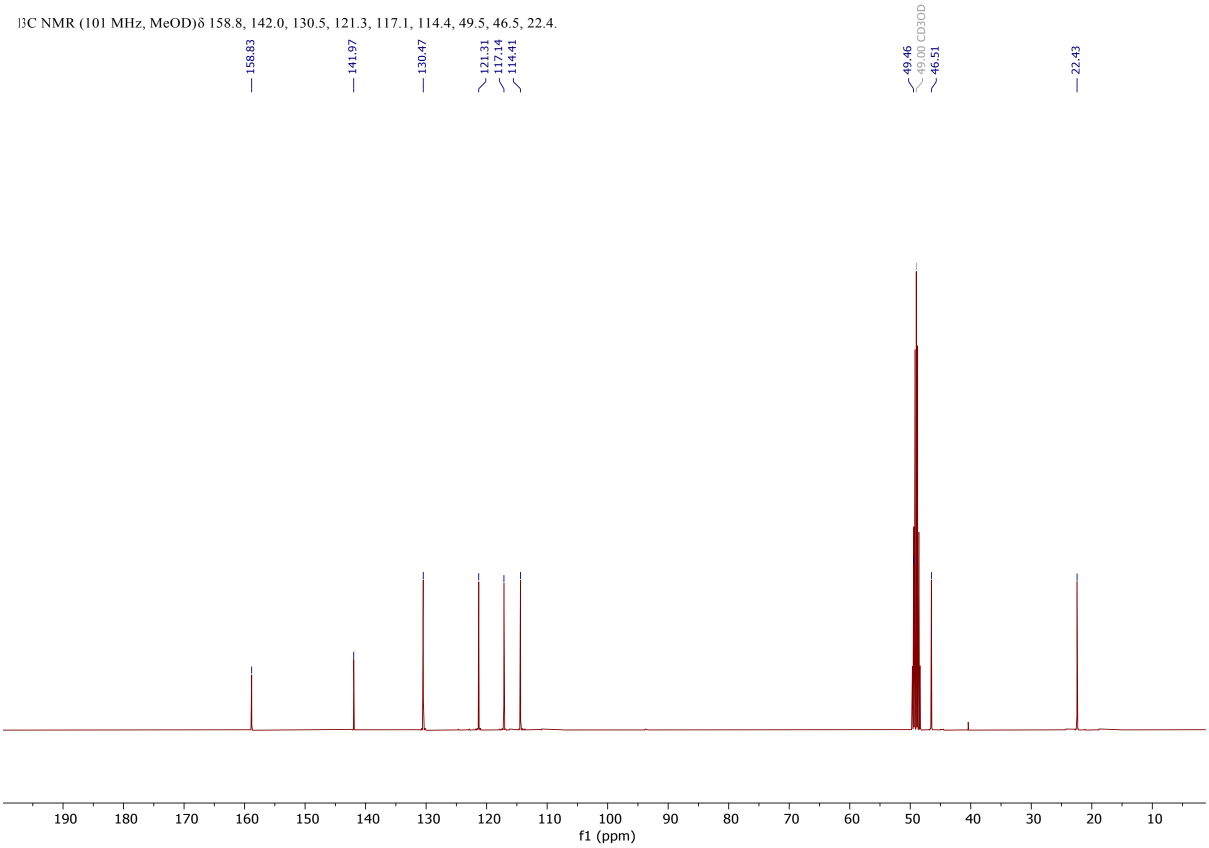

## Appendix

As mentioned briefly in the main text, a range of aldehydes were also tested for the biocatalytic Pictet-Spengler cascade in a manner portrayed in Scheme S1 below. As ketones **8a-c** gave disappointing conversions to the expected THIQ product **11-13**, it was hypothesised that the corresponding aldehydes would be more reactive towards the Pictet-Spengler reaction and better substrates for the transaminase reaction. To this end, the PS reaction was optimised for the reactants **S15** and **S17** and biotransformations were preformed between aldehydes **S16a-b** with amine donors **S14** described in Table S4.

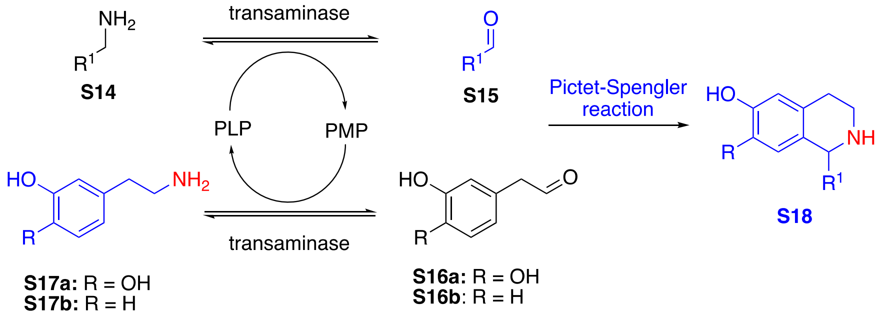


***Scheme S1**: An ATA-Pictet-Spengler cascade demonstrating the concept of biocatalytic amine borrowing for the synthesis of THIQs from aldehyde substrates. The transaminase substrates are shown in black, and the transaminase products are shown in blue.*

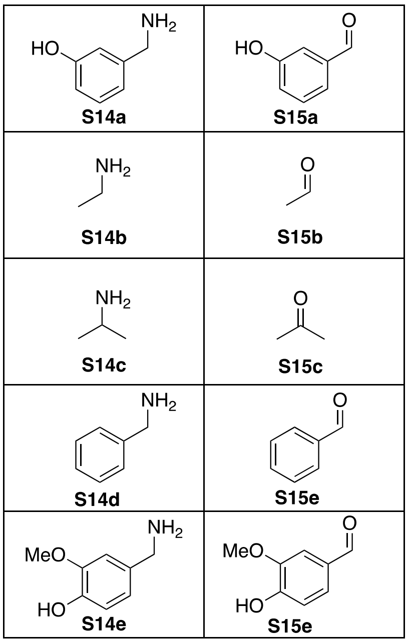


***Figure S2:** Summary of amine donors and their carbonyl equivalents used in TA-PS cascade reactions with aldehydes **S16a,b***

## Procedure for analytical scale Pictet-Spengler reactions with ß-phenylethylamines

A stock solution of 3-(2-aminoethyl)phenol **S17b** (200 mM) in $KP_i$ buffer (100 mM or 300 mM) was prepared and 100 µL was aliquoted equally into microcentrifuge tubes. To this, the aldehyde 3-hydroxybenzaldehyde **S15a** or acetaldehyde **S15b** (from a stock solution in methanol) was added to the tubes. The mixture was pH adjusted and then was brought up to 1000 µL with $KP_i$ buffer and methanol so that the final solution would be 25% *v/v* methanol. The resulting mixture was incubated at 40 °C for 24 h in a shaking incubator (200 rpm), after which 630 µL of the mixture was taken and 70 µL $D_2O$ was added. This sample was analyzed by NMR.

## Table S4. Optimization of phosphate catalysed Pictet-Spengler reaction

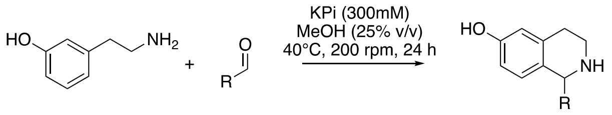


**S17b** **S15a-c** **S18a-c**

| Run | R | Aldehyde (mM) | pH | $KP_i$ (mM) | Temp (°C) | Conversion. (%) |
|---|---|---|---|---|---|---|
| 1 | S15a | 75 | 6 | 100 | 50 | 53 |
| 2 | S15a | 20 | 6 | 100 | 40 | 49 |
| 3 | S15a | 20 | 9 | 100 | 45 | 62 |
| 4 | S15a | 20 | 6 | 300 | 40 | 46 |
| 5 | S15a | 20 | 9 | 300 | 40 | 79 |
| 6 | S15a | 20 | 6 | 300 | 40 | 63 |
| 7 | S15a | 20 | 9 | 100 | 40 | 72 |
| 8 | S15b | 20 | 6 | 100 | 40 | 44 |
| 9 | S15b | 20 | 9 | 300 | 40 | 48 |
| 10 | S15c | 20 | 6 | 100 | 40 | 0 |
| 11 | S15c | 20 | 6 | 300 | 40 | 3 |
| 12 | S15c | 20 | 9 | 300 | 40 | 7.5 |

*Reaction conditions: 3-(2-aminoethyl)phenol **S17b**, aldehyde **S15a-c** (1 equivalent), $KP_i$ buffer (100 or 300mM), methanol (25 % v/v), 200 rpm, 24 h. Conversion was measured by NMR. Results are the mean of two replicates.*

## Analytical scale biotransformations for the synthesis of THIQ (S18) from aldehydes S16a and S16b

Commercially available (S)-selective ATA256 (25mg) was rehydrated in $KP_i$ buffer (5 mL, 300 mM, pH 9) containing PLP (2 mM). The solution was pH adjusted using NaOH solution (1 M). The solution was divided equally into separate microcentrifuge tube (500 µL). To each, a solution of aldehyde substrate (3, 4-dihydroxyphenyl)acetaldehyde **S16a** or 2-(3-hydroxyphenyl)acetaldehyde **S16b** (20 mM, 80 µL from a 250 mM stock solution in methanol) was added, followed by a solution of the corresponding amount of amine donor **S14** (5 equiv., 200 µL from a 500 mM stock solution in $KP_i$ buffer). The total volume was adjusted to 1000 µL and the reaction mixture was incubated at 40 ˚C, 200 rpm, for 24 h. After completion, the pH of each solution was

adjusted to 12 using aq. NaOH (50 μL, 10M) and extracted with EtOAc (1000 μL). The resulting organic layer was analysed by GC-FID *via* gradient **2**.

## Table S5. Results for the ATA-Pictet-Spengler cascade with aldehyde substrates S16a/b

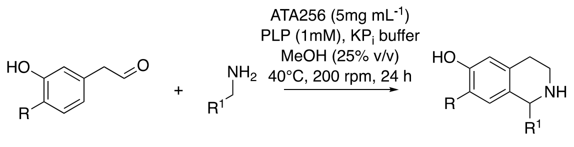


**S16a-b** **S14d-e** **S18d-e**

| Run | R | Amine donor **S14** | pH | Equiv. amine donor | Conversion to THIQ **S18** (%) |
|---|---|---|---|---|---|
| 1 | H | S14d | 9 | 1.1 | n.d |
| 2 | H | S14d | 9 | 5 | n.d |
| 3 | H | S14d | 7 | 5 | <1 |
| 4 | H | S14d | 6 | 5 | 3 |
| 5 | OH | S14e | 9 | 5 | n.d |
| 6 | OH | S14e | 6 | 5 | n.d |

*Reaction conditions:**S16a-b**, amine donor **S14**, ATA256 (5 mg $mL^{-1}$), PLP (1mM) $KP_i$ buffer (100 or 300 mM), methanol (25 % v/v), 40 °C, 200 rpm, 24 h. Conversion was measured by GC-FID (gradient **2**). Results are the mean of two replicates. N.d = none detected.*

## Procedure for the synthesis of aldehydes for the Pictet-Spengler reaction

### 4-(2-hydroxyethyl)benzene-1,2-diol (I-5)

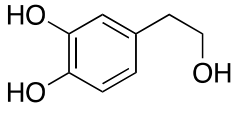


A solution of 3, 4 dihydroxyphenylacetic acid (1.0075 g, 5.9 mmol) in dry THF (30 mL) was added dropwise to a solution of lithium aluminium hydride (1.6g, 39.2 mmol) in dry THF (210 mL). After 10 minutes, the solution was heated to reflux for 2 hours, then cooled to -15 °C and the reaction was quenched by the addition of ethyl acetate (50 mL) and water (10 mL). Solvent was removed by rotary evaporation. The residue was resuspended in water (10 mL) and then acidified to pH 0. The resulting solution was extracted with ethyl acetate (7 x 100 mL). The combined organic layers were washed with saturated sodium bicarbonate until reaching a permanent pH of 8.5. The combined aqueous phases were extracted with ethyl acetate. The final combined organic phases were dried with excess magnesium sulphate and concentrated *in vacuo*, yielding a brown oil (410 mg, 45 %). $^1$H NMR (300 MHz, MeOD) $\delta_H$ 6.72 – 6.65 (2H, m, ArC**H**), 6.53 (1H, dd, $J$ = 8.0, 2.1 Hz, ArC**H**), 3.69 (2H, t, $J$ = 7.2 Hz, C$\mathbf{H_2}$), 2.67 (2H, t, $J$ = 7.2 Hz, C$\mathbf{H_2}$); $^{13}$C NMR (126 MHz, MeOD) $\delta_C$ 146.3, 144.8, 131.9, 121.4, 117.2, 116.5, 64.8, 39.8; HRMS (ESI) m/z: $[M+H]^+$ Calcd. for $C_8H_{11}O_3^+$ 155.0703; Found 155.0703. In accordance with literature data.[5]

**2-(3,4-dihydroxyphenyl)acetaldehyde (S16a)**

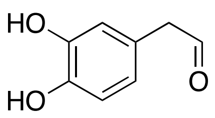

4-(2-hydroxyethyl)benzene-1,2-diol **I-5** (300 mg, 1.9 mmol) was dissolved in 1:1 dry DCM:DMSO (7 mL). To this, *N,N*-diisopropylethylamine (827 µL, 4.75 mmol, 2.5 eq.) was added at –15 °C. A solution of $SO_3$.pyridine in 1:1 DCM:DMSO (7.5 mL) was then added dropwise to the mixture over 30 minutes at –15 °C. The reaction was stirred for 1 hr at –15 °C Reaction was quenched by the addition of ice-cold water (50 mL) and the aqueous layer was extracted with DCM (3 x 50 mL). The combined organic layers were washed with brine (50 mL) and solvent was removed *in vacuo* before purification by column chromatography (eluent cyclohexane:ethyl acetate 85:15) to give a yellow oil (127 mg, 44 %). $^1$H NMR (300 MHz, $CDCl_3$) $\delta_H$ 9.69 (1H, t, *J* = 2.5 Hz, C**H**O), 6.85 (1H, d, *J* = 8.1 Hz, Ar**H**), 6.72 (1H, dd, *J* = 3.8, 2.0 Hz, Ar**H**), 6.62 (1H, dd, *J* = 8.0, 2.1 Hz, Ar**H**), 5.98 (3H, bs, O**H**), 3.57 (2H, d, C**H**$_2$); $^{13}$C NMR (126MHz, $CDCl_3$) $\delta_C$ 186.4, 144.4, 142.9, 130.9, 120.6, 116.9, 115.3, 50.6; HRMS (ESI) m/z: [M-H]$^-$ Calcd. for $C_8H_7O_3^-$ 151.0401; Found 151.0401. In accordance with literature data. [6]

**2-(3-hydroxyphenyl)acetaldehyde (S16b)**

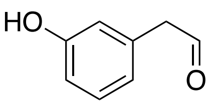

2-(3-hydroxyphenyl)ethanol (470 mg, 3.4 mmol) was dissolved in a solution of dry 1:1 DCM:DMSO (10mL). To this, *N,N*-diisoproylethylamine (1.57 mL, 9.05 mmol, 2.5 equiv.) was added, followed by the dropwise addition of a solution of $SO_3$.pyridine (1.44 g, 9.05 mmol, 2.5 eq.) in dry 1:1 DCM:DMSO (15 mL) over 45 minutes at -15 °C. The solution turned from clear to yellow upon addition of the $SO_3$.pyridine mixture. After 45 minutes, the reaction was quenched by the addition of ice-cold water (50 mL), the aqueous layer was extracted with DCM (3 x 50 mL) and combined organic layers were concentrated *in vacuo*. The crude material was purified by flash chromotography (eluent 20 % EtOAc in cyclohexane) to give the title compound as a yellow oil (87 mg, 19 %). $^1$H NMR (400 MHz, $CDCl_3$) $\delta_H$ 9.71 (1H, t, *J* = 2.4 Hz, C**H**O), 7.22 (1H, t, *J* = 7.8 Hz, 5-Ar**H**), 6.79 – 6.74 (2H, m, 4-Ar**H** and 6-Ar**H**), 6.71 – 6.67 (1H, m, 2-Ar**H**), 6.01 (1H, bs, O**H**), 3.63 (2H, d, *J* = 2.4 Hz, C**H$_2$**); $^{13}$C NMR (101 MHz, $CDCl_3$) $\delta_C$ 200.1, 156.5, 133.4, 130.4, 121.9, 116.7, 114.7, 50.5; HRMS (ESI) m/z: [M-H]$^-$ Calcd. for $C_8H_7O_2^-$ 135.0452; Found 135.0452. In accordance with literature data.[6]

## $^{1}H$ and $^{13}C$ NMR

## 2-(3,4-dihydroxyphenyl)acetaldehyde S16a

$^{1}H$ NMR (300 MHz, $CDCl_3$) $\delta_H$ 9.69 (1H, t, $J$ = 2.5 Hz, C**H**O), 6.85 (1H, d, $J$ = 8.1 Hz, Ar**H**), 6.72 (1H, dd, $J$ = 3.8, 2.0 Hz, Ar**H**), 6.62 (1H, dd, $J$ = 8.0, 2.1 Hz, Ar**H**), 5.98 (3H, bs, O**H**), 3.57 (2H, d, C**H**$_2$)

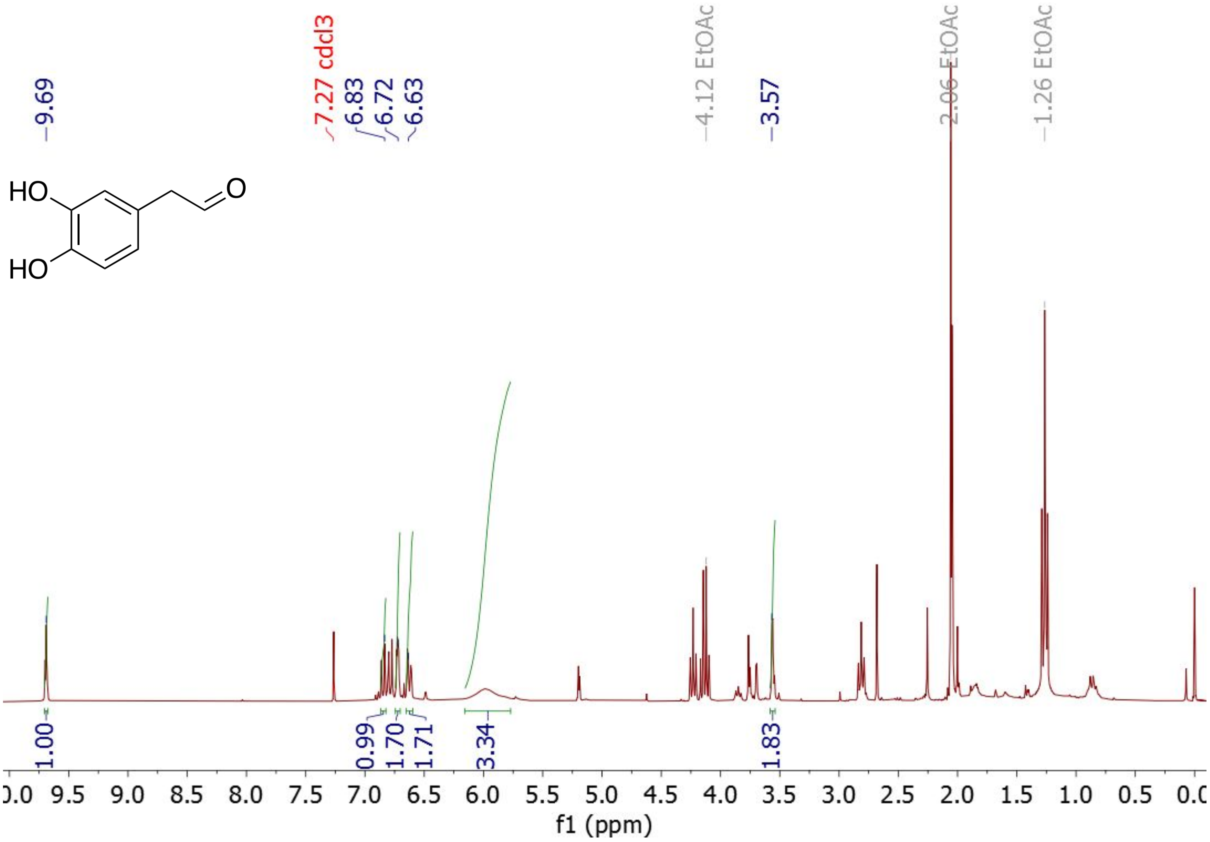


$^{13}C$ NMR (126MHz, $CDCl_3$) $\delta_C$ 186.4, 144.4, 142.9, 130.9, 120.6, 116.0, 115.3, 50.6

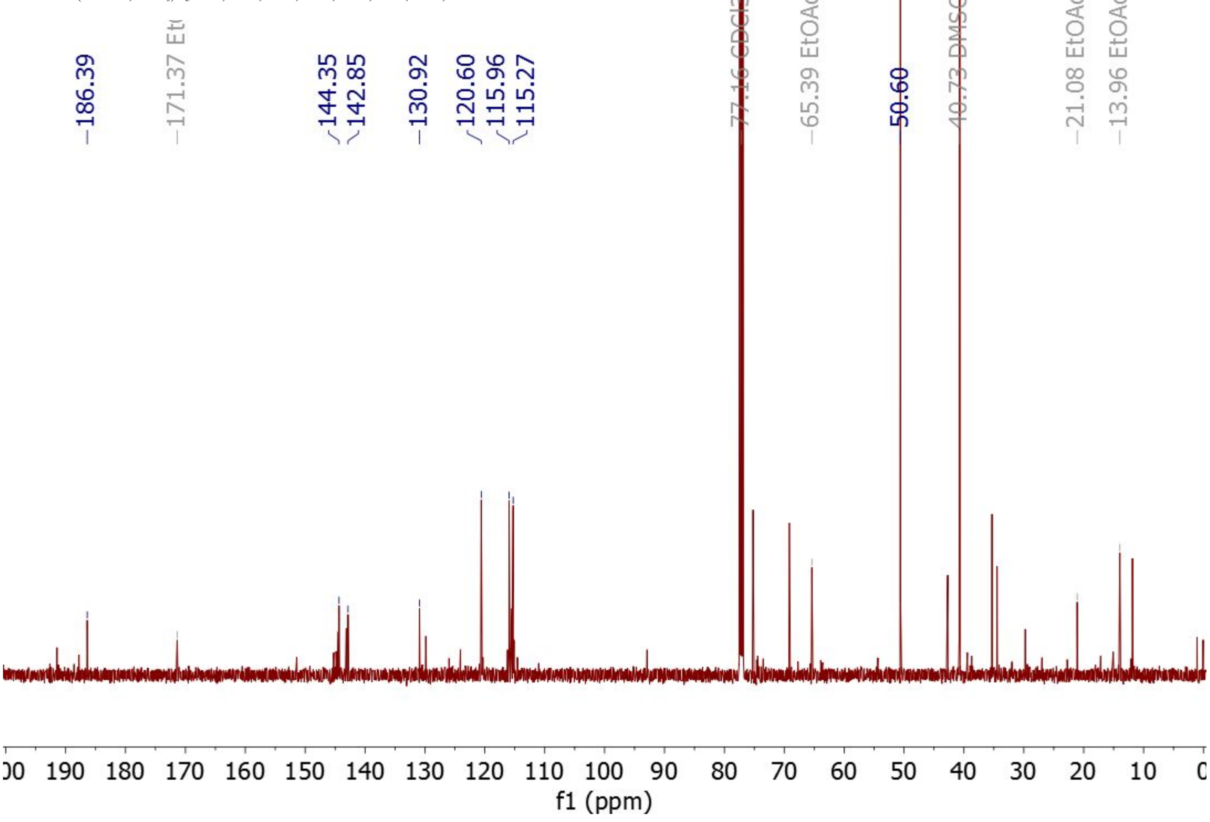

# 2-(3-hydroxyphenyl)acetaldehyde S16b

$^{1}$H NMR (400 MHz, $CDCl_3$) $\delta_H$ 9.71 (1H, t, $J$ = 2.4 Hz, C**H**O), 7.22 (1H, t, $J$ = 7.8 Hz, 5-Ar**H**), 6.79 – 6.74 (2H, m, 4-Ar**H** and 6-Ar**H**), 6.71 – 6.67 (1H, m, 2-Ar**H**), 6.01 (1H, bs, O**H**), 3.63 (2H, d, $J$ = 2.4 Hz, C**H**$_2$)

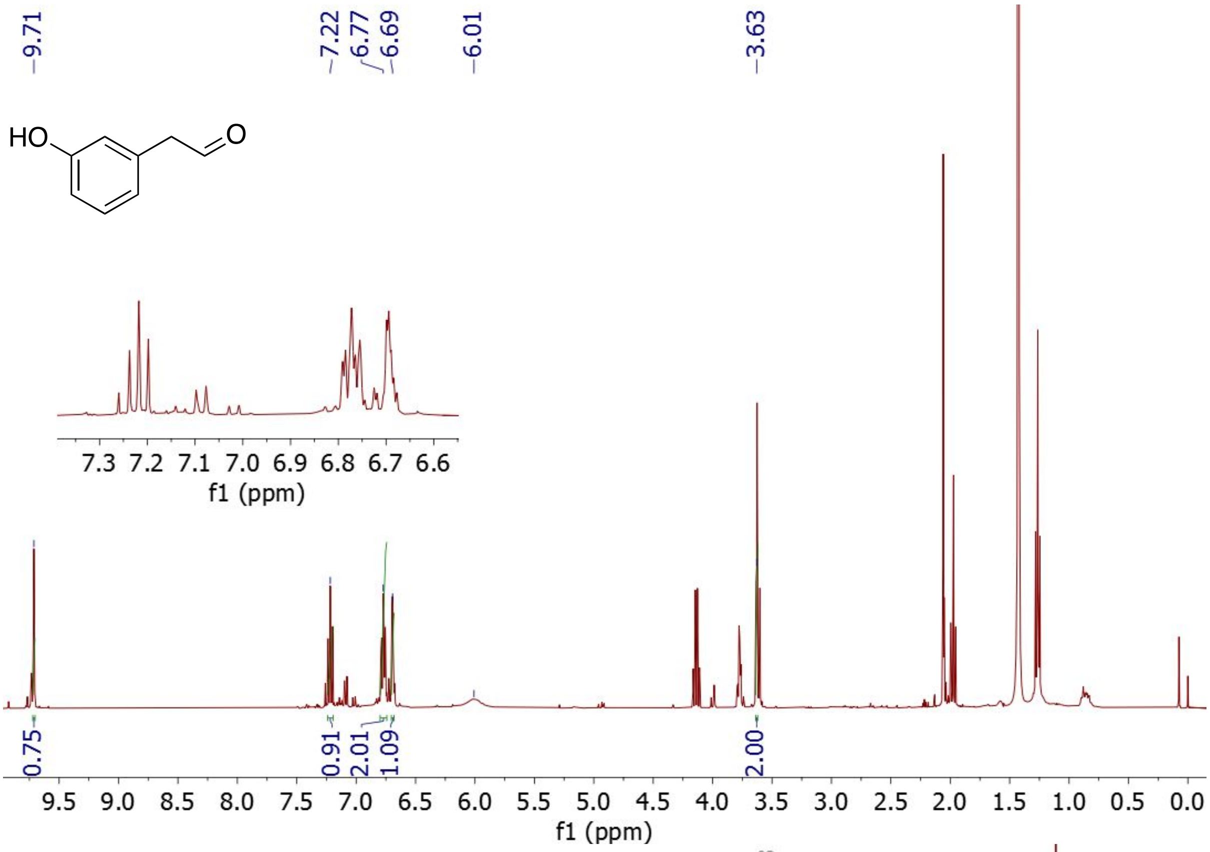


$^{13}$C NMR (101 MHz, $CDCl_3$) $\delta_C$ 200.1, 156.5, 133.4, 130.4, 121.9, 116.7, 114.7, 50.5

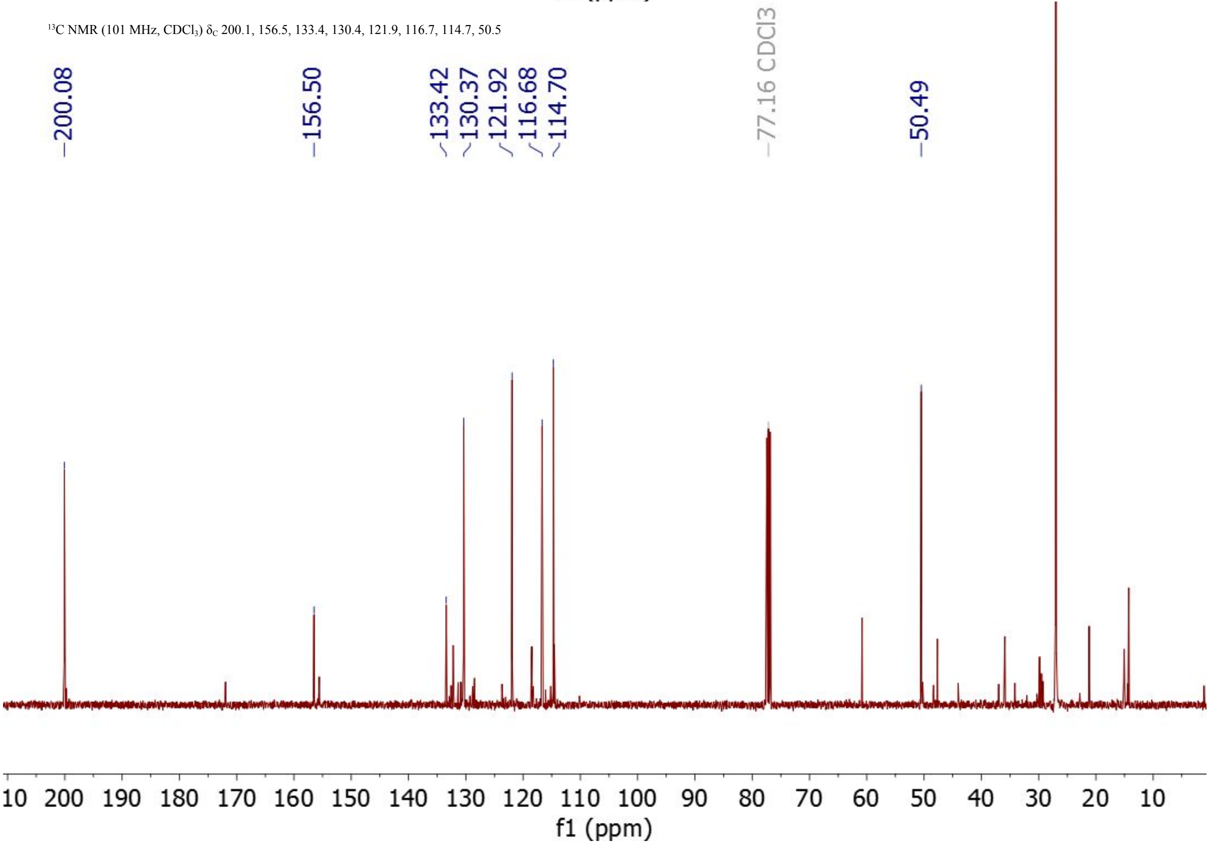

## References


(1) McNulty, J.; Das, P. Development of a One-Pot Method for the Homologation of Aldehydes to Carboxylic Acids. *Tetrahedron* **2009**, *65* (37), 7794–7800. https://doi.org/https://doi.org/10.1016/j.tet.2009.07.032.

(2) Bouchez, L.; Gerbeaux, C.; Rusch, M.; Patoor, M.; Livendahl, M.; Press, N. 'Wake-Up Call of A Sleeping Beauty': Straightforward Synthesis of Functionalized β-(2-Pyridyl) Ketones from 2,6-Lutidine. *Synlett* **2017**, *28*. https://doi.org/10.1055/s-0036-1588154.

(3) Li, P.; Lü, B.; Fu, C.; Ma, S. Zheda-Phos for General α-Monoarylation of Acetone with Aryl Chlorides. *Adv. Synth. Catal.* **2013**, *355* (7), 1255–1259. https://doi.org/https://doi.org/10.1002/adsc.201300207.

(4) Xu, J.; Green, A. P.; Turner, N. J. Chemo-Enzymatic Synthesis of Pyrazines and Pyrroles. *Angew. Chemie - Int. Ed.* **2018**, *57* (51), 16760–16763. https://doi.org/10.1002/anie.201810555.

(5) Capasso, R.; Evidente, A.; Avolio, S.; Solla, F. A Highly Convenient Synthesis of Hydroxytyrosol and Its Recovery from Agricultural Waste Waters. *J. Agric. Food Chem.* **1999**, *47* (4), 1745–1748. https://doi.org/10.1021/jf9809030.

(6) Lichman, B. R.; Lamming, E. D.; Pesnot, T.; Smith, J. M.; Hailes, H. C.; Ward, J. M. One-Pot Triangular Chemoenzymatic Cascades for the Syntheses of Chiral Alkaloids from Dopamine. *Green Chem.* **2015**, *17* (2), 852–855. https://doi.org/10.1039/c4gc02325k.
